# Amphipathic Proline-Rich Cell Penetrating Peptides for Targeting Mitochondria

**DOI:** 10.1021/acschembio.5c00479

**Published:** 2025-08-19

**Authors:** Adeline Schmitt, Helma Wennemers

**Affiliations:** Laboratory of Organic Chemistry, 27219 ETH Zürich, D-CHAB, Vladimir-Prelog-Weg 3, Zürich 8093, Switzerland

## Abstract

Cell-penetrating peptides (CPPs) offer a platform for targeted intracellular delivery. Here, we developed amphipathic oligoprolines for targeting mitochondria. The rigid peptides feature cationic guanidinium and hydrophobic cyclohexyl groups aligned along the edges of the polyproline II (PPII) helical backbone. Systematic variations of the hydrophobicity through C-terminal and backbone modifications provided CPPs with enhanced cellular uptake and mitochondrial selectivity. Comparative studies with conformationally more flexible analogs revealed the benefit of aligned cationic and hydrophobic residues on a rigid backbone for mitochondria targeting. Notably, the amphipathic peptides undergo time-dependent intracellular redistribution, leading to selective and prolonged mitochondrial residency. Our findings established design principles for optimizing CPPs to target mitochondria.

## Graphical abstract

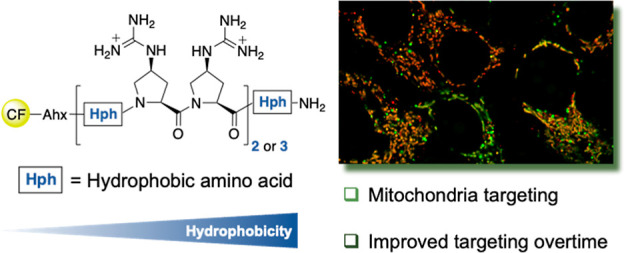

## Introduction

Cell-penetrating peptides (CPPs) are short peptides that cross the plasma membrane of mammalian cells.^1–5^ Their ability to transport cargo such as small molecules,^6^ nucleic acids,^7,8^ and proteins,^9^ has sparked growing interest in the use of CPPs for drug delivery, gene therapy, and molecular imaging.^10–12^ Like other peptides, most CPPs are easily accessible by chemical synthesis. Their modular structure offers a platform for facile modifications and the fine-tuning of the amino acid sequence and, thereby the properties to enhance cellular uptake and selective subcellular targeting.^13–16^ This adaptability has driven extensive research into the development of CPPs for targeting organelles that play key roles in metabolism, such as mitochondria.

Mitochondria are responsible for pivotal cellular functions, including oxidative phosphorylation, the regulation of reactive oxygen species (ROS), and programmed cell death.^17–19^ Mitochondrial dysfunction is, therefore, closely associated with diseases such as Parkinson’s disease,^20,21^ Alzheimer’s disease,^22,23^ diabetes,^24^ and cancer.^25^ Mitochondria targeting can be a first step toward disease treatment. The inner mitochondrial membrane presents, however, a formidable barrier due to its densely packed hydrophobic proteins and a high negative electrochemical gradient.^26^ As a result, the targeting of mitochondria is difficult.^27^

Despite these challenges, certain molecules accumulate selectively in mitochondria.^28,29^ These include lipophilic cations, such as triphenylphosphonium (TPP) moieties,^30,31^ and amphipathic peptides.^32^ Among the latter, lead examples are short peptides with alternating hydrophobic cyclohexylalanine and positively charged arginine (Arg) residues introduced by Kelley that build on early work with amphiphilic tetrapeptides by Szeto and Schiller (Figure 1A,B).^33–39^ Similarly, Chmielewski incorporated hydroxyproline derivatives with isopropyl and guanidinium groups tethered to an alkyl spacer along the backbone of an oligoproline helix to target mitochondria (Figure 1C).^40,41^ Recently, the group used (4*S*)-guanidiniumproline (Gup, Z), with the cationic group directly attached to the backbone instead of via a short alkyl spacer (Figure 1D).^42^ This modification increased the cellular uptake significantly, but the amphipathic peptides no longer targeted mitochondria. These results underscore the challenges of directing peptides to specific organelles.

**Figure 1:**
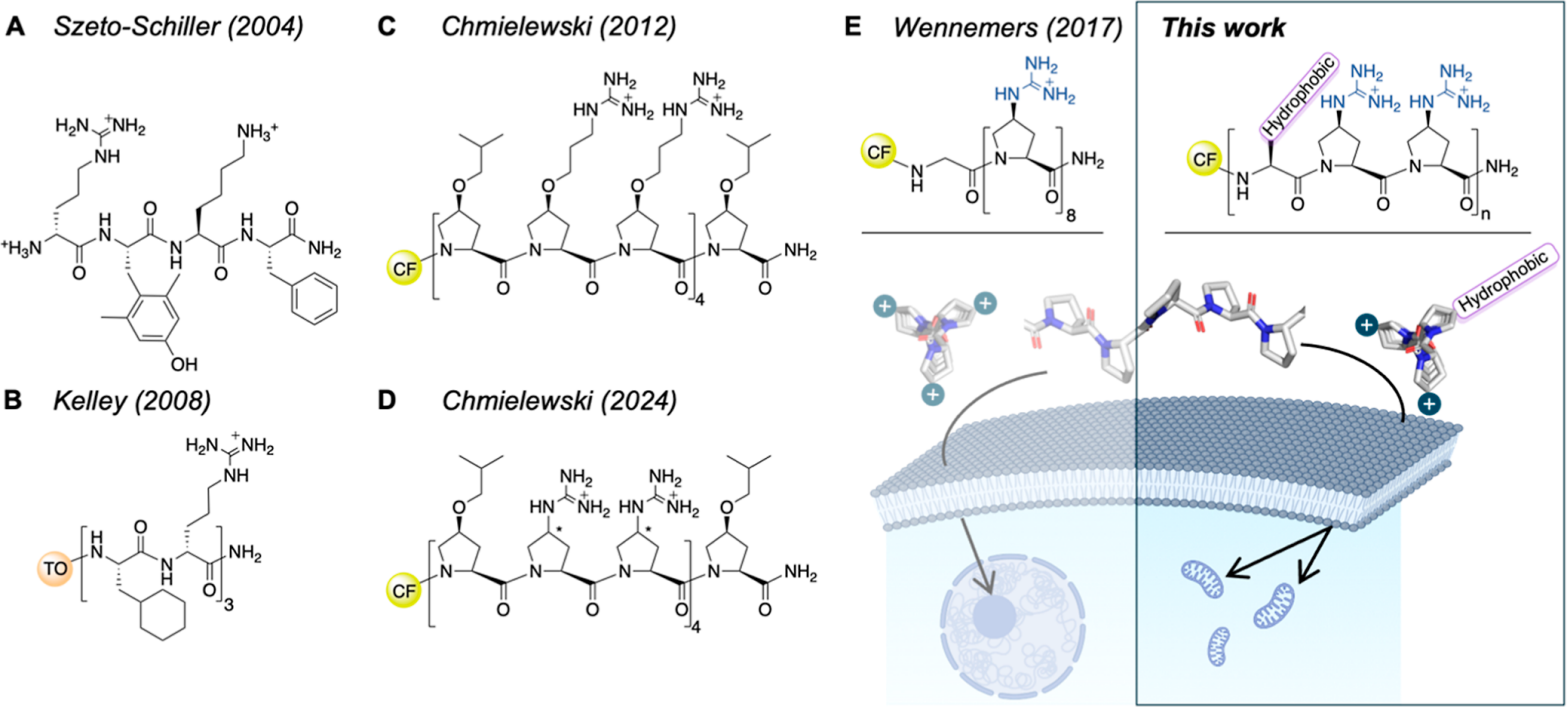
Examples of amphipathic and cationic CPPs. (A) Early example of mitochondria targeting with a synthetic tetrapeptide.^38^ (B) Cha- and dArg-containing mitochondria targeting hexapeptide.^34^ (C) PPII-helical amphipathic 13-mer for mitochondria targeting.^41^ (D) PPII-helical Gup-containing amphipathic CPP does not target mitochondria.^42^ (E) Left: PPII-helical cationic Gup-based oligoproline, Z_8_, targeting the nucleoli.^43^ Right: Amphipathic Gup-based peptides for targeting mitochondria. Center: Structure of a PPII helical oligoproline, view along the axis and side view. CF = 5(6)-carboxyfluorescein; TO = thiazole orange; Gup = (4*S*)-guanidinium-proline.

Our group showed that an oligoproline consisting of eight Gup residues is a potent CPP (Figure 1E, left).^43^ Octa-Gup adopts a polyproline II (PPII) helix in which every third amino acid is aligned (Figure 1E, center). As a result, the charges are organized along the three edges of the helix. The peptide localizes in the nucleoli and the cytoplasm with minimal endosomal entrapment, thereby overcoming one of the major challenges in the CPP field. In contrast, more flexible analogs—octaarginine and an oligoproline with spacers between the proline backbone and the guanidinium group—are trapped in endosomes to a significant extent and exhibit reduced cellular uptake.^43^ Leveraging the advantageous cellular uptake properties observed with the Gup-containing peptide, we aimed to develop and gain a better understanding of the features necessary for amphipathic Gup-containing peptides to target mitochondria selectively.

Herein, we show that PPII-helical peptides with amphipathic patterning achieve mitochondrial targeting. Hydrophobicity tuning—at the C-terminus or at the backbone—modulates cellular uptake and selectivity. Notably, the peptides redistribute over time within cells, enhancing and prolonging mitochondrial localization.

## Results and Discussion

### Effect of Different Hydrophobic Side Chains on the Intracellular Delivery of Amphipathic Gup-Containing Peptides

We started by investigating the effect of the side chain of hydrophobic amino acids on the intracellular localization of Gup-rich peptides. A set of peptides bearing amino acids with a hydrophobic side chain in every third position (X) of a 9-mer (XZZ)_3_ (Z = Gup) was synthesized by solid phase peptide synthesis (SPPS). Specifically, we incorporated valine (Val), phenylalanine (Phe), tryptophan (Trp), and cyclohexylalanine (Cha). 5(6)-Carboxyfluorescein (CF) served as a label for intracellular visualization and aminohexanoic acid (Ahx) as a spacer between the amphipathic peptide and the fluorophore, CF-Ahx-(XZZ)_3_-NH_2_ (Figure 2A).

**Figure 2:**
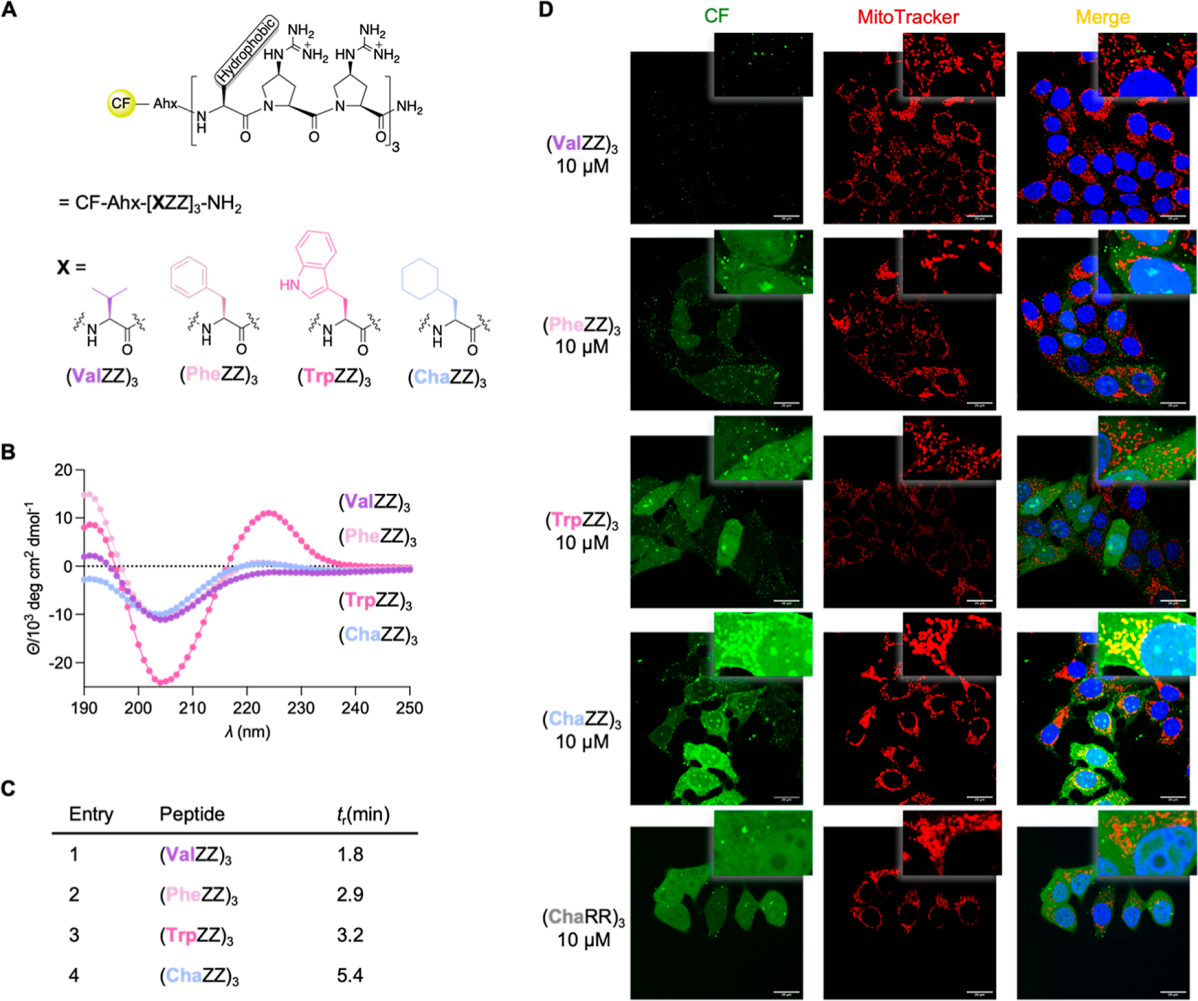
(A) General structure of amphipathic peptides, CF-Ahx-[XZZ]_3_-NH_2_; CF = 5(6)-Carboxyfluorescein, Ahx = aminohexanoic acid. (B) CD spectra of the peptides, recorded at 50 μM in H_2_O, pH 5.7. For a comparison with a spectrum of a Gup oligomer, see Figure S2. (C) Retention time of the peptides measured by RP-HPLC on a C4 column with a mobile phase from 30 to 55% of MeCN in H_2_O/MeCN/TFA (1000/10/1). (D) Representative confocal microscopic images of live MCF-7 cells, incubated for 1 h at 37 °C with peptide solutions at a concentration of 10 μM in DMEM + 1% FBS (left, in green). Mitochondria were stained with Deep Red MitoTracker (middle, in red) and merged images (right, orange/yellow indicating colocalization) with Hoechst33342 stain (blue) for the nucleus.

The Gup residues should preorganize the peptides into a PPII helix with one hydrophobic and two cationic edges along the PPII helical peptide. Indeed, circular dichroism (CD) spectra of the peptides display the characteristic minimum and maximum at ∼206 nm and ∼224 nm, respectively, of a PPII helix (Figure 2B). The spectra imply that the non-proline residues in every third position are tolerated within the helical structure.^44^ We used reverse phase high performance liquid chromatography (RP-HPLC) for an estimate of the hydrophobicity of the peptides. On a C4 column using two mobile phases A (H_2_O/MeCN/TFA (1000/10/1)) and B (MeCN) with a gradient of 30% to 55% B, all peptides eluted over 20 min but with different retention times (Figure 2C). The Cha-containing peptide had the longest retention time, indicating the highest hydrophobicity within the series, followed by the Trp-, Phe-, and Val-peptides.

To explore the cellular uptake and intracellular localization, we incubated the amphipathic peptides (10 μM) with breast cancer cells (MCF-7) for 1 h at 37 °C. MitoTracker Deep Red was used as a counter stain for mitochondria and Hoechst 33342 for the nucleus. Inspection of the cells by confocal microscopy showed little cellular uptake of the Val peptide, even at a concentration of 20 μM; the Phe and Trp peptides were detected in the cytosol and the nucleoli but not in the mitochondria (Figure 2D). The most hydrophobic peptide with Cha residues localized in the mitochondria as well as in the cytoplasm and the nucleoli. To explore whether PPII-helicity is key for mitochondrial localization, we prepared peptide CF-Ahx-(ChaRR)_3_-NH_2_ with Arg instead of Gup residues. This control peptide features a flexible conformation as judged by CD spectroscopy (Figure S1B). Cellular studies monitored by confocal microscopy indicate high uptake into the cytoplasm and the nucleus, but little to no uptake into mitochondria (Figure 2D). These findings show the importance of the secondary structure of the CPP for subcellular organelle localization.

### Modulating the Hydrophobicity of the Cha-Peptide to Enhance Mitochondria Targeting

To explore the importance of hydrophobic moieties for cellular uptake and mitochondria targeting further, we added a Cha residue at the C-terminus. In addition, we explored analogs bearing a cyclohexyl moiety directly attached at C^γ^ of the oligoproline backbone.

#### Effect of an Additional Cha Residue at the C-Terminus

The CPPs of the initial series feature a cationic Gup residue at the C-terminus. We envisioned that the addition of a hydrophobic C-terminal Cha residue could modulate the uptake and mitochondria targeting properties and prepared CF-Ahx-(ChaZZ)_3_-Cha-NH_2_ for comparison with CF-Ahx-(ChaZZ)_3_-NH_2_. We also prepared shorter analogs, CF-Ahx-(ChaZZ)_2_-NH_2_ and CF-Ahx-(ChaZZ)_2_-Cha-NH_2_, bearing only 6 and 7 amino acids, respectively (Figure 3A). CD spectra confirmed that neither the additional Cha residue nor the shortening of the peptides disturbed the preferential PPII helicity (Figure 3B). Thus, the two cationic and one hydrophobic edges are retained in this second series of peptides. RP-HPLC analyses imply that the shorter peptides are less hydrophobic compared to their longer analogs (Figure 3C). The additional C-terminal Cha residue increased the hydrophobicity significantly as indicated by a higher retention time of ∼3 min.

**Figure 3:**
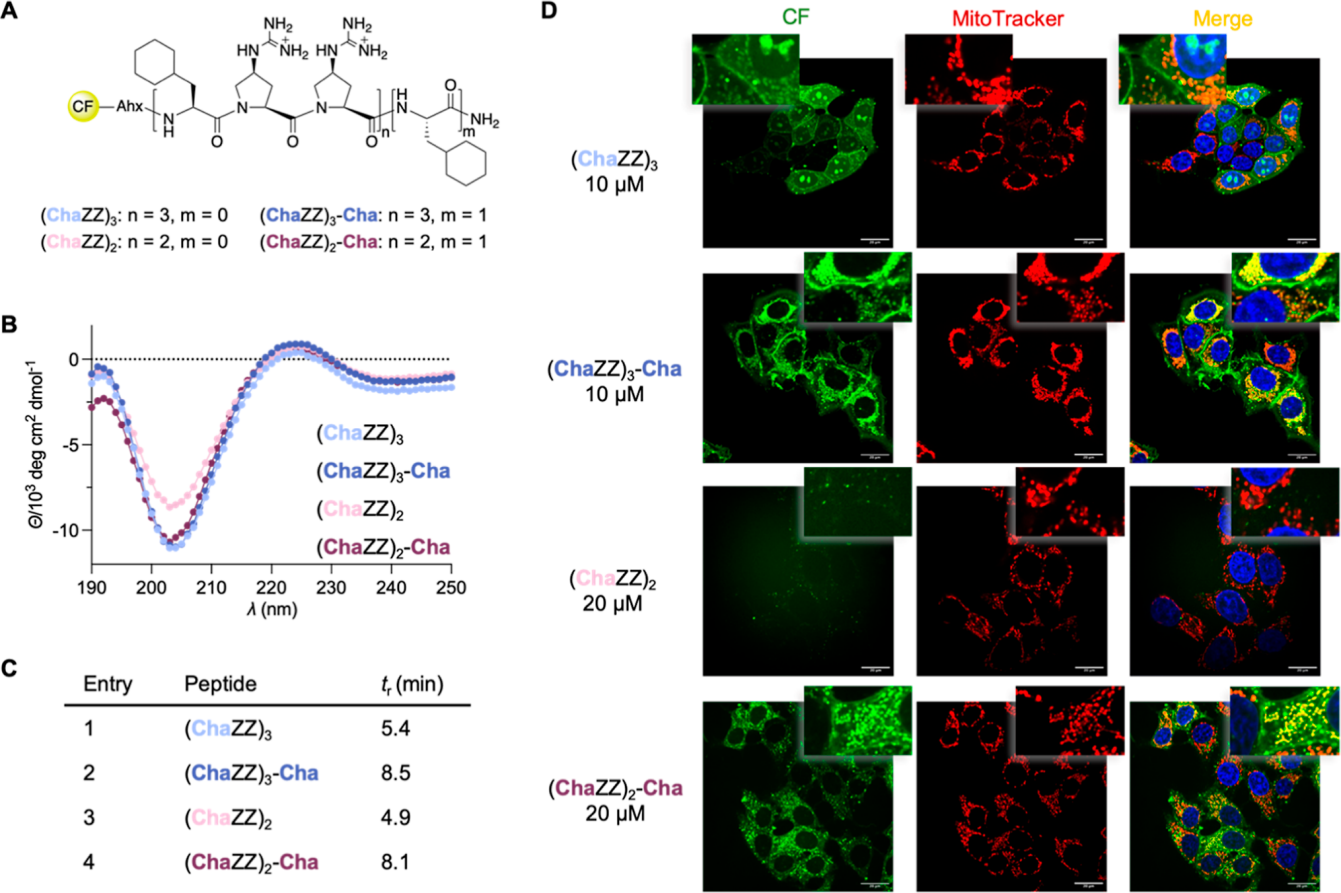
(A) 2nd set of peptides with varied lengths and additional C-terminal Cha residue; CF = 5(6)-Carboxyfluorescein, Ahx = aminohexanoic acid. (B) CD spectra of the peptides, recorded at 50 μM in H_2_O, pH 5.7. (C) Retention time of the CF-Ahx-peptides measured by RP-HPLC on a C4 column with a mobile phase from 30 to 55% of MeCN in H_2_O/MeCN/TFA (1000/10/1). (D) Representative confocal microscopy images of live MCF-7 cells after incubation with the peptides for 1 h at 37 °C at different concentrations in DMEM + 1% FBS (left, green). Middle: Mitochondria were stained with Deep Red MitoTracker (red). Right: Merged images (orange/yellow indicates colocalization) and staining of the nucleus with Hoechst33342 stain (blue).

The cellular uptake and mitochondrial targeting of the peptides were then evaluated with MCF-7 cells (1 h incubation at 37 °C) at different concentrations of the CPPs (5 μM, 10 μM, and 20 μM) with mitochondria and nucleus counterstains (Figures 3D and S3). The lead peptide from the first series, **(ChaZZ)**_**3**_, localized in the cytoplasm, the nucleoli, and the mitochondria at concentrations of 10 μM and higher (Figure 3D). An additional C-terminal Cha residue, **(ChaZZ)**_**3**_ **-Cha**, increased the cellular uptake, as indicated by the intracellular localization already upon incubation at 5 μM, a concentration at which no fluorescence was visible with the parent peptide **(ChaZZ)**_**3**_ (Figure S3) **(ChaZZ)**_**3**_ **-Cha** localized in the mitochondria and not the nucleus but was also trapped in endosomes as indicated by punctuated fluorescence in the cytoplasm (Figure 3D). The shorter variant, **(ChaZZ)**_**2**_, did not penetrate into the cells, even at a concentration of 20 μM. In contrast, the 7-mer analog with an additional C-terminal Cha residue, **(ChaZZ)**_**2**_ **-Cha**, exhibited strong mitochondrial colocalization at 20 μM (Figure 3D) with limited endosomal entrapment. These findings underline the importance of hydrophobicity for selective mitochondria targeting. The data also highlight the beneficial role of a hydrophobic C-terminal residue for translocation of CPPs into cells. This finding is in line with previous work that used hydrophobicity to enhance cellular uptake.^45–49^

#### Effect of Cyclohexyl Moieties Attached at the γ-position of Proline Residues

Next, we explored whether cyclohexyl moieties directly attached to C^γ^ of the oligoproline backbone alter the cell penetrating and targeting properties. In contrast to the peptides of the first and second series that contain secondary amide bonds, the (4*S*)-cyclohexyl-proline (ChPro) containing peptides consist of all-tertiary amide bonds, which should increase hydrophobicity and thereby cellular uptake.^50^ For a direct comparison with the peptides of the first two series, we prepared 6- and 7-mers as well as 9- and 10-mers bearing ChPro in place of Cha residues (Figure 4A). The CD spectra of these peptides are typical for PPII helices (Figure 4B). Their intensity is, in general, more pronounced compared to those of the related Cha-containing peptides at the same concentration, indicating a higher PPII helix propensity. RP-HPLC analyses revealed a longer retention time of 3 min of **(ChProZZ)**_**2**_ compared to **(ChaZZ)**_**2**_ and an even greater shift of 4 min of **(ChProZZ)**_**3**_ **-ChPro** compared to **(ChaZZ)**_**3**_ **-Cha** (Figure 4C).

**Figure 4:**
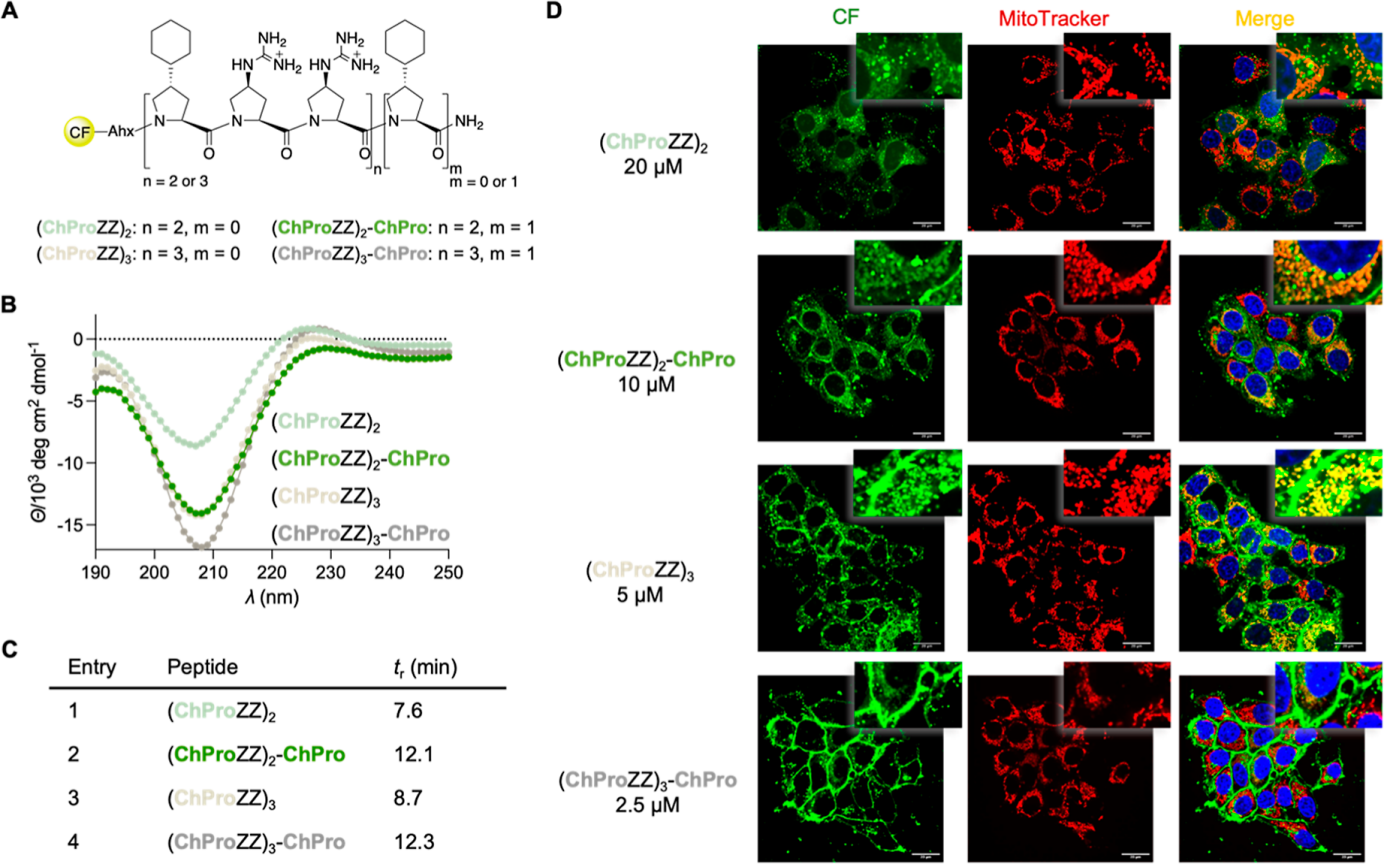
(A) 3rd set of peptides with ChPro instead of Cha residues.; CF = 5(6)-Carboxyfluorescein, Ahx = aminohexanoic acid. (B) CD spectra of the peptides, recorded at 50 μM in H_2_O, pH 5.7. (C) Retention time of the peptides measured by RP-HPLC on a C4 column with a mobile phase from 30 to 55% of MeCN in H_2_O/MeCN/TFA (1000/10/1). (D) Representative confocal microscopy images of live MCF-7 cells after incubation with the peptides for 1 h at 37 °C at different concentrations in DMEM + 1% FBS (left, green). Middle: Mitochondria were stained with Deep Red MitoTracker (red). Right: Merged images (orange/yellow indicates colocalization) and staining of the nucleus with Hoechst33342 stain (blue).

Confocal microscopic imaging of uptake studies with MCF-7 cells showed internalization, even of the shortest variant, **(ChProZZ)**_**2**_, after an incubation time of 1 h at 20 μM (Figure 4D). Under the same conditions, intracellular fluorescence was not detected with the Cha analog (Figures 3D and S3). With the analog bearing an additional C-terminal ChPro, **(ChProZZ)**_**2**_ **-ChPro**, mitochondrial targeting was observed starting at a concentration of 10 μM (Figures 4D and S3). This peptide was also endosomally entrapped as evidenced by punctuated fluorescence in the cytoplasm and localized in the plasma membrane (Figures 4D, S3 and S4). This finding indicates that the hydrophobic C-terminus enhances anchoring of the peptide in the membrane. The longer variant **(ChProZZ)**_**3**_ targeted mitochondria already at a concentration of 5 μM whereas the Cha-containing analog exhibited minimal fluorescence at this concentration (Figure S3). **(ChProZZ)**_**3**_ localized in endosomes and in the cellular membrane. The 10-mer with an additional C-terminal ChPro residue, **(ChProZZ)**_**3**_ **-ChPro**, localized predominantly in the cell membrane, with detectable fluorescence at a concentration as low as 2.5 μM, but featured limited mitochondria localization after incubation for 1 h (Figure 4D). These results suggest that an increased number of ChPro residues enhances membrane binding and restricts intracellular translocation. The high cellular uptake and mitochondrial targeting of the ChPro-containing peptides were associated with cytotoxicity at concentrations lower than those of the other examined peptides. For example, **(ChProZZ)**_**3**_ was cytotoxic at a concentration of 10 μM, whereas **(ChaZZ)**_**3**_ was not, as indicated by altered cell morphology and MitoTracker leakage in confocal imaging (Figure S3). The cytotoxicity is likely caused by too high mitochondrial accumulation of the peptides at the comparatively high concentration used for incubation, resulting in mitochondrial damage and apoptosis. MTT assays at different concentrations quantified the cytotoxicity of the peptides (Figure S5). Based on these studies, concentrations at which the peptides are cytotoxic were excluded from subsequent studies.

### Quantification by Flow Cytometry

Next, we evaluated the overall cellular uptake of the peptides by fluorescence-activated cell sorting (FACS). Here, we focused, particularly, on the effect of a C-terminal hydrophobic residue and the substitution of Cha with ChPro. The cellular fluorescence was quantified after a 1 h-incubation time at various concentrations. As anticipated based on the confocal images, the FACS analyses revealed that an additional C-terminal hydrophobic residue enhances cellular uptake in general, with increases ranging from 1.5- to 13-fold (Figure 5A). The addition of a C-terminal Cha residue to **(ChaZZ)**_**3**_ improved cellular uptake by 3-fold at 5 and 10 μM and 1.5-fold at 20 μM (**(ChaZZ)**_**3**_ **-Cha**; Figure 5A, blue bars). For the shorter 6- to 7-mer analogs, which exhibited minimal intracellular fluorescence below 20 μM (Figure S3), the addition of Cha still led to a 2-fold higher uptake at 5 μM and a 4-fold increase at 10 μM (Figure 5A, purple bars). At 20 μM, the uptake of **(ChaZZ)**_**2**_ **-Cha** is 6-fold higher compared to **(ChaZZ)**_**2**_, with strong mitochondrial localization as seen by confocal microscopy. Similarly, **(ChProZZ)**_**3**_ **-ChPro** is taken up 6-fold more compared to **(ChProZZ)**_**3**_ at 2.5 μM (Figure 5A, gray bars). However, the confocal images show predominant membrane staining at this concentration. Thus, the high uptake of these peptides arises in part from plasma membrane association rather than intracellular localization. The shorter analog **(ChProZZ)**_**2**_ **-ChPro** displayed an exceptional 12- to 13-fold increase in uptake at 5 and 10 μM compared to **(ChProZZ)**_**2**_ (Figure 5A, green bars).

**Figure 5:**
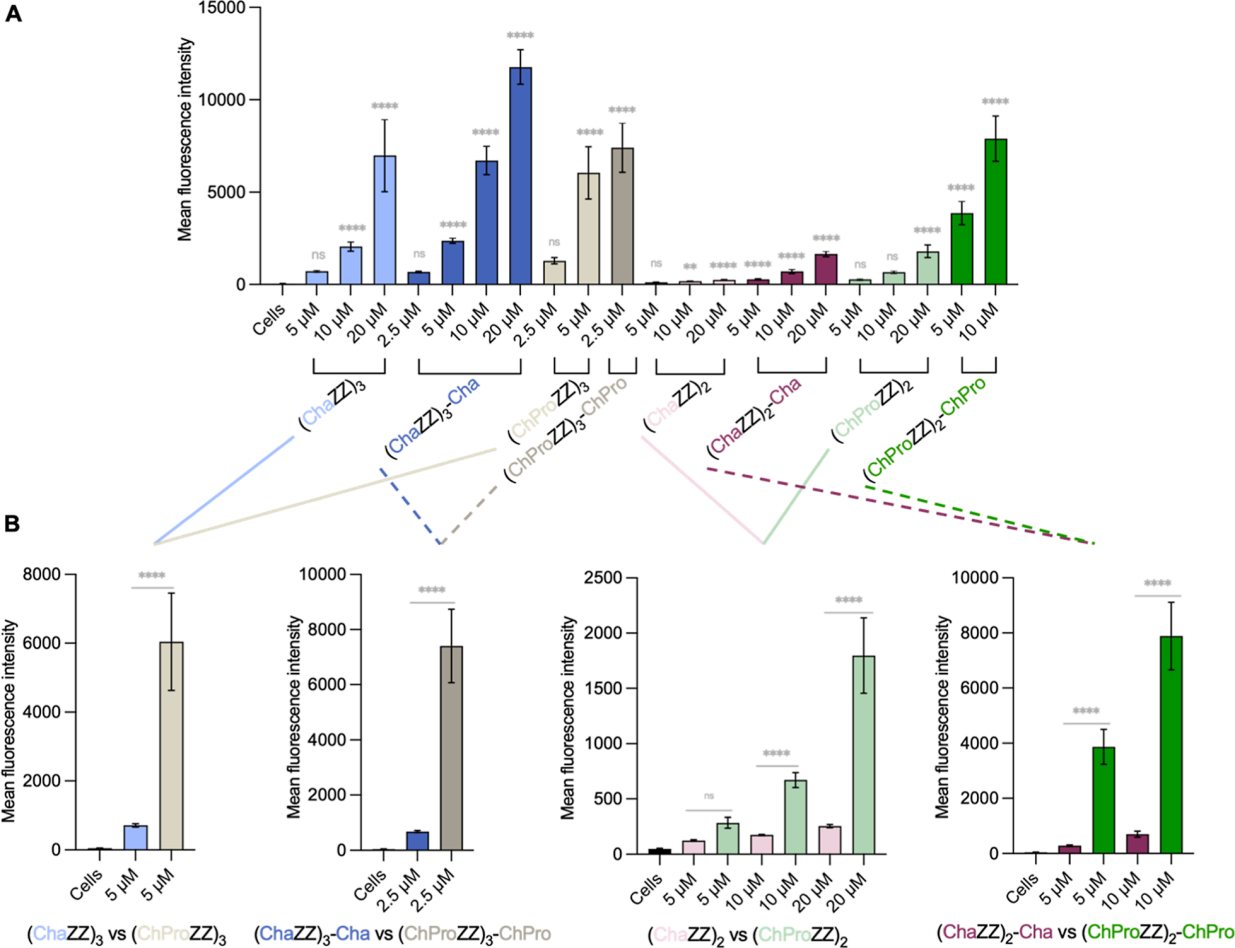
(A) FACS analyses after incubating MCF-7 cells for 1 h at 37 °C with the peptides at different concentrations. The comparison of the effect of C-terminal modifications is represented by color: (ChaZZ)_3_ versus (ChaZZ)_3_-Cha in blue, (ChProZZ)_3_ versus (ChProZZ)_3_-ChPro in gray, (ChaZZ)_2_ versus (ChaZZ)_2_-Cha in purple, (ChProZZ)_2_ versus (ChProZZ)_2_-ChPro in green. (B) Comparison of the cellular uptake when Cha is replaced by ChPro. The indicated *P*-values were determined using one-way ANOVA followed by Tukey’s multiple comparisons test per group of peptides (0.1234 (ns), 0.03328­(*), 0.0021 (**), 0.0002 (***), < 0.0001­(****)).

The replacement of Cha with ChPro, which increases hydrophobicity, led to even more pronounced effects. In case of 9-mer **(ChaZZ)**_**3**_, this seemingly small modification resulted in an 8-fold higher cellular uptake, enabling **(ChProZZ)**_**3**_ to internalize at a concentration of 5 μM (Figure 5B, light blue and gray bars). In case of the 10-mers, the ChPro analog **(ChProZZ)**_**3**_ **-ChPro** internalized 10-fold more compared to the Cha peptide (**ChaZZ)**_**3**_ **-Cha** at 2.5 μM (Figure 5B, dark gray and blue bars). Among the 6-mers, the uptake of **(ChProZZ)**_**2**_ is 2- to 3-fold higher compared to that of **(ChaZZ)**_**2**_ at 5 and 10 μM, and 7-fold higher at 20 μM (Figure 5B, light pink and green bars). Finally, **(ChProZZ)**_**2**_ **-ChPro** exhibited a greater than 10-fold higher uptake at 5 and 10 μM compared to **(ChaZZ)**_**2**_ **-Cha** (Figure 5B, dark purple and green bars), further highlighting the significant impact of ChPro on cell penetration.

Flow cytometry provides the overall fluorescence of the cells, which is a combination of peptides localized in the mitochondria, endosomes, and the cell membrane. Thus, for interpreting the data, the results from confocal imaging (Figures 3, 4, S3 and S4) *and* FACS need to be taken into account. Higher total cellular uptake does not necessarily coincide with higher mitochondrial targeting. Indeed, the microscopic analyses showed that replacing Cha with ChPro made the peptides more prone to endosomal entrapment and localization in the plasma membrane (Figures 3 and 4).

### Intracellular Trafficking of the Peptides over Time Improves Mitochondria Targeting

The observed localization of the ChPro-containing peptides in the plasma membrane let us hypothesize that their cellular localization could change over time. We, therefore, monitored the cellular localization of **(ChProZZ)**_**3**_ **-ChPro** and **(ChProZZ)**_**3**_, the peptides that localized to a large extend in the membrane and endosomes, over time. After incubation for 1 h at 2.5 μM, both peptides localized predominantly in the cell membrane and endosomes (Figures 4D and 6A, left). Afterward, the cells were kept in DMEM supplemented with 10% FBS for 24 h and were then analyzed again (Figure 6A, right).

**Figure 6:**
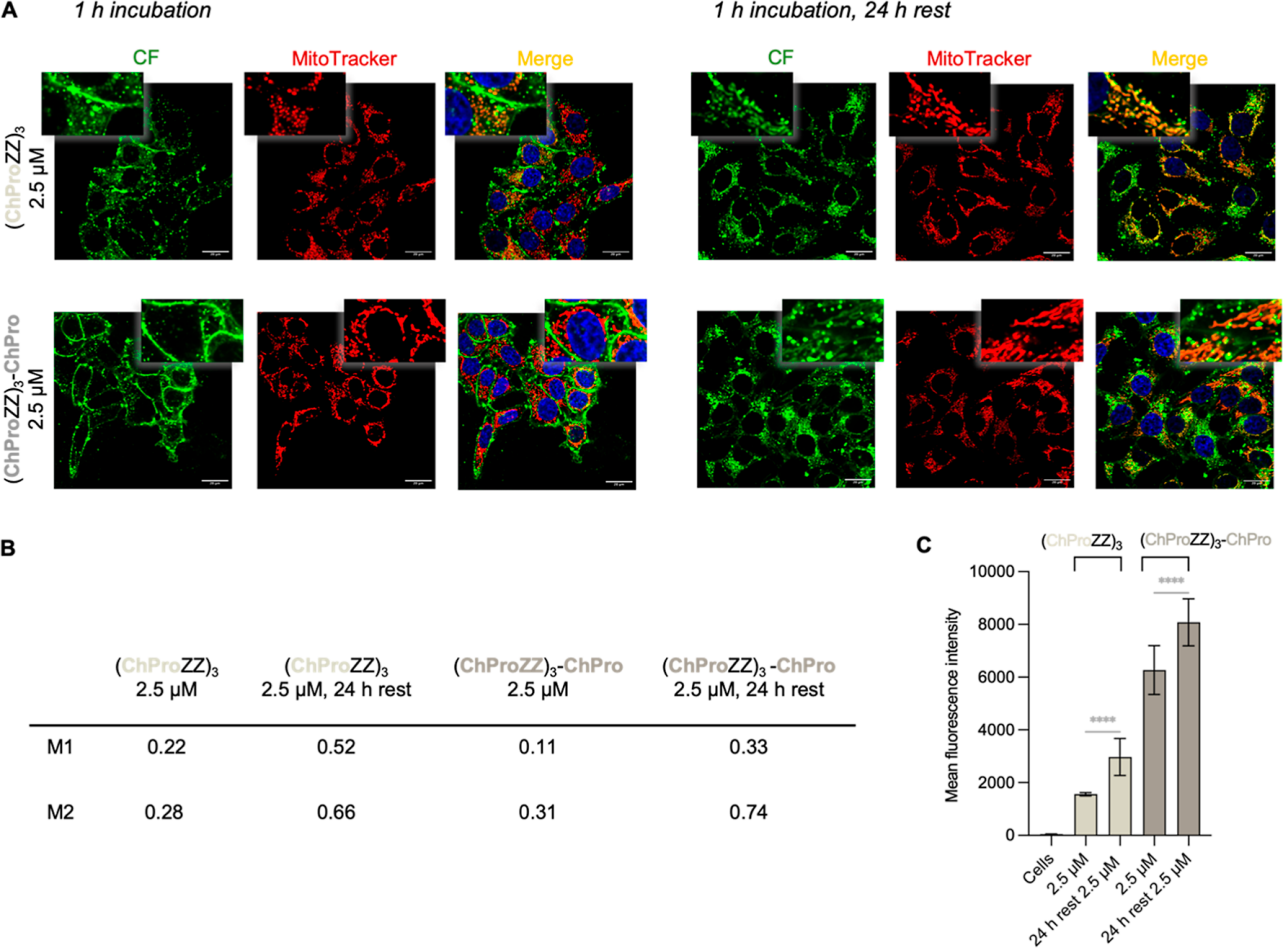
(A) Representative confocal microscopic images of live MCF-7 cells, incubated for 1 h at 37 °C with peptides (ChProZZ)_3_ and (ChProZZ)_3_-ChPro (green) at 2.5 μM in DMEM + 1% FBS (left) and after a 24 h rest in DMEM + 10% FBS (right). Mitochondria were stained with DeepRed MitoTracker (red), merged images (right, orange/yellow indicating colocalization) with Hoechst33352 stain (blue) for the nucleus. (B) Manders’ coefficients: M1 corresponds to the fraction of peptide colocalizing with MitoTracker, M2 corresponds to the fraction of MitoTracker colocalizing with the peptide (C) FACS analysis of the peptides after 1 h incubation at 37 °C at 2.5 μM in DMEM + 1% FBS, and after a 24 h rest in DMEM + 1% FBS. The indicated *P*-values were determined using one-way ANOVA followed by Tukey’s multiple comparisons test per group of peptides (<0.0001­(****)).

During these additional 24 h, both peptides redistributed intracellularly: **(ChProZZ)**_**3**_ transitioned from the cell membrane to mitochondria, with little endosomal entrapment as indicated by limited cytosolic punctate fluorescence (Figure 6A, top row right). **(ChProZZ)**_**3**_ **-ChPro**, which initially localized almost exclusively in or at the plasma membrane, translocated to a large extent to the mitochondria (Figure 6A, bottom row right). Thus, over time, mitochondrial targeting increased significantly. This finding is supported by Manders’ coefficients, indicators for the colocalization of the peptides with MitoTracker, which increased by more than 2-fold (Figure 6B).

To determine whether the peptides migrated to the mitochondria from the cell membrane or were expelled from the cells, we quantified the total cellular fluorescence by FACS after a 1 h incubation time and the subsequent 24 h rest in DMEM supplemented with 10% FBS. These experiments revealed higher fluorescence levels after the 24 h rest period (Figure 6C). This, at first glance, counter intuitive result, corroborates the gradual translocation of the peptides from the membrane to the mitochondria and also indicates gradual escape of the peptides from initially formed endosomes.^51^ The latter conclusion is consistent with the pH-dependent emission of carboxyfluorescein,^52^ which is lower in the acidic environment of endosomes compared to the slightly basic (pH ∼ 8) environment of the mitochondria.

Applying this procedure to the peptides that internalized showed, for most peptides, higher mitochondrial colocalization over time, accompanied by reduced membrane association and endosomal entrapment (Figure S7). With **(ChaZZ)_3_** the least hydrophobic in the series, less fluorescence was observed after 24 h, likely due to clearance from the cytosol and the nucleus. Significantly less fluorescence was observed after 24 h with **(ChaZZ)_2_-Cha**. These findings let us to evaluate the integrity of the peptides, in MCF-7 cell lysate (Figure S8). These studies revealed that all ChPro-containing peptides are stable in cell lysate of MCF-7 over 24 h, implying that the peptide remains intact inside cells. However, **(ChaZZ)_2_-Cha**, the peptide that was not retained in mitochondria and with which less fluorescence after 24 h was observed, degraded in the MCF-7 cell lysate over time. The longer Cha containing peptides, **(ChaZZ)**_**3**_ and **(ChaZZ)_3_-Cha**, degraded only slowly and remained in mitochondria overtime. These data imply that longer translocation times can be beneficial for mitochondrial localization, particularly for hydrophobic peptides.

## Conclusions

In this study, we showed how the installation and arrangement of hydrophobic cyclohexyl moieties within rigid cationic oligoproline-based CPPs influence cellular uptake and subcellular distribution. The systematic variation of hydrophobic groups and their position, peptide length, and flexibility provided short amphipathic CPPs for targeting mitochondria. Our studies show the value of a rigid PPII helical scaffold with cationic guanidinium groups aligned at two edges and hydrophobic cyclohexyl groups at the third edge of the helix for cellular uptake and mitochondria targeting, even at low micromolar concentration. Fine-tuning of the hydrophobicity, for example, by introducing a single cyclohexyl-containing residue at the C-terminus or implementing tertiary instead of secondary amide groups, significantly enhanced both uptake and mitochondrial localization. As such, the work provided design principles for the optimization of mitochondria targeting CPPs. The study also showed that endosomal entrapment and plasma membrane association increase with the hydrophobicity of the CPP. Notably, over time, the amphipathic CPPs redistributed intracellularly from the plasma membrane and endosomes into the mitochondria. Thus, time is a critical parameter for investigating the intracellular localization of, particularly, amphipathic CPPs. Overall, the findings highlight the value of a rigid backbone with proper positioning and balance of cationic and hydrophobic groups, as well as time for effective mitochondria targeting. We, therefore, anticipate that our study will be valuable for targeted intracellular delivery.

## Methods

### Peptide Synthesis, Labeling and Purification

#### Amino Acid Couplings

The peptides were synthesized manually by SPPS using Rink Amide resin (0.67 mmol/g). For the coupling of secondary amines, Fmoc–Xaa–OH (3.0 equiv) and OxymaPure (3.0 equiv) were dissolved in a minimum of DMF/CH_2_Cl_2_ (1:1). DIC (6.0 equiv) was added, and the resulting solution was added to the amino-functionalized resin. The suspension was shaken for 2 h and washed with DMF (3x). For the coupling of primary amines, Fmoc–Xaa–OH (3.0 equiv) and HATU (3.0 equiv) were dissolved in a minimum of DMF. Hünig’s base (6.0 equiv) was added, and the resulting solution was added to the amino-functionalized resin. The suspension was shaken for 60 min and washed with DMF (3x).

#### Fmoc-Deprotection

The Fmoc group was removed by addition of a solution of 40% (v/v) piperidine in DMF to the resin followed by shaking for 5 min, and another 10 min in a freshly added solution of 40% (v/v) piperidine in DMF, followed by extensive washing with DMF.

#### Labeling with Carboxyfluorescein

5­(6)-Carboxyfluorescein (1.5 equiv), Pfp–OH (1.5 equiv) and EDC·HCl (1.5 equiv) were dissolved in a minimum of dry DMF and shaken for 30 min. The resulting solution and Hünig’s base (6.0 equiv) were added to the resin-bound peptide bearing an N-terminal amine. The mixture was shaken for 4 h at rt in the dark and the resin was then thoroughly washed with DMF and CH_2_Cl_2_.

#### Removal of Side-chain Protecting Groups

The peptides were deprotected and cleaved from the resin by addition of a solution of TFA/TIS/H_2_O (95:2.5:2.5), twice for 1 h. Both filtrates were collected and concentrated under a N_2_ flow. Cold Et_2_O was added, and the resulting suspension was centrifuged for 4 min at 1.9 rcf (repeated 2 more times). The peptides were then purified by RP-HPLC. The peptides were used as TFA salts.

The concentration of the peptide stock solutions was determined by UV–vis, measuring the absorbance at 494 nm in PBS (pH 7.4), using a molar extinction coefficient of 65′000 M^–1^ cm^–1^.

### Circular Dichroism (CD) Spectroscopy

The secondary structure of the peptides was analyzed by recording CD spectra of 50 μM solutions in H_2_O (pH 5.7) at 25 °C.

### Cellular Studies

#### Confocal Microscopy

MCF-7 cells were seeded in a 8-μwell Ibidi plate at 25′000 cells/well in DMEM (100 μL, 10% FCS) and allowed to adhere overnight. The medium was removed, the cells were washed with PBS (200 μL, 1x) and incubated with the peptide solution at 20 μM, 10 μM, 5 μM or 2.5 μM in DMEM (200 μL, 1% FCS) for 1 h at 37 °C. The medium was removed, and the cells were washed with PBS (200 μL, 2x). MitoTracker Deep Red was distributed at 20 nM in DMEM (200 μL, 1% FBS) and the cells were incubated at 37 °C for 25 min. The medium was removed, and the cells were washed with PBS (200 μL, 2x). Hoechst333342 was distributed at 2 μM in DMEM (200 μL, 1% FBS) and the cells were incubated at 37 °C for 5 min. The cells were washed with PBS (200 μL, 2x), and FluoroBrite medium (200 μL) was added. The live cells were monitored on the confocal microscope at 37 °C, 5% CO_2_.

For the cells subjected to a 24 h rest period, the FluoroBrite medium was exchanged for DMEM (200 μL, 10% FBS) after the initial imaging, and the cells were kept in this medium for 24 h. The culture medium was then exchanged for FluoroBrite DMEM, and the cells were imaged again.

#### Flow Cytometry

MCF-7 cells were seeded in a 24-well plate at 150′000 cells/well in DMEM (1 mL, 10% FCS), and allowed to adhere overnight. The medium was removed, the cells were washed with PBS (250 μL, 1x) and incubated with the peptide solution at 20 μM, 10 μM, 5 μM or 2.5 μM in medium (200 μL, 1% FCS) for 1 h at 37 °C. The medium was removed, and the cells were washed with PBS (250 μL, 2x). Trypsin (100 μL, 0.05%) was added, and the cells were incubated at 37 °C for 5 min. D-PBS with Mg^2+^ and Ca^2+^ (300 μL, 4 °C) was added, the cells were resuspended and centrifuged for 5 min at 0.4 rcf. The supernatant was carefully removed, and the cells were resuspended in a solution of PBS containing 1.5 μM PI and 2 mM EDTA (400 μL at 4 °C). The cells were added to FACS tubes and kept on ice prior to analysis. Each sample contained 10′000 cells and was analyzed in triplicate. Each experiment was repeated three times.

For the experiments that included the 24 h rest period, the peptides were incubated as described above. After the 1 h incubation time, the cells were washed with PBS (250 μL, 2x), DMEM (250 μL, 10% FBS) was added, and the cells were kept at 37 °C, 5% CO_2_ for 24 h. For a direct comparison of the effect of a 24 h rest period, another set of peptides was incubated with the cells for 1 h. Then, cells of both sets of experiments (1 h followed by the 24 h rest period versus 1 h incubation) were washed with PBS, treated with trypsin, and prepared for FACS analysis as described above. Each sample was run in triplicate and was repeated three times.

## Supplementary Material



## Notes

General aspects and materials, details on experimental procedures, compound characterization, additional data on the structure of the peptides as well as cellular uptake, toxicity and stability (PDF)
