# Supplementary material for: Amphipathic Proline-Rich Cell Penetrating Peptides for Targeting Mitochondria
Source: ACS Chem Biol. 2025 Aug 19;20(9):2298–307. doi: 10.1021/acschembio.5c00479 (PMC12455572; doi:10.1021/acschembio.5c00479)
Supplement: Supplementary file 1 [file cb5c00479_si_001.pdf]

Supporting Information

Amphipathic Proline-rich Cell Penetrating Peptides for Targeting Mitochondria

Adeline Schmitt, Helma Wennemers*

Laboratory of Organic Chemistry, ETH Zürich, D-CHAB, Vladimir-Prelog-Weg 3, 8093 Zürich, Switzerland

Table Of Contents

GENERAL ASPECTS AND MATERIALS **3**

Chemicals and synthesis 3
Reversed Phase High-Performance Liquid Chromatography (RP-HPLC) 3
High Resolution Mass Spectrometry (HR-MS) 4
Circular Dichroism Spectroscopy (CD) 4
UV-Vis Spectroscopy 4
Cell culture 4
Fluorescence-Activated Cell Sorting (FACS) analyzer 4
Confocal microscope 5
Plate reader used for cytotoxicity studies 5

EXPERIMENTAL METHODS **5**

Confocal imaging 5
MTT assay 5
Cell lysate preparation 6
Stability of the peptides in cell lysate 6

PEPTIDE $(ChaRR)_3$ – CONFORMATIONAL AND CELL PENETRATION STUDIES **7**

STUDY OF THE PPII HELICAL STRUCTURE OF THE PEPTIDES **8**

CELL STUDIES **9**

Confocal microscopy of MCF-7 cells after incubation with the peptides at different concentrations 9
Confocal images after staining with LysoTracker 17
Cytotoxicity of the peptides determined by MTT assays 21
Cell analysis after a 24 h resting period 21
Stability of the peptides in MCF-7 cell lysate 28
Flow cytometry at 4 °C 32

ANALYTICAL DATA **35**

Peptides 35
Fmoc-(4S)Gup$(Boc)_2$-OH 50

REFERENCES **52**

General aspects and materials

Chemicals and synthesis

Reagents were purchased in the highest commercially available grade and were used as received. DMF, CH_3CN, Et_2O, triisopropylsilane (TIPS), trifluoroacetic acid (TFA), *N,N'*-Diisopropylcarbodiimide (DIC), Oxyma Pure, 5(6)-Carboxyfluorescein, *N*-(3-Dimethylaminopropyl)-*N'*-ethylcarbodiimide hydrochloride (EDC.HCl), Ethylenediaminetetraacetic acid (EDTA) and propidium iodide (PI) were purchased from Sigma-Aldrich/Merck. Dichloromethane (CH_2Cl_2), piperidine, N,N-Diisopropylethylamine (DIPEA), were purchased from Acros/Thermo Fisher Scientific. Fmoc-Hyp(*t*Bu) and Fmoc-L-Cha were purchased from abcr. Fmoc-(4*S*)-cyclohexyl-L-proline ((2*S*,4*S*)-Fmoc-ChPro-OH) was purchased from Iris Biotech. Pentafluorophenol (Pfp-OH) was purchased from FluoroChem. HATU was purchased from Bachem.
Rink Amide-Polystyrene AM resin (200-400 mesh, 0.67 mmol/g) was purchased from Novabiochem.
Trypsin-EDTA (0.05%/0.25%) in Ca^{2+}- and Mg^{2+}-deficient phosphate-buffered saline (PBS) (1X), PBS (pH 7.4), Dulbecco's PBS (DPBS) (pH 7.4), GlutaMAX™ and Fetal calf serum (FCS) were purchased from Gibco. 3-(4,5-Dimethylthiazol-2-yl)-2,5-Diphenyltetrazolium Bromide (MTT) was purchased from Thermo Scientific. Hoechst 33342, LysoTracker™ Deep Red, MitoTracker™ Deep Red and FluoroBrite DMEM were purchased from Invitrogen.

Plates for microscopy were purchased from Ibidi. 5 mL round bottom polystyrene Test tubes for FACS analysis were purchased from Falcon. Cell culture plates (24-well plate) and cell culture flasks were purchased from TPP.

H_2O refers to "Milli-Q" water with a resistivity of 18.2 MΩ·cm obtained via an Arium611VF purification system from Sartorius.

Amino acid couplings were carried out on a peptide shaker, and completion of the amino acid coupling was verified using a Microflex from Bruker with Matrix Assisted Laser Desorption Ionisation (MALDI) and a Time Of Flight (TOF) mass analyzer. In case of incomplete coupling, the peptide was subjected to a second coupling under the same conditions.

Reactions were stirred magnetically and monitored by thin layer chromatography using silica gel aluminum plates (Merck, 0.2 mm, 60 F254). Compounds were detected using standard visualizing agents (UV fluorescence, 254 nm, and ninhydrin stain). Flash chromatography was performed on silica gel (Merck, Kieselgel 60 F254 230-400 mesh). ^{1}H- and ^{13}C-NMR spectra were recorded on 400 MHz or 500 MHz instruments.

Reversed Phase High-Performance Liquid Chromatography (RP-HPLC)

For analytical and preparative RP-HPLC, HPLC-grade solvents were used.

Preparative RP-HPLC was performed on an HPG-3200BX UltiMate 3000 system from Dionex using as mobile phase, solvent A: H_2O containing 1% v/v CH3CN and 0.1% v/v TFA and solvent B CH_3CN. A ReproSil Gold C18 column (250 x 10 mm, 10 µm particle size, 120 Å pore size) from Dr. Maisch was used as stationary phase. A flow rate of 6 mL/min was used the peptides were purified at room temperature.

Analytical RP-HPLC was performed on a LPG3400SD UltiMate 3000 system from Dionex, using as mobile phase solvent B: H_2O containing 1% v/v CH_3CN and 0.1% v/v TFA, and solvent A: CH_3CN. A Reprosil Gold C4 column (150 x 4.0 mm, 5 µm particle size, 120 Å pore size) was used as stationary phase. A flow rate of 1 mL/min and an oven temperature of 25 °C were set. The purity was determined using the UV signal at 214 nm.
Two sets of conditions were used:
Conditions A were used to compare the hydrophobicity of the peptides and to assess the purity of most peptides: gradient of 70 % to 45 % B during 20 min.
Conditions B were used to assess the purity of the more polar peptides: gradient of 80 % to 55 % B during 20 min.

High Resolution Mass Spectrometry (HR-MS)

HR-MS was performed using a Daltonics maXis from Bruker equipped with an electrospray ionization source and a Qq-TOF mass analyzer. The instrument was operated by the Molecular and Biomolecular Analysis (MoBiAs) service at D-CHAB, ETH Zürich.

Circular Dichroism Spectroscopy (CD)

CD spectroscopic analyses were performed on a Chirascan Plus instrument from Applied Biophysics connected to a NitroPack nitrogen generator from Parker Balston and a TC125 temperature controller from Quantum Northwest. Samples were measured in quartz cuvettes from Hellma Analytics with a path length of 1.0 mm. CD spectra were acquired at a spectral bandwidth of 1 nm with a time constant of 5 s and a step resolution of 1 nm. All the peptides were recorded at 25 °C in H_2O at 50 µM.

UV-Vis Spectroscopy

Spectra were recorded on an Agilent Cary 300 spectrometer from 800 nm to 200 nm, with a step resolution of 1 nm. The samples were measured using a quartz cuvette from Hellma Analytics with a path length of 10.0 mm at room temperature.

Protein concentration of cell lysates was determined using a NanoPhotometer N60 from Implen.

Cell culture

MCF-7 cells were obtained from the American Type Culture Collection (ATCC).
The cells were grown in a humidified 5% CO_2 atmosphere at 37 °C Dulbecco's Modified Eagle's Medium (DMEM), high glucose, GlutaMAX™ (4 mM) supplemented with L-glutamine (4 mM), gentamicin (10 mg/L), and 10% fetal calf serum (FCS) superior (standardized).

Fluorescence-Activated Cell Sorting (FACS) analyzer

On a BD LSRFortessa flow cytometer, CF was excited at 488 nm and monitored with 530/30 bandpass, PI was excited at 561 nm and monitored with a 610/20 bandpass. Mean fluorescence values were determined from the histograms using FlowJo 10.0.6 software while dead cells were removed according to PI staining. Below is an example, CF-Ahx-$(ChaZZ)_3$, of gating, set on untreated cells and propagated to all samples. Each sample was run in triplicate, and each experiment was repeated at least three times.

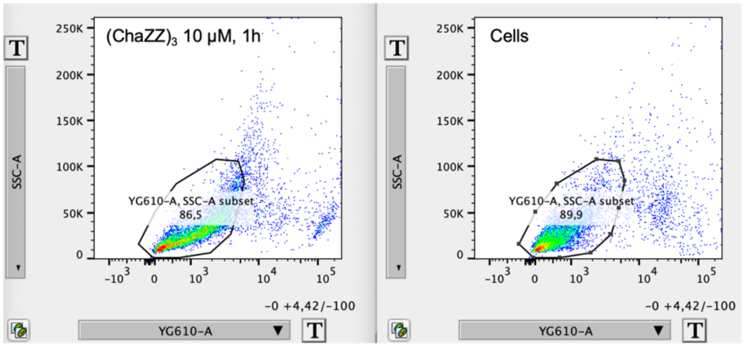

Confocal microscope

Fluorescence images of cells were collected using a Nikon Eclipse T1 (inverse) microscope equipped with a Yokogawa Confocal Scanner Unit CSU-W1-T2, containing a spinning disk (50 µm). The microscope is equipped with two EMCCD Andor iXon Ultra cameras. Emission in the blue channel (405 nm) was filtered with a 450/50 bandpass filter, emission in the green channel (488 nm) was filtered with a 525/50 bandpass, and the emission in the red channel (641 nm) was filtered with a 708/75 bandpass filter. Fluorescence images were obtained using an oil-immersion objective with a magnification of 100 × 1.4 CFI Plan Apo Oil. The microscope was operated using VisiView ("Metamorph") software. Each experiment was repeated at least three times. The images acquired were adjusted using ImageJ2 software. Correlation coefficients were determined on entire images using JaCOP plugin.[1]

Plate reader used for cytotoxicity studies

The toxicity of the peptides was assessed by an MTT assay (see below). The absorbance of the formazan solution produced by cells was recorded on a Spark 10M plate reader from Tecan, and the data was analyzed with the software SPARKCONTROL.
Absorption was measured at 570 nm at 25 °C, and cell viability was calculated relative to PBS treated control cells, which were set to 100 % viability according to the following formula:
% cell viability = $(A_{570} – A_{570,\ Background})/ (A_{570,\ Control} – A_{570,\ Background}) \times 100$

Experimental methods

Confocal imaging

MCF-7 cells were seeded in a 8-µwell Ibidi plate at 25'000 cells/well in DMEM (100 µL, 10% FCS) and allowed to adhere overnight. The medium was removed. The cells were washed with PBS (200 µL, 1x) and incubated with the peptide solution at 20 µM, 10 µM, 5 µM, or 2.5 µM in DMEM (200 µL, 1% FCS) for 1 h at 37 °C. The medium was removed, and the cells were washed with PBS (200 µL, 2x).
For imaging of mitochondria, MitoTracker Deep Red was added as described in the manuscript. For imaging of endosomes/lysosomes, LysoTracker Deep Red was added (200 µL, 20 nM in DMEM, 1% FBS) followed by incubation of the cells at 37 °C for 25 min. The medium was removed, and the cells were washed with PBS (200 µL, 2x). For imaging of the nucleus, Hoechst333342 was added at (200 µL, 2 µM in DMEM, 1% FBS) followed by incubation of the cells at 37 °C for 5 min. The cells were washed with PBS (200 µL, 2x), and FluoroBrite medium (200 µL) was added. The live cells were monitored on the confocal microscope at 37 °C, 5% CO_2.

MTT assay

MCF-7 cells were seeded in a 96-well plate at 7'500 cells/well in cell culture medium (200 µL, 10% FCS), and allowed to adhere overnight. The medium was removed, the cells were washed with PBS (100 µL, 1x) and incubated with the peptide solution at 20 µM, 10 µM, 5 µM, or 2.5 µM in DMEM (200 µL, 1% FCS) for 1 h at 37 °C. The medium was removed, and the cells were washed with PBS (100 µL, 1x). The MTT solution in DMEM (110 µL, 1.2 mM, 1% FBS) was added. The cells were incubated for 3 h at 37 °C. The supernatant was carefully removed, and DMSO (100 µL) was added. The plate was gently shaken for 30 min and the absorption of formazan solution at 570 nm was measured on the plate reader.
To assess the cytotoxicity of the peptides after the 24 h rest period, the peptides were incubated as stated above. The cells were then washed with PBS (100 µL, 2x), DMEM (100 µM, 10% FBS) was added, and the cells were incubated at 37 °C for 24 h. The medium was then removed, and the procedure was resumed as described above.

Cell lysate preparation

MCF-7 cells were grown to confluency in a T-175 flask. Cells were washed with PBS (12 mL, 4 °C) and subsequently scraped off into PBS (7 mL, 4 °C) and collected in a Falcon tube. The suspension was then sonicated for 3 sec at an amplitude of 30% followed by a 30 sec rest period, and the procedure was repeated three time. The insoluble fraction was removed by centrifugation for 10 min at 12.000 rcf, at 4 °C and the supernatant was transferred to a new tube. UV-Vis spectroscopy (see above) determined a protein concentration of the obtained cell lysate of 3 mg/mL.

Stability of the peptides in cell lysate

The peptides were added (final concentration of 200 µM) to the MCF-7 cell lysate, with a protein concentration adjusted to 1 mg/mL with PBS. Immediately after addition of the peptide to the cell lysate, a sample (80 µL) was taken as the t = 0 min time point. The sample was diluted in MeCN (1:1), kept on ice for 5 min and centrifuged (12.000 rcf, 10 min, 4 °C). The supernatant was filtered and analyzed by analytical RP-HPLC (at 488 nm) using the standard conditions (gradient 70 % to 45 % of B over 20 min at 25 °C). The stability of the peptides was monitored at time points 0 min, 1 h and 24 h.

Peptide (ChaRR)$_3$ – conformational and cell penetration studies

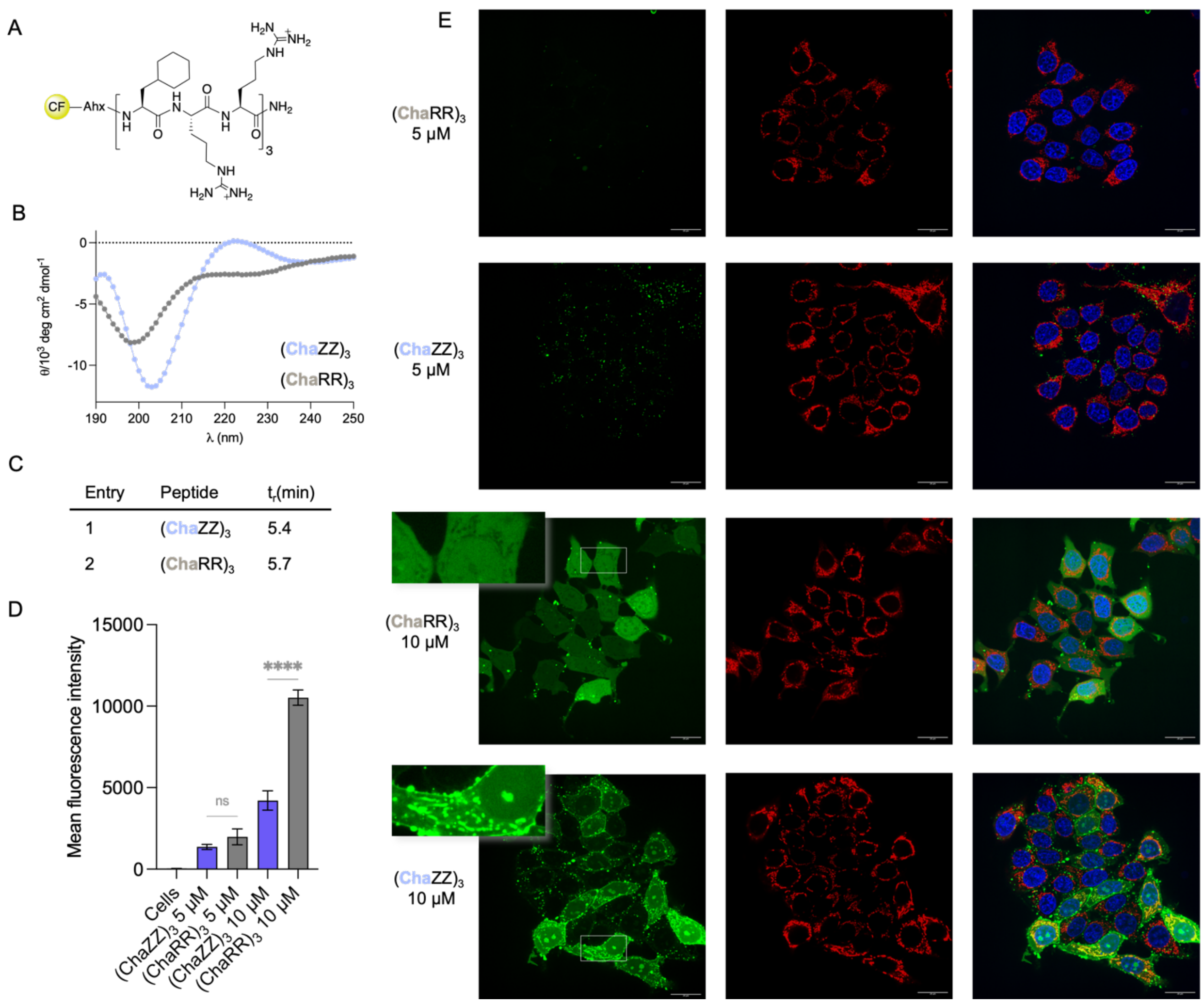

Figure S1. Comparison of (ChaRR)$_3$ and (ChaZZ)$_3$ peptide. A) Structure of the CF-Ahx-(ChaRR)$_3$ peptide; CF = 5(6)-Carboxyfluorescein, Ahx = aminohexanoic acid. B) CD spectrum of the peptide at 50 µM in H_2O, indicative of a random coil conformation (minimum at 200 nm and maximum at 215 nm), in contrast to PPII helical (ChaZZ)$_3$ (minimum at 203 nm and maximum at 223 nm). C) Retention time of the peptides on RP-HPLC (C4 column, gradient 15 to 35 % B) indicates similar hydrophobicity for the two peptides. D) FACS analysis revealed enhanced cellular uptake of (ChaRR)$_3$ compared to (ChaZZ)$_3$. E) Confocal images at different concentrations (the peptide, in green, left, MitoTracker, in red, middle, merge image, orange/yellow indicating colocalization, right). Both peptides show low uptake at 5 µM. At 10 µM, (ChaRR)$_3$ is excluded from mitochondria, while (ChaZZ)$_3$ localizes in the mitochondria. The indicated P-values were determined using one-way ANOVA followed by Tukey's multiple comparisons test per group of peptides (0.1234 (ns), 0.03328(*), 0.0021 (**), 0.0002 (***), <0.0001(****)).

Study of the PPII helical structure of the peptides

To compare the PPII helicity of the peptides containing hydrophobic residues in the sequence **CF-Ahx-[X-ZZ]$_3$**, where X is Val, Phe, Cha or Trp, we plotted a Gup oligomers peptide (**Ac-Z$_8$**) on the same graph (Figure S2). For clarity, we excluded **(ValZZ)$_3$** from this plot. The zoom of the maxima and the minima show that their location hardly deviates across the different peptides. These observations imply that the non-proline residues are tolerated within the helical structure.
The more intense minima and maxima of the tryptophan containing peptide **(WZZ)$_3$** arise from the absorbance of Trp in the far-UV.[2]

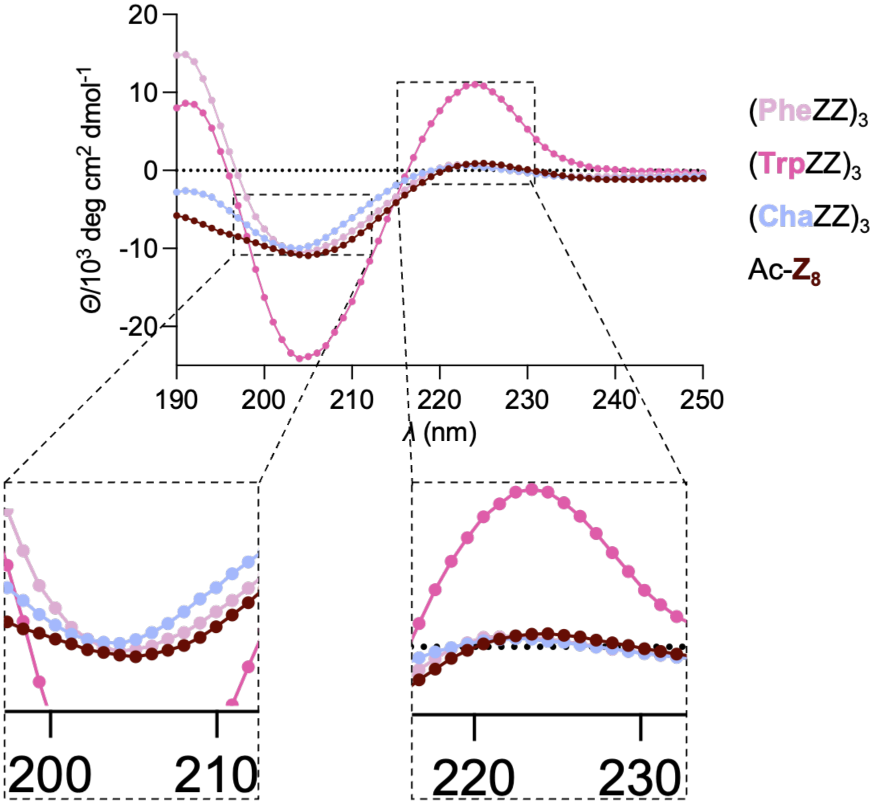

Figure S2. Comparative CD spectrum of the peptides at 50 µM in H_2O, indicating PPII helical peptides (minimum at 203-205 nm and maximum at 223-225 nm)

Cell studies

Confocal microscopy of MCF-7 cells after incubation with the peptides at different concentrations

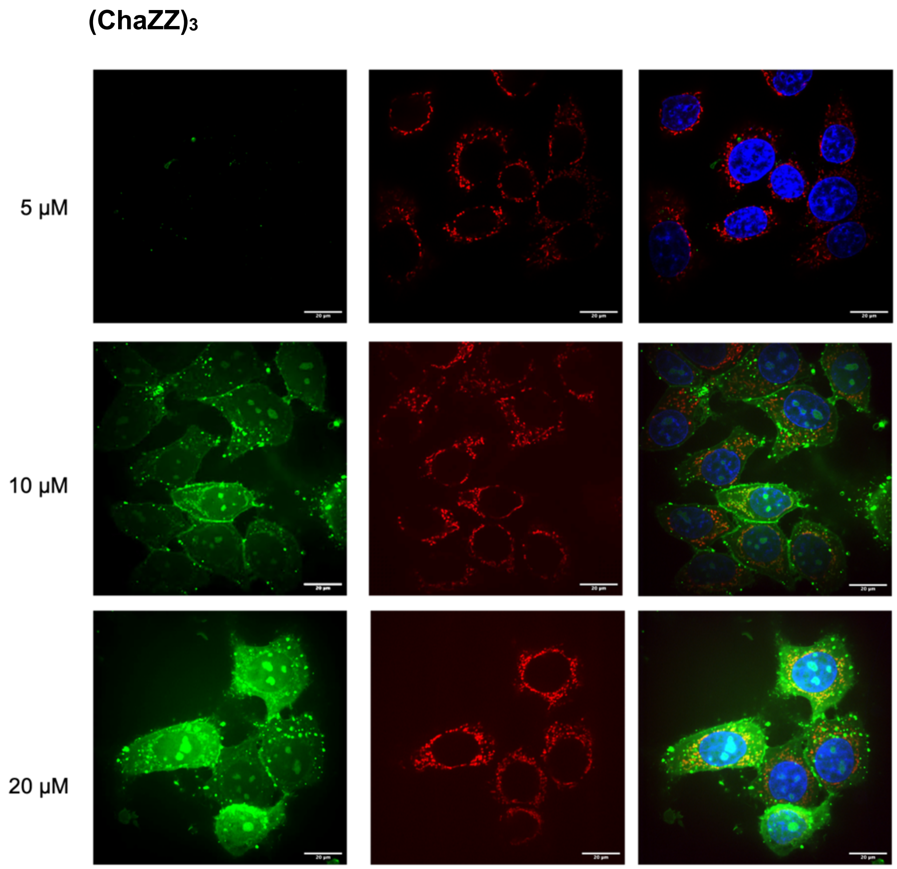

Figure S3. Peptide (in green, left) at different concentrations, with MitoTracker Deep Red (in red, middle) and merge images (yellow/orange indicates colocalization, right)

(ChaZZ)$_3$-Cha

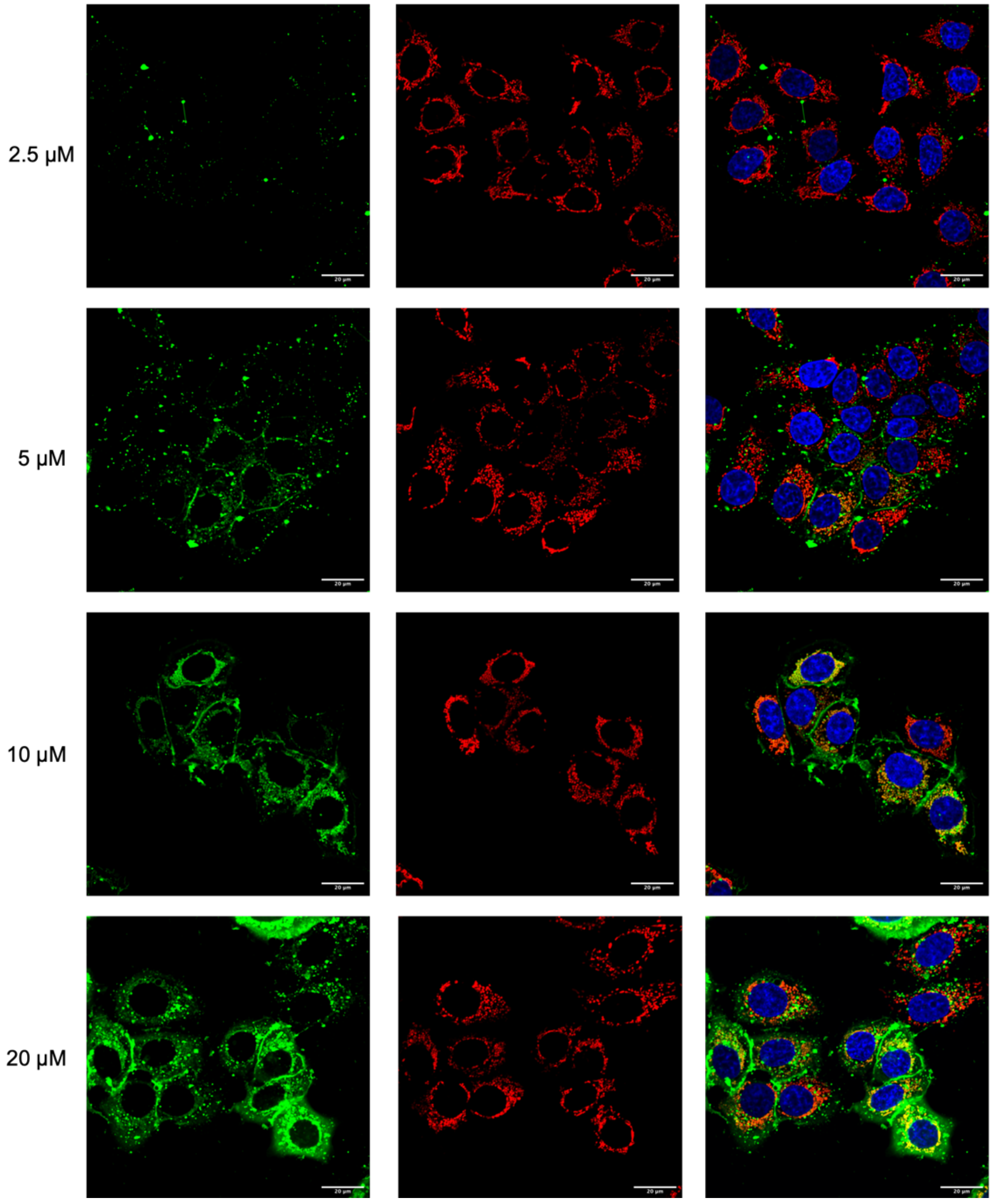

Figure S3. – continued –

$(ChaZZ)_2$

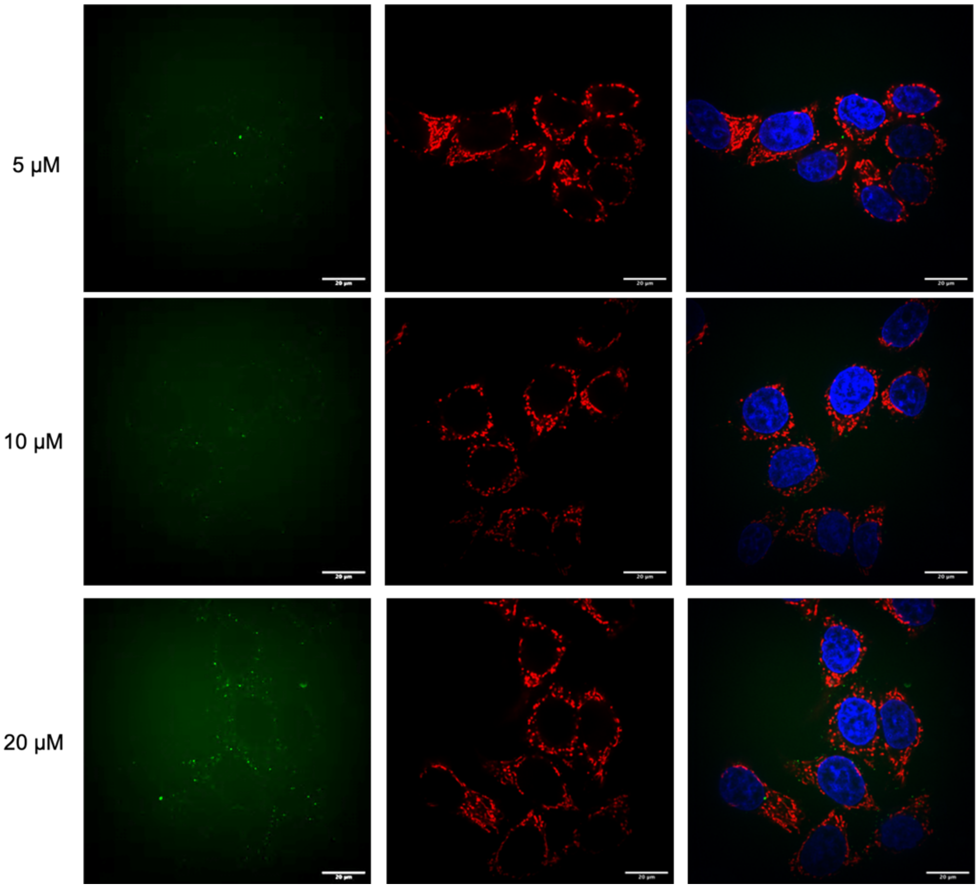

Figure S3. – continued –

(ChaZZ)$_2$-Cha

5 µM

10 µM

20 µM

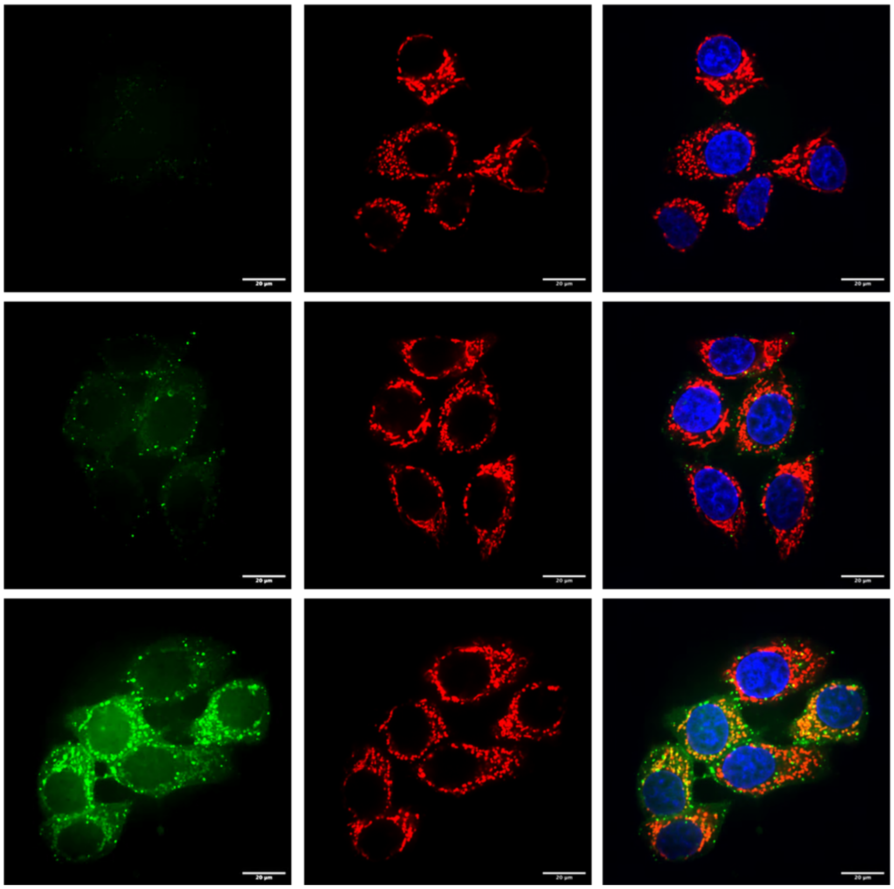

Figure S3. – continued –

$(ChProZZ)_3$

2.5 µM

5 µM

10 µM

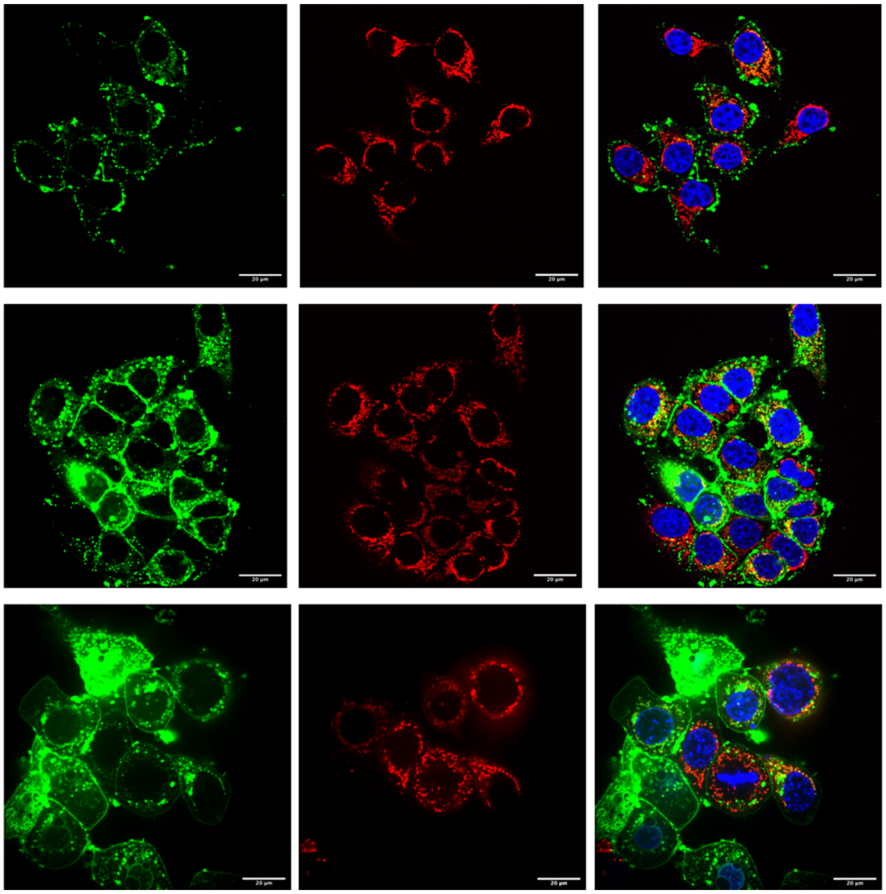

Figure S3. – continued –

(ChProZZ)$_3$-ChPro

2.5 µM

5 µM

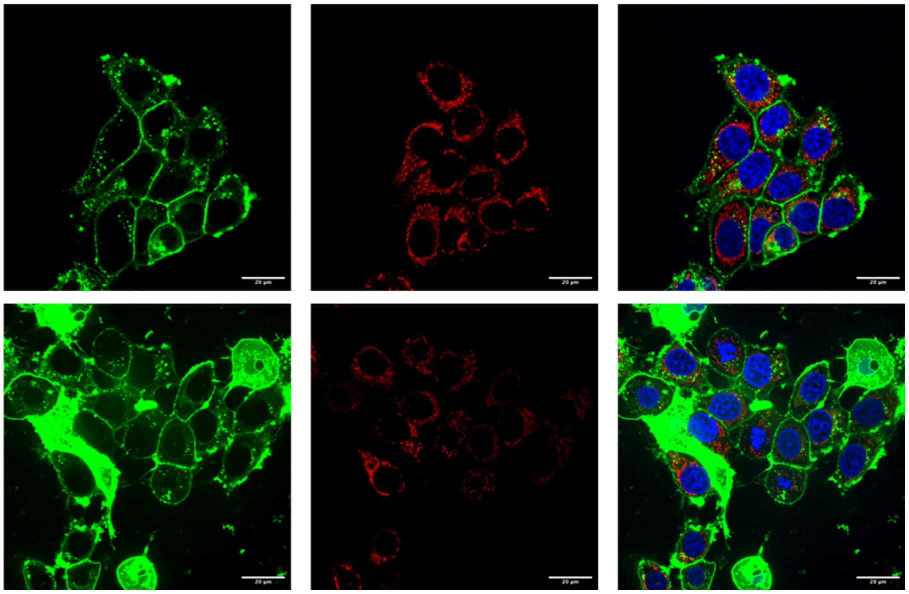

Figure S3*.* – continued –

(ChProZZ)$_2$

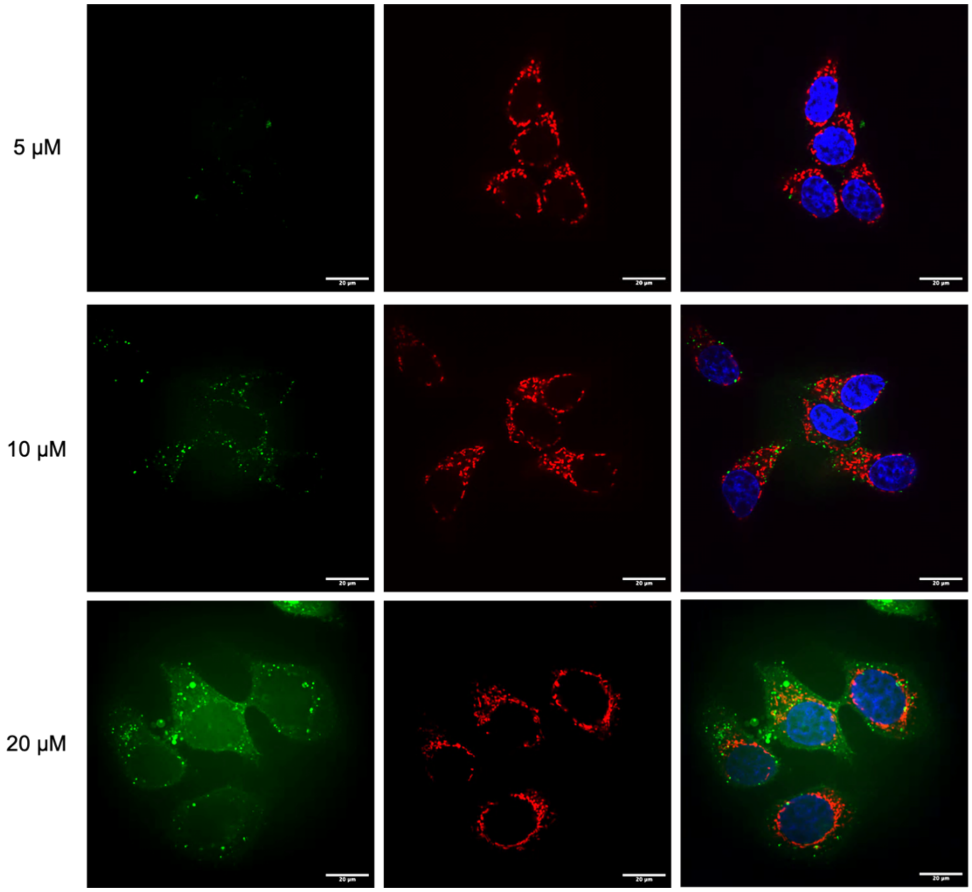

Figure S3. – continued –

$(ChProZZ)_2$-ChPro

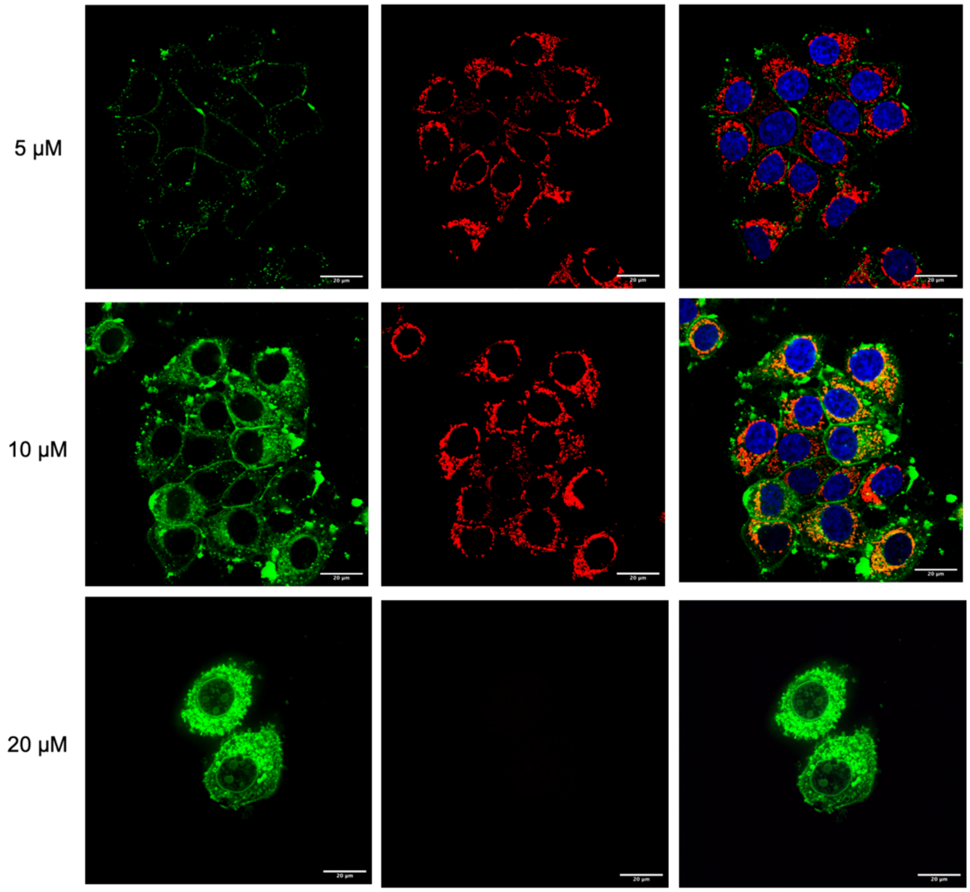

Figure S3. – continued –

Confocal images after staining with LysoTracker

Confocal images at peptide concentrations at which mitochondria targeting was observed. LysoTracker Deep Red as counterstain for endosomes.

(ChaZZ)$_3$

10 µM

20 µM

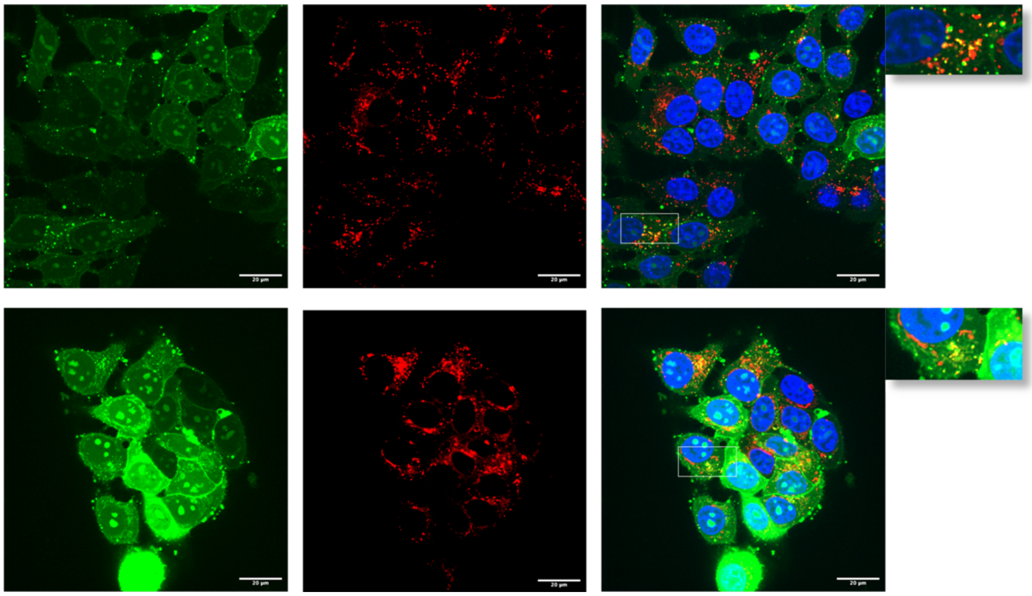

Figure S4. Peptide in green (left), LysoTracker Deep Red in red (middle), merged image with yellow/orange indicating colocalization (right). The square white box indicates the enlarged section.

(ChaZZ)$_3$-Cha

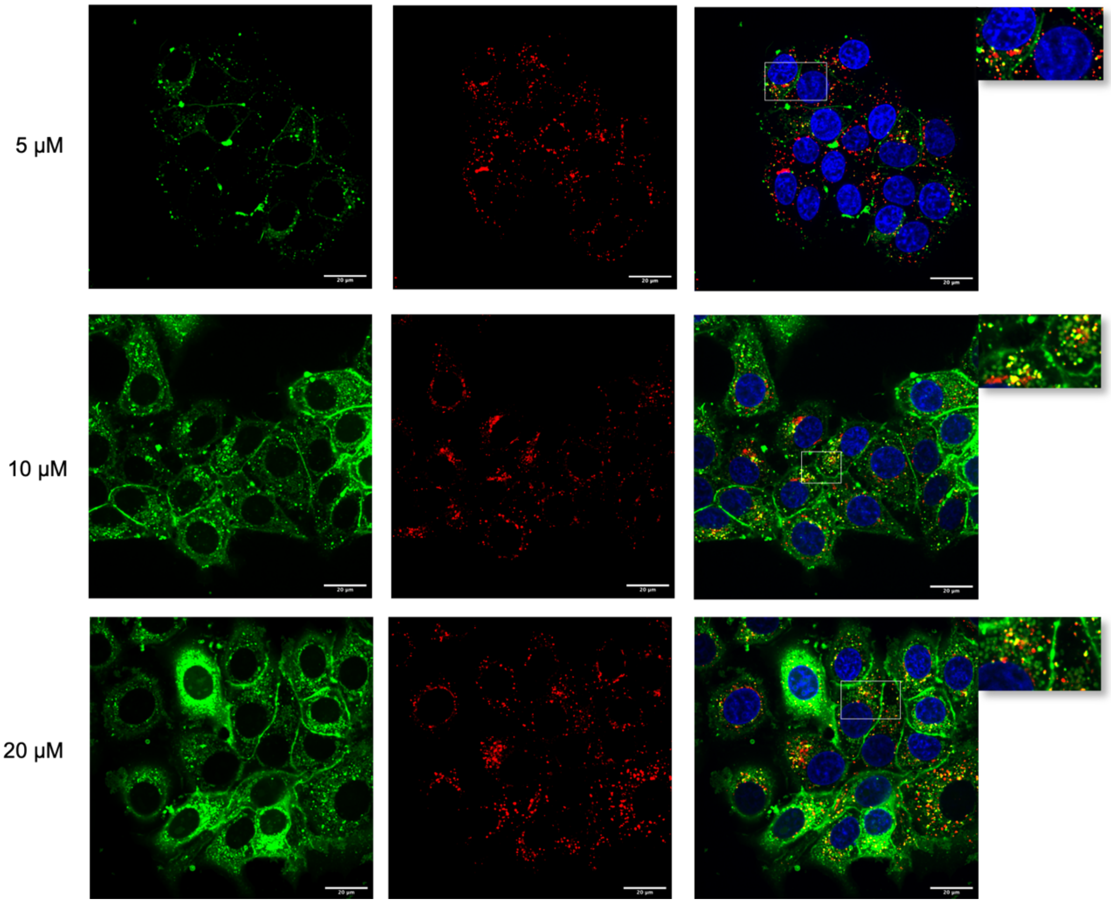

Figure S4. – continued –

(ChProZZ)$_3$

2.5 µM

5 µM

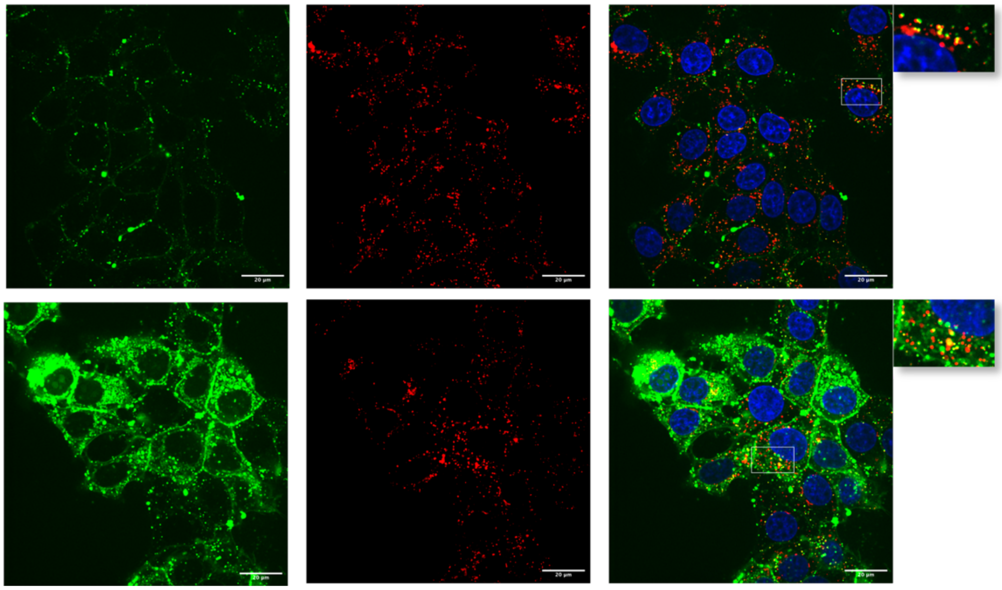

(ChProZZ)$_3$-ChPro

2.5 µM

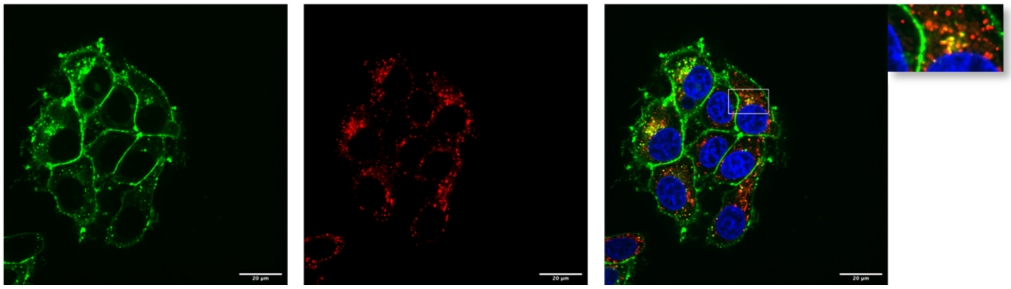

(ChaZZ)$_2$-Cha

20 µM

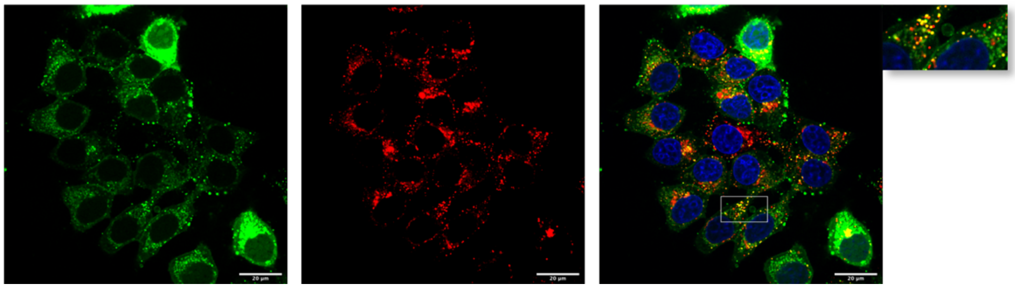

Figure S4. – continued –

(ChProZZ)$_2$

20 µM

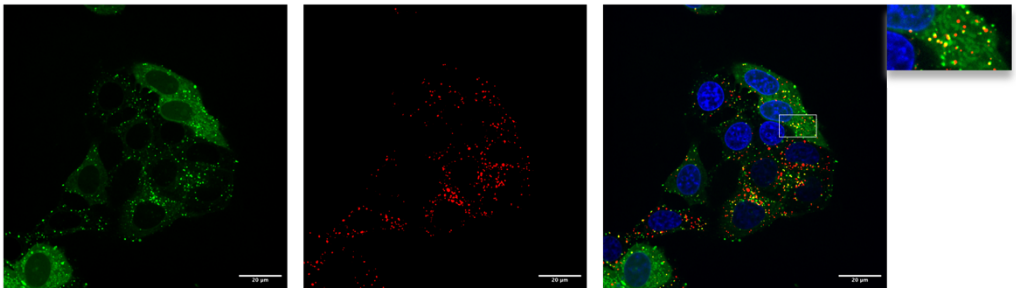

(ChProZZ)$_2$-ChPro

10 µM

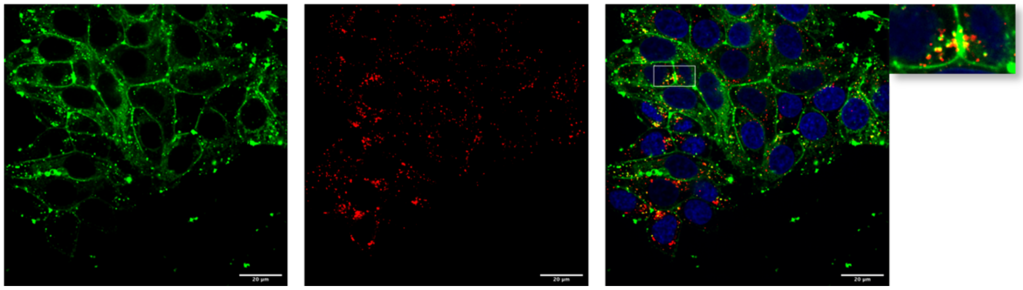

Figure S4. – continued –

Cytotoxicity of the peptides determined by MTT assays

1 h incubation

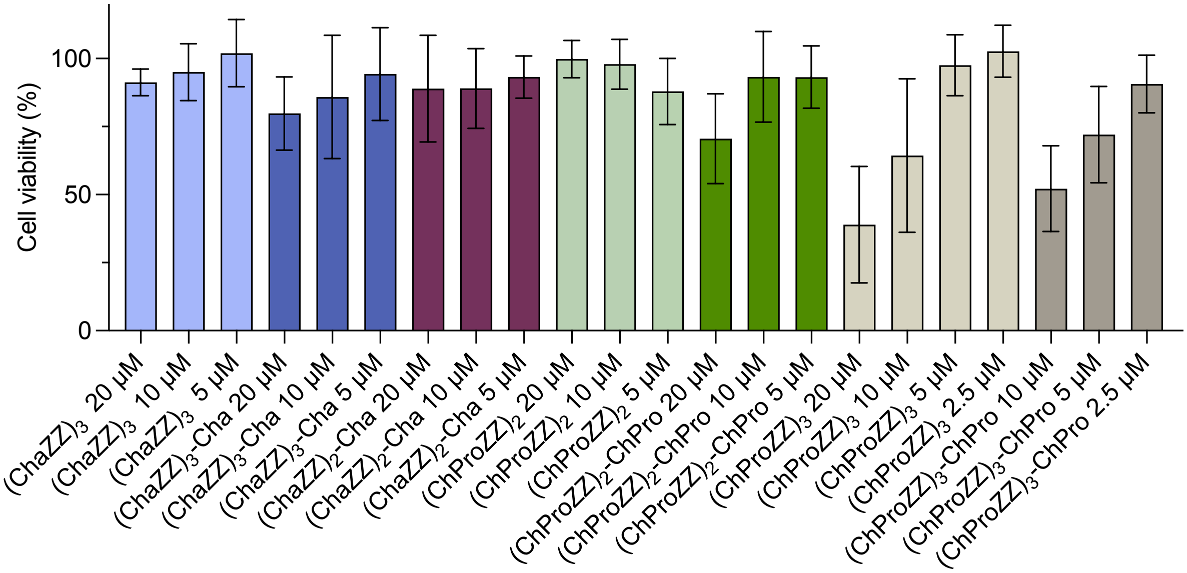

Figure S5. Cytotoxicity of the peptides at different concentrations after 1 h incubation in DMEM + 1 % FBS at 37 °C.

1 h incubation followed by 24 h rest

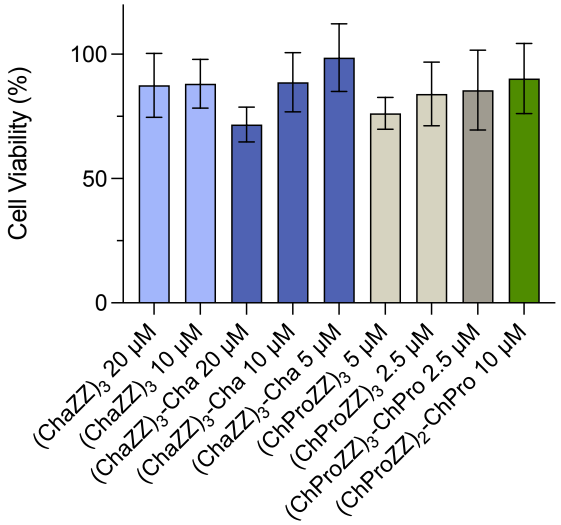

Figure S6. Cytotoxicity of the peptides after 1 h incubation at 37 °C in DMEM + 1% FBS, followed by a 24 h rest period in DMEM + 10 % FBS at 37 °C. Only the peptides exhibiting improved mitochondria targeting were included in this study.

Cell analysis after a 24 h resting period

Comparative analysis of MCF7 cells immediately after 1 h incubation with the respective peptide at 37 °C and after washing and a 24 h rest in DMEM + 10 % FBS. A) Representative confocal microscope images of the respective peptide after 1 h (in green, left block) and after the 24 h rest period (right block). Staining of mitochondria with MitoTracker (in red, middle) and the merged images (orange/yellow corresponds to colocalization, the nucleus is stained in blue). B) Correlation factors, where M1 corresponds to the fraction of the peptide colocalizing with MitoTracker, M2 corresponds to the fraction of MitoTracker colocalizing with the peptide, and PCC to the Pearson's correlation coefficient (PCC). C) Comparative FACS analysis of the peptides at different concentrations.

$(ChaZZ)_3$

A

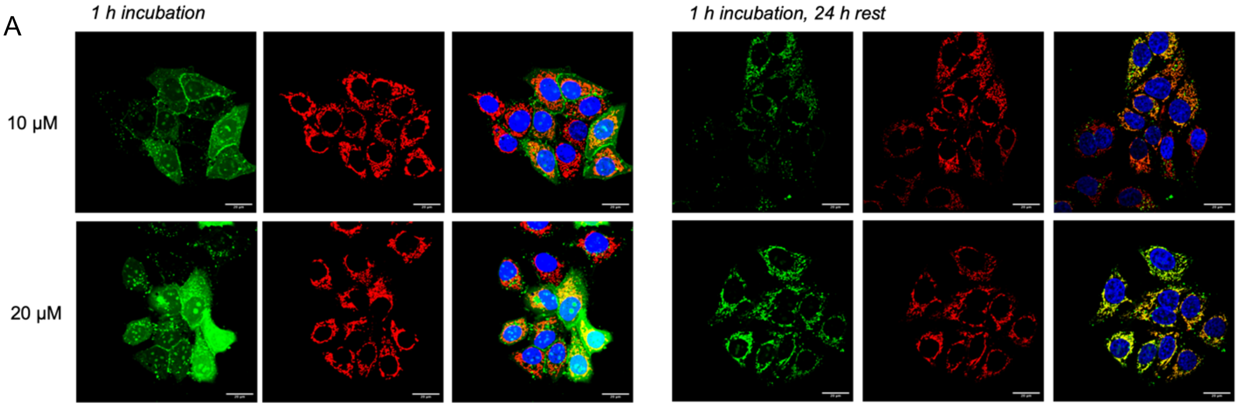

B

$(ChaZZ)_3$	10 µM, 1h	10 µM, 1 h, 24 h rest	20 µM, 1h	20 µM, 1 h, 24 h rest
M1	0.24	0.62	0.23	0.50
M2	0.20	0.57	0.64	0.80
PCC	0.24	0.55	0.23	0.62

C

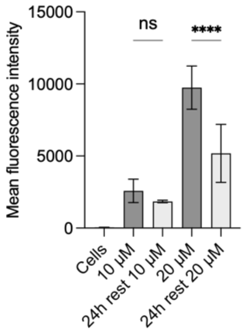

Figure S7.

A) After 1 hour of incubation, $(ChaZZ)_3$ localized in mitochondria, nucleoli, and the cytoplasm (left). Following a 24-hour rest period, the peptide localized predominantly in the mitochondria (right). B) This redistribution is reflected by colocalization analysis with correlation coefficientsmore than a 2-fold greater in peptide overlap with MitoTracker at 10 µM, and a similar improvement in MitoTracker overlap with the peptide at 10 µM. C) FACS analysis revealed lower total cellular fluorescence after the 24 h rest. This finding is consistent with release of the peptide from nucleoli and cytoplasm into the extracellular space.

(ChaZZ)$_3$-Cha

A

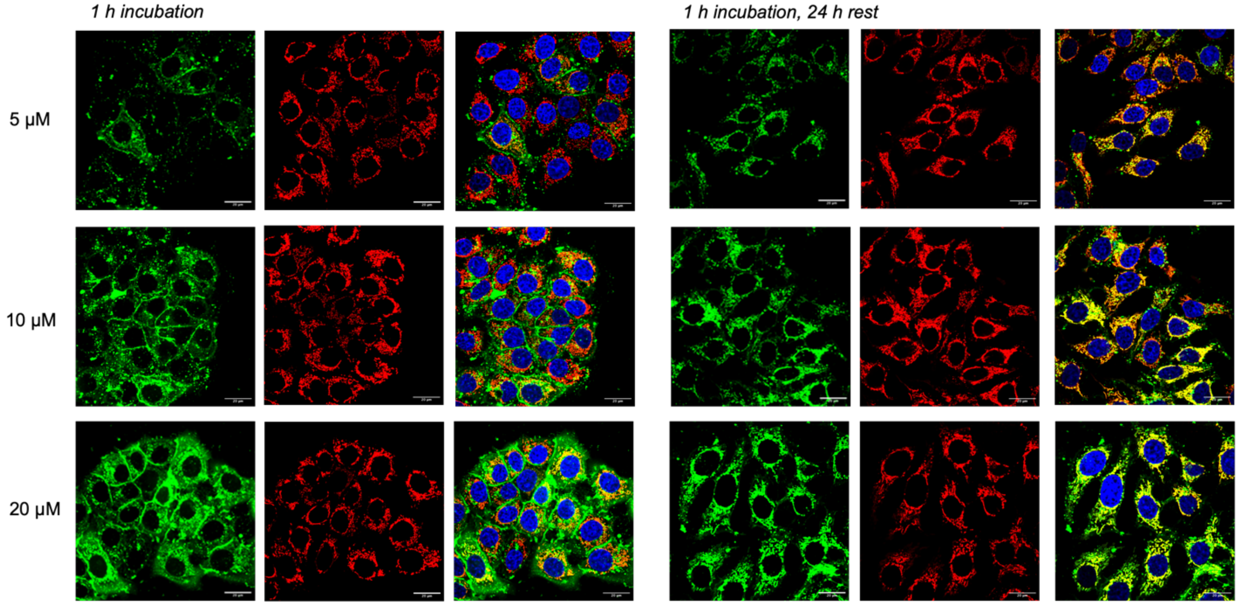

B

(ChaZZ)$_3$-Cha	5 µM, 1h	5 µM, 1 h, 24 h rest	10 µM, 1h	10 µM, 1 h, 24 h rest	20 µM, 1h	20 µM, 1 h, 24 h rest
M1	0.20	0.61	0.30	0.45	0.26	0.46
M2	0.20	0.73	0.53	0.97	0.85	0.85
PCC	0.12	0.56	0.29	0.61	0.39	0.39

C

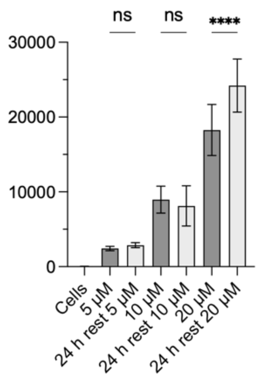

Figure S7. – continued –

A) After 1 hour of incubation, (ChaZZ)$_3$-Cha localized in mitochondria, endosomes, and the plasma membrane (left). Following a 24-hour rest period, the peptide localized predominantly in the mitochondria (right). B) This redistribution is reflected by colocalization analysis with correlation coefficients more than 1.5-fold greater in peptide overlap with MitoTracker (M1), and up to 3.6-fold greater in MitoTracker overlap with the peptide (M2). C). FACS data revealed constant or increased total cellular fluorescence after the 24 h rest. This finding is consistent with redistribution of the peptide from the plasma membrane and endosomes into the mitochondria.

(ChProZZ)$_3$

A

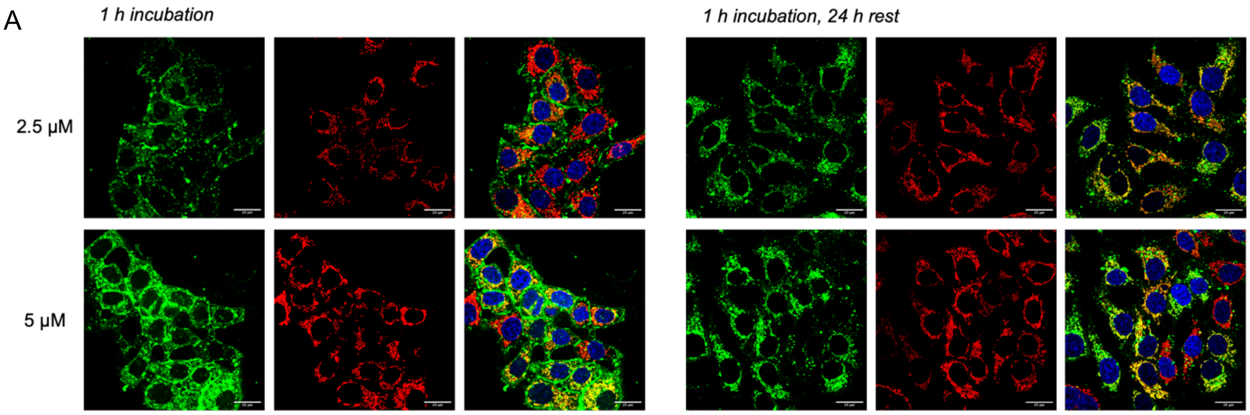

B

(ChProZZ)$_3$	2.5 µM, 1h	2.5 µM, 1 h, 24 h rest	5 µM, 1h	5 µM, 1 h, 24 h rest
M1	0.22	0.52	0.29	0.44
M2	0.28	0.67	0.72	0.89
PCC	0.19	0.52	0.32	0.53

C

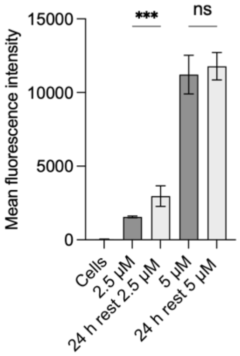

Figure S7. – continued

A) After 1 hour of incubation, (ChProZZ)$_3$ localized in mitochondria, endosomes, and the plasma membrane (left). Following a 24-hour rest period, the peptide localized predominantly in the mitochondria (right). B) This redistribution is reflected by colocalization analysis with correlation coefficients more than 1.5-fold greater in peptide overlap with MitoTracker (M1), and up to 3.4-fold greater in MitoTracker overlap with the peptide (M2). C). FACS data revealed constant or increased total cellular fluorescence after the 24 h rest. This finding is consistent with redistribution of the peptide from the plasma membrane and endosomes into the mitochondria.

(ChProZZ)$_3$-ChPro

A

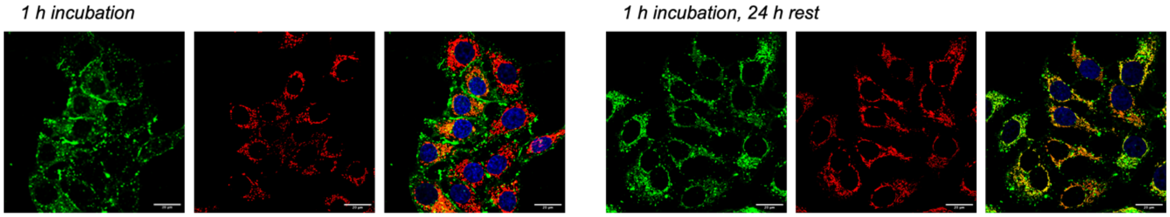

B

(ChProZZ)$_3$-ChPro	2.5 µM, 1h	2.5 µM, 1 h, 24 h rest
M1	0.11	0.33
M2	0.31	0.74
PCC	0.11	0.47

C

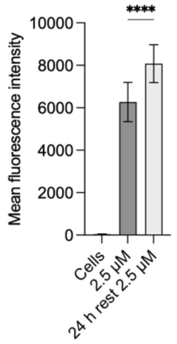

Figure S7. – continued –

A) After 1 hour of incubation, (ChProZZ)$_3$-ChPro localized mainly in endosomes, and the plasma membrane (left). Following a 24-hour rest period, the peptide localized predominantly in the mitochondria (right). B) This redistribution is reflected by colocalization analysis with correlation coefficients of 3-fold greater in peptide overlap with MitoTracker (M1), and of 1.5-fold greater in MitoTracker overlap with the peptide (M2). C). FACS data revealed increased total cellular fluorescence after the 24 h rest. This finding is consistent with redistribution of the peptide from the plasma membrane and endosomes into the mitochondria.

(ChProZZ)$_2$-ChPro

A

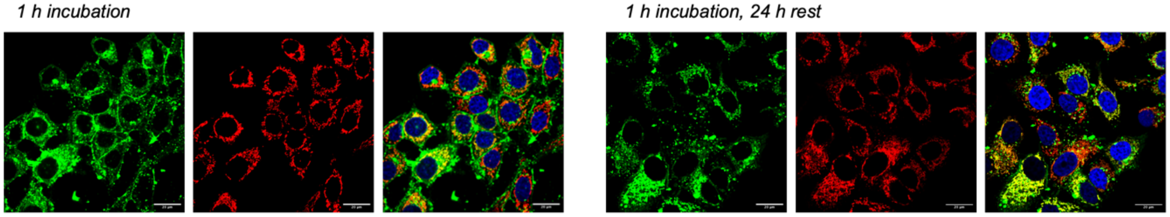

B

(ChProZZ)$_2$-ChPro	10 µM, 1h	10 µM, 1 h, 24 h rest
M1	0.28	0.48
M2	0.68	0.78
PCC	0.33	0.53

C

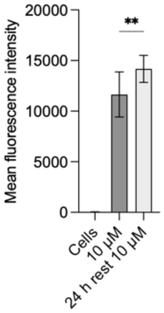

Figure S7. – continued –

A) After 1 hour of incubation, $(ChProZZ)_2$-ChPro localized mainly in mitochondria, endosomes, and the plasma membrane (left). Following a 24-hour rest period, the peptide localized predominantly in the mitochondria (right). B) This redistribution is reflected by colocalization analysis with correlation coefficients of 1.7-fold greater in peptide overlap with MitoTracker (M1), and of 1.2-fold greater in MitoTracker overlap with the peptide (M2). C). FACS data revealed increased total cellular fluorescence after the 24 h rest. This finding is consistent with redistribution of the peptide from the plasma membrane and endosomes into the mitochondria.

$(ChaZZ)_2$-Cha

A

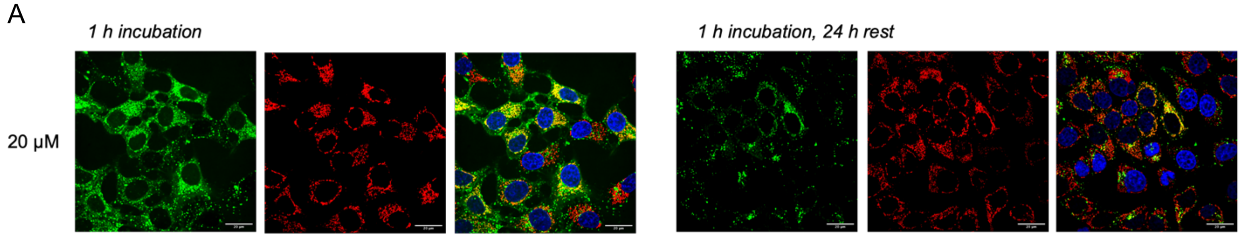

B

$(ChaZZ)_2$-Cha	20 µM, 1h	20 µM, 1 h, 24 h rest
M1	0.44	0.56
M2	0.79	0.29
PCC	0.51	0.39

C

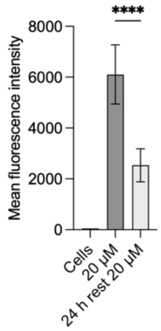

Figure S7. – continued –

A) After 1 hour of incubation, $(ChaZZ)_2$-Cha localized in mitochondria and the cytoplasm (left). Following a 24-hour rest period, the peptide localized in the cytoplasm as punctuated fluorescence and remains slightly in the mitochondria (right). B) This redistribution is reflected by colocalization analysis with correlation coefficients of only 1.3-fold greater in peptide overlap with MitoTracker, and a strong decrease of 0.4-fold in MitoTracker overlap with the peptide. C) FACS analysis revealed lower total cellular fluorescence after the 24 h rest. This finding is consistent with release of the peptide from the cytoplasm and from mitochondria to a certain extent, into the extracellular space.

(ChProZZ)$_2$

A

1 h incubation *1 h incubation, 24 h rest*

20 µM

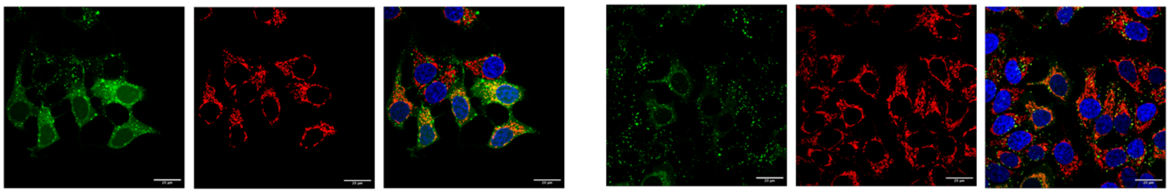

B

(ChProZZ)$_2$	20 µM, 1h	20 µM, 1 h, 24 h rest
M1	0.28	0.38
M2	0.46	0.19
PCC	0.33	0.26

C

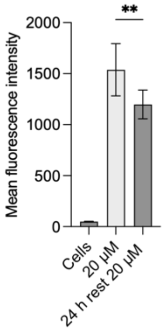

Figure S7. – continued –

A) After 1 hour of incubation, (ChProZZ)$_2$ localized in mitochondria and the cytoplasm (left). Following a 24-hour rest period, the peptide localized in the cytoplasm as punctuated fluorescence and remains slightly in the mitochondria (right). B) This redistribution is reflected by colocalization analysis with correlation coefficients of only 1.3-fold greater in peptide overlap with MitoTracker, and a decrease of 0.4-fold in MitoTracker overlap with the peptide. C) FACS analysis revealed lower total cellular fluorescence after the 24 h rest. This finding is consistent with release of the peptide from the cytoplasm and from mitochondria to a certain extent, into the extracellular space.

Stability of the peptides in MCF-7 cell lysate

(ChProZZ)$_3$ and (ChProZZ)$_3$-ChPro peptides are stable in MCF-7 cell lysate, even after 24 h incubation.

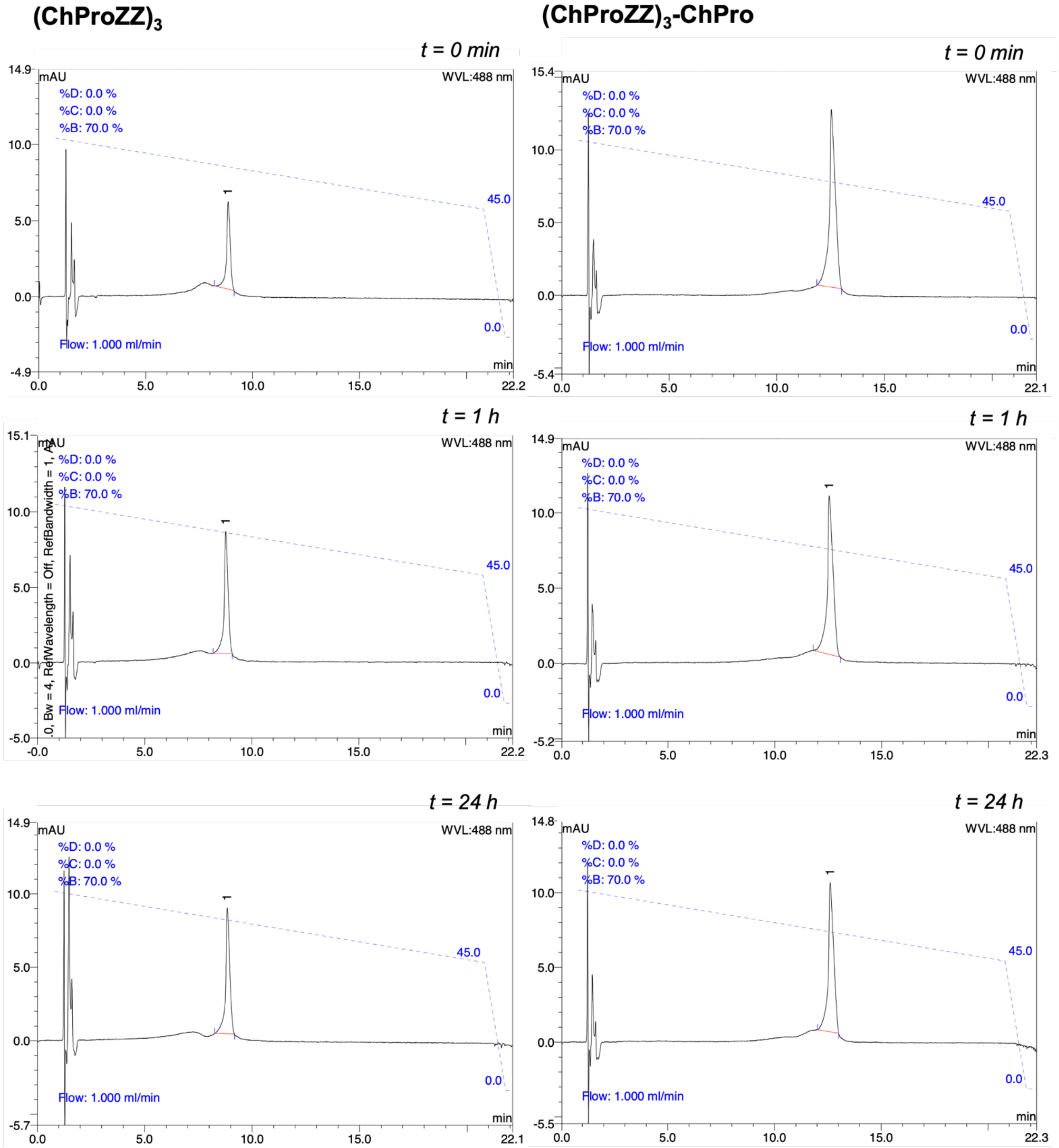

Figure S8. RP-HPLC trace of the respective peptides upon incubation in MCF-7 cell lysate (1 mg/mL) after 0 min (top), 1 h (center) and 24 h (bottom), monitored at 488 nm.

$(ChProZZ)_2$ and $(ChProZZ)_2$-ChPro peptides are stable in MCF-7 cell lysate, even after 24 h incubation.

$(ChProZZ)_2$

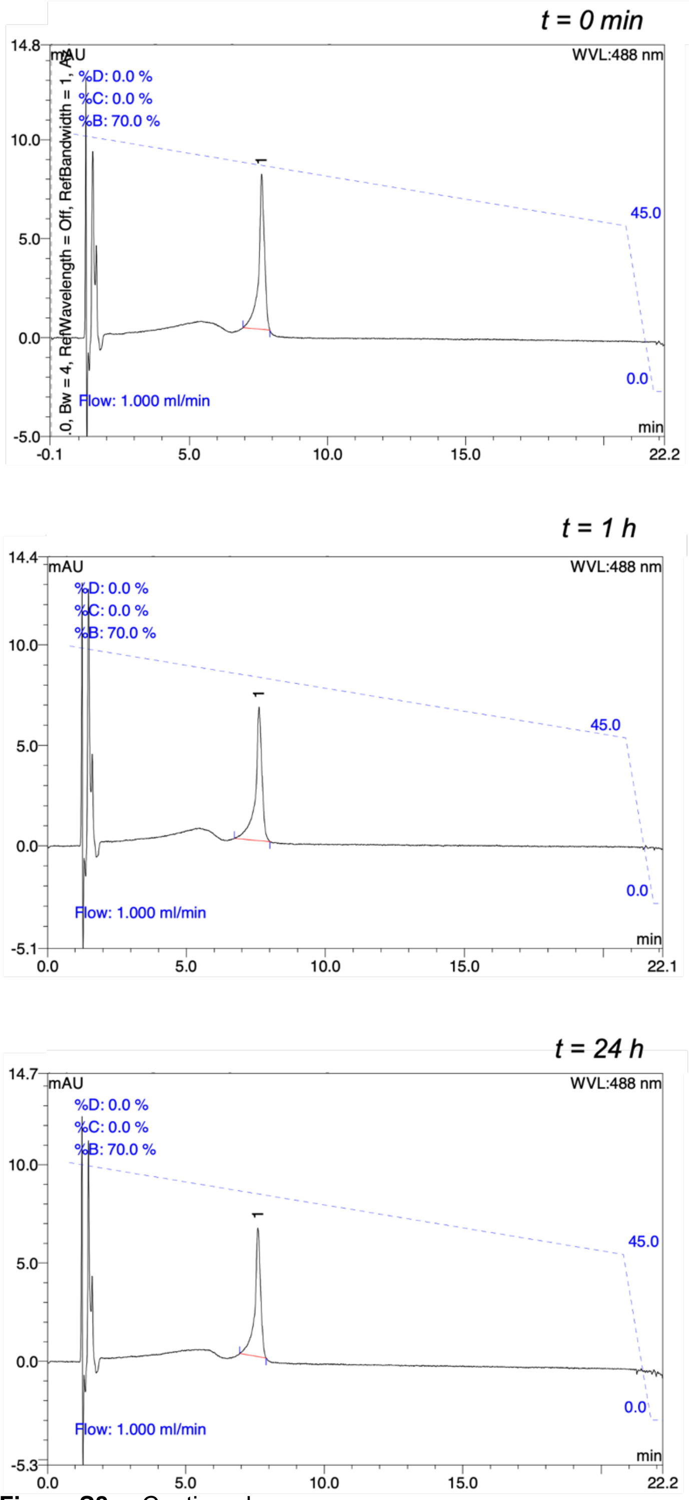

$(ChProZZ)_2$

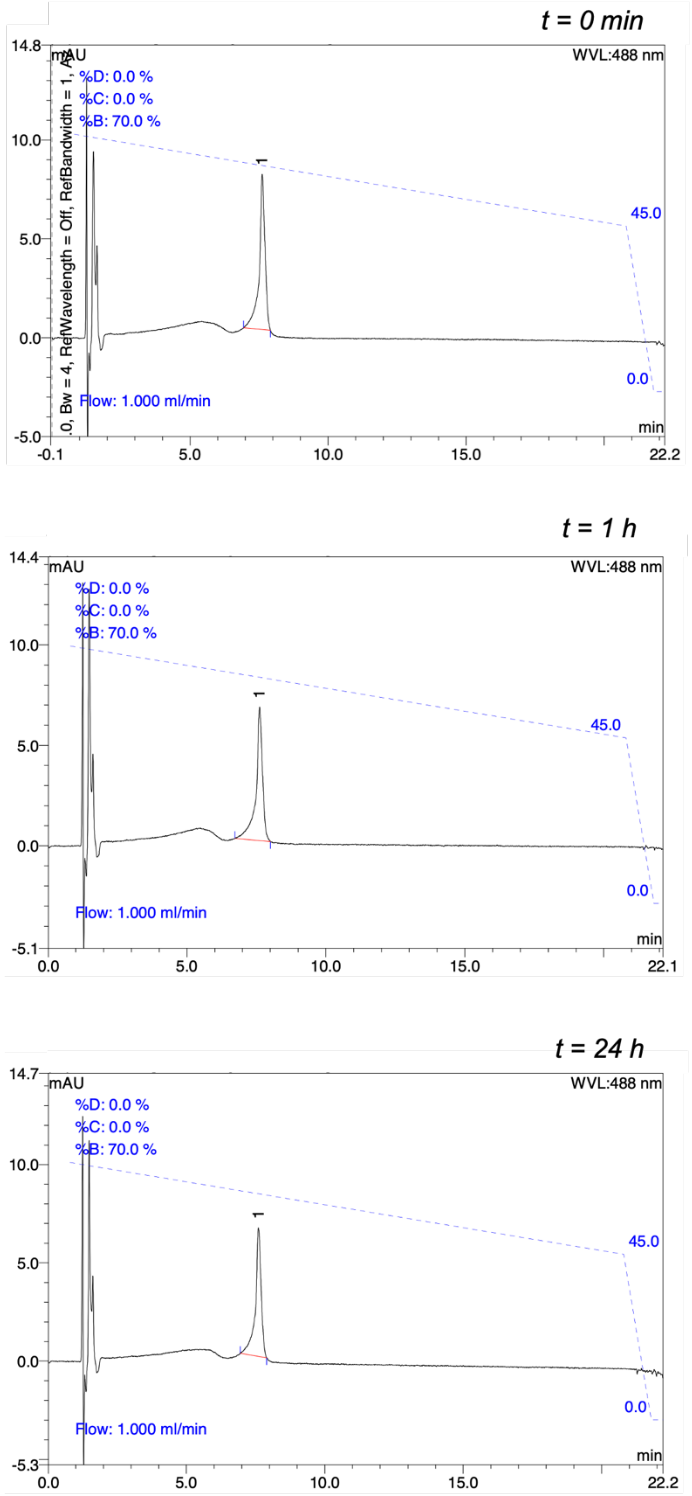

Figure S8. – Continued –

$(ChaZZ)_3$ is stable in MCF-7 cell lysate, even after 24 h incubation. Approximately 6% of $(ChaZZ)_3$-Cha degraded in MCF-7 cells lysate at 37 °C after 24 h incubation.

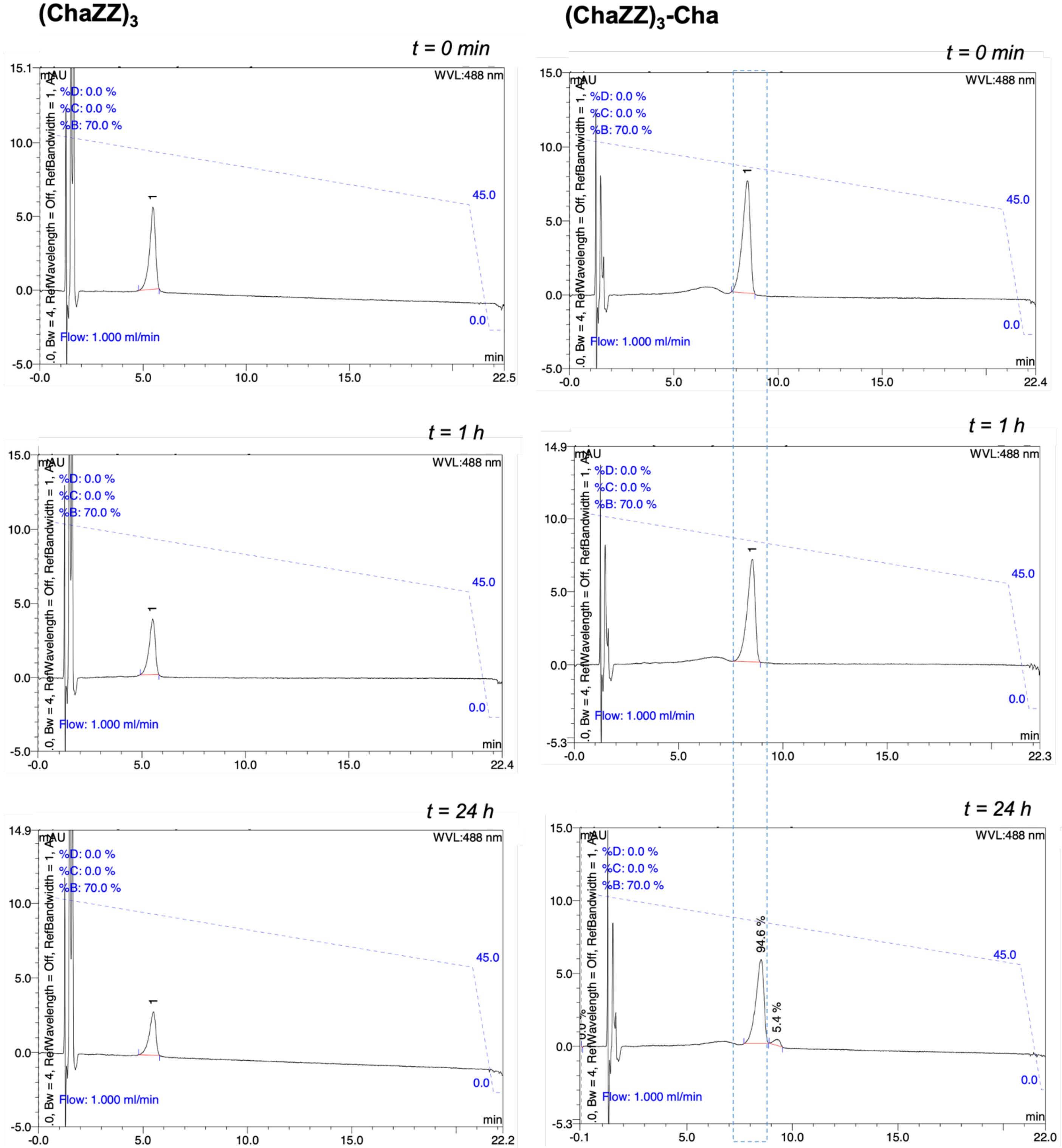

Figure S8. – Continued –

(ChaZZ)$_2$-Cha is is stable in MCF-7 cell lysate after 1 h, but degrades significantly during 24 h.
Figure S8. – Continued –

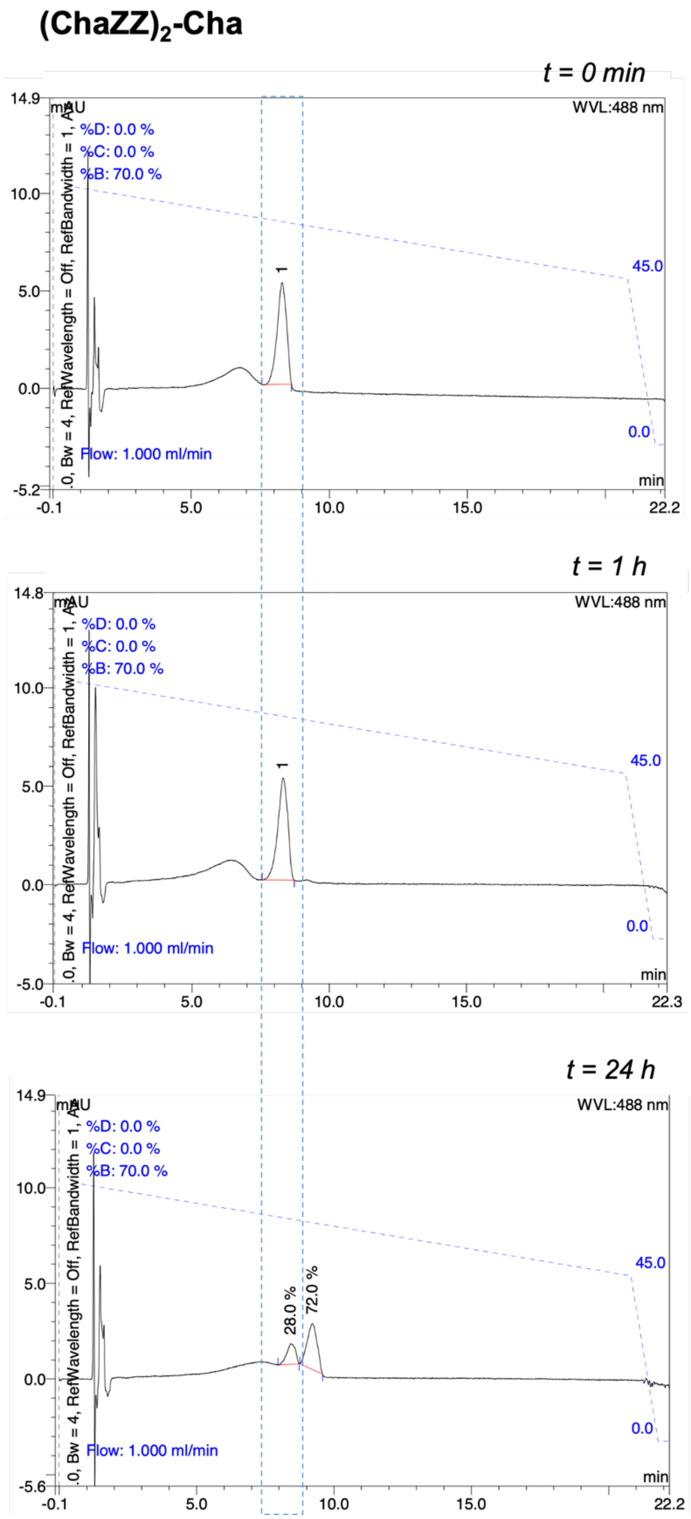

Figure S8. – Continued –

Flow cytometry at 4 °C

$(ChaZZ)_3$ **$(ChaZZ)_3$-Cha**

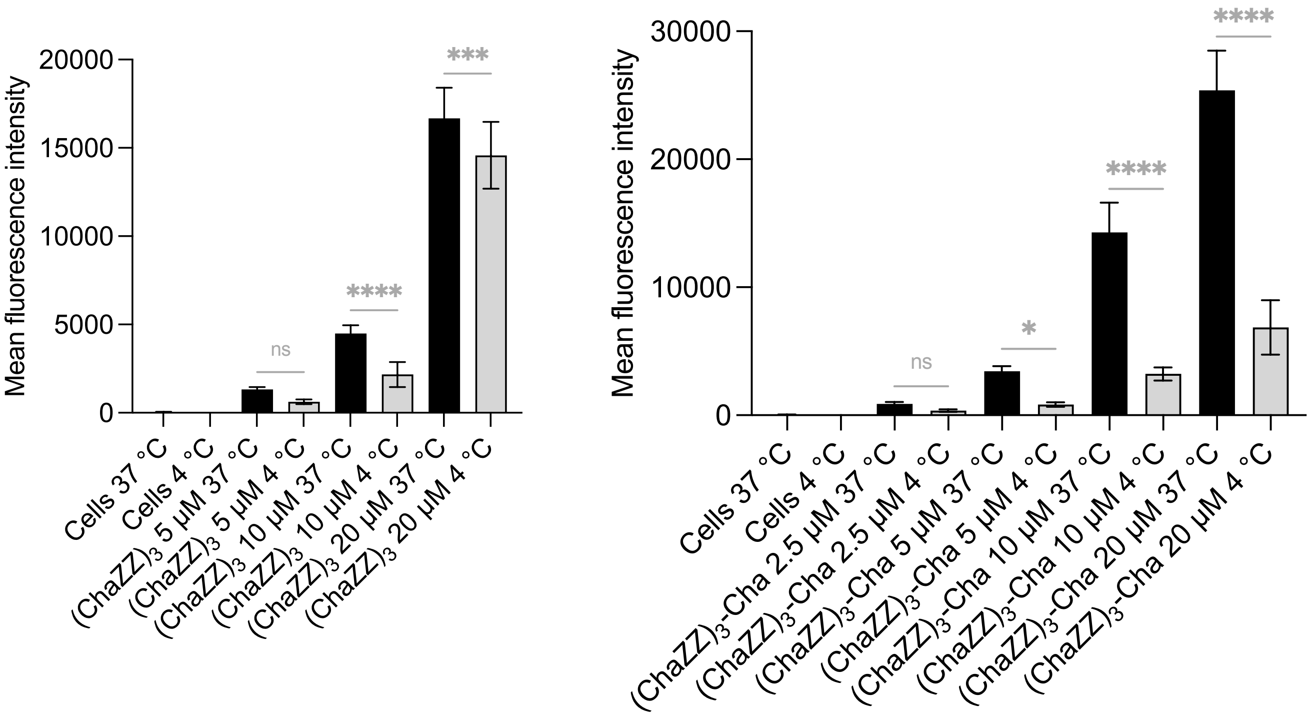

Figure S9. Comparative FACS analysis of the peptides incubated at different concentrations for 1 h at either 37 °C (dark bars) or 4 °C (grey bars). After 1 h incubation at 4 °C, the fluorescence signal is in case of most of the peptides lower, indicating an endocytosis pathway. The close-to-level signal of $(ChaZZ)_3$ at 20 µM could indicate a combination of different entry mechanisms. The indicated P-values were determined using one-way ANOVA followed by Tukey's multiple comparisons test per group of peptides (0.1234 (ns), 0.03328(*), 0.0021 (**), 0.0002 (***), <0.0001(****)).

(ChProZZ)$_3$

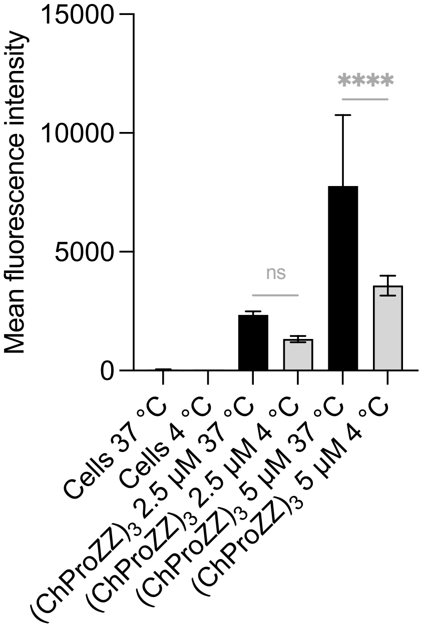

(ChProZZ)$_3$-ChPro

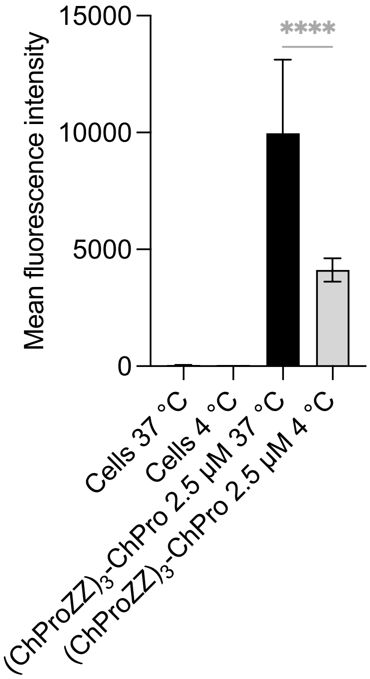

(ChaZZ)$_2$

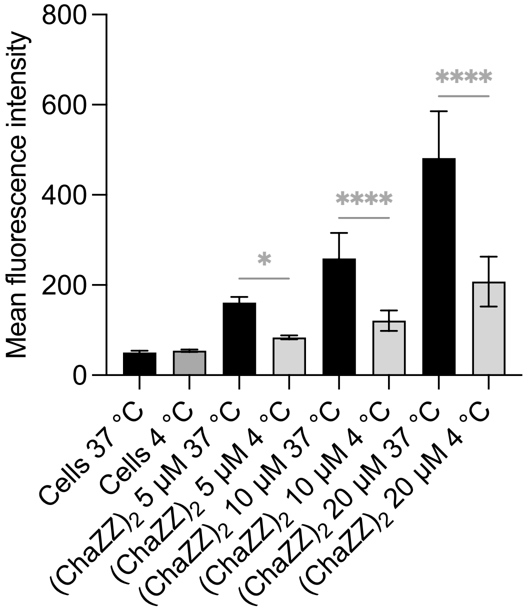

(ChaZZ)$_2$-Cha

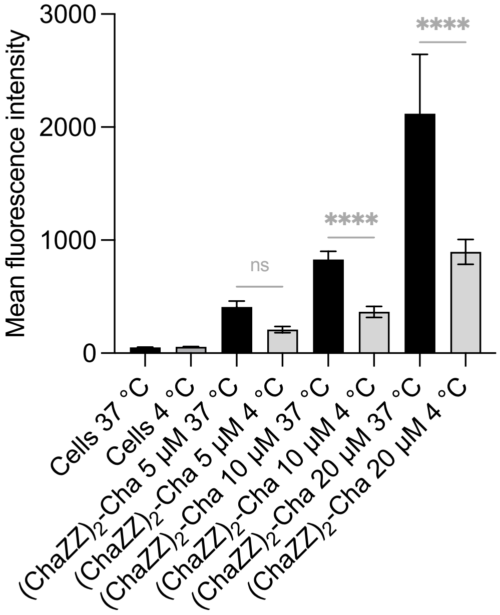

Figure S9. - continued

(ChProZZ)$_2$

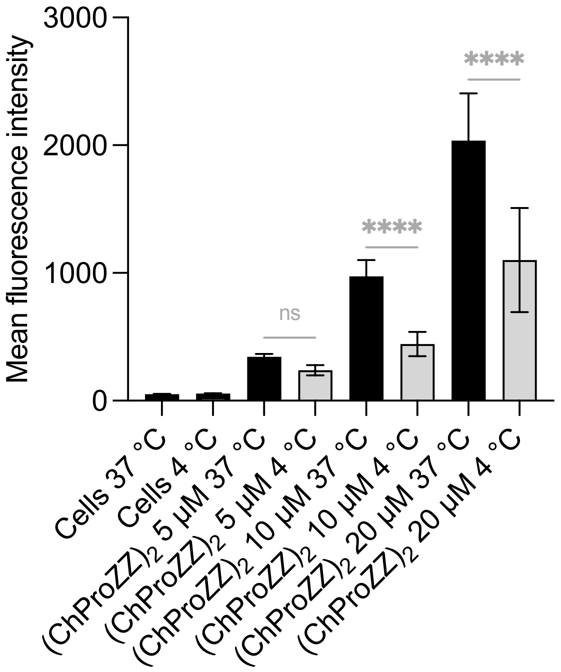

(ChProZZ)$_2$-ChPro

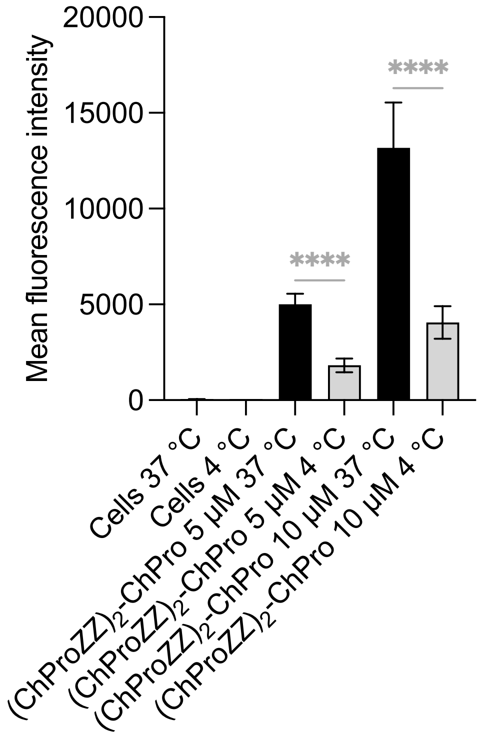

Figure S9. - continued

Analytical data

Peptides

CF-Ahx-VZZVZZVZZ (**(ValZZ)**$_3$)

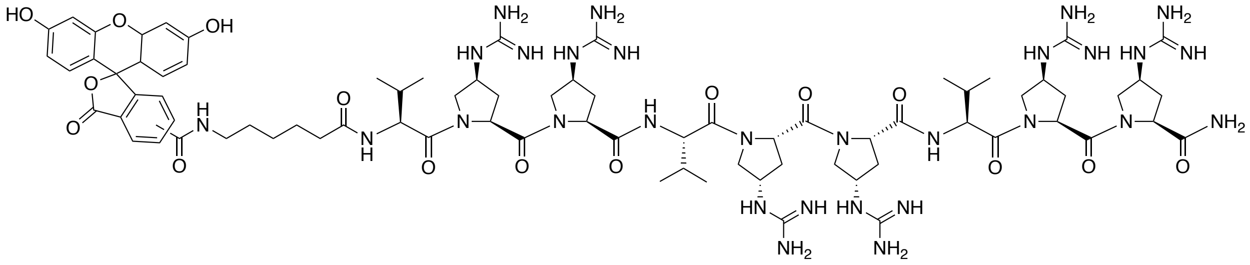

Purity, determined using conditions B.

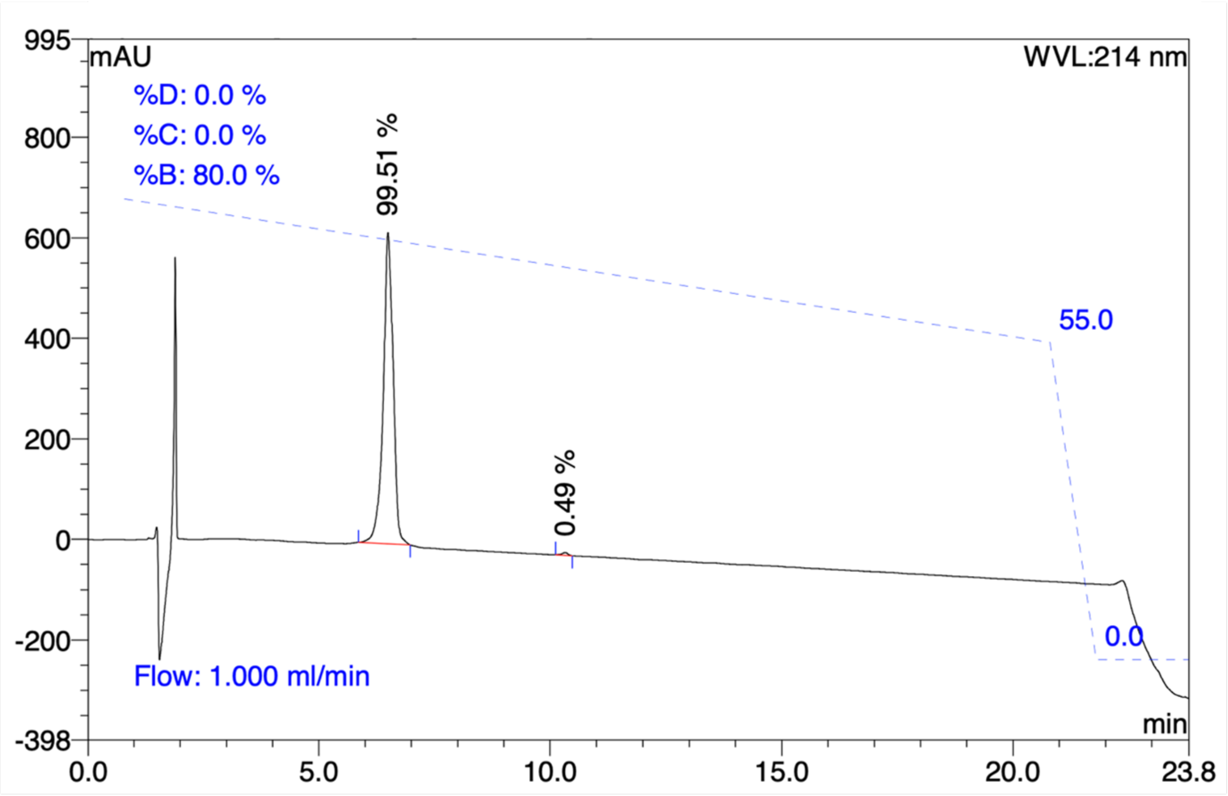

Retention time analysis, determined using conditions A.

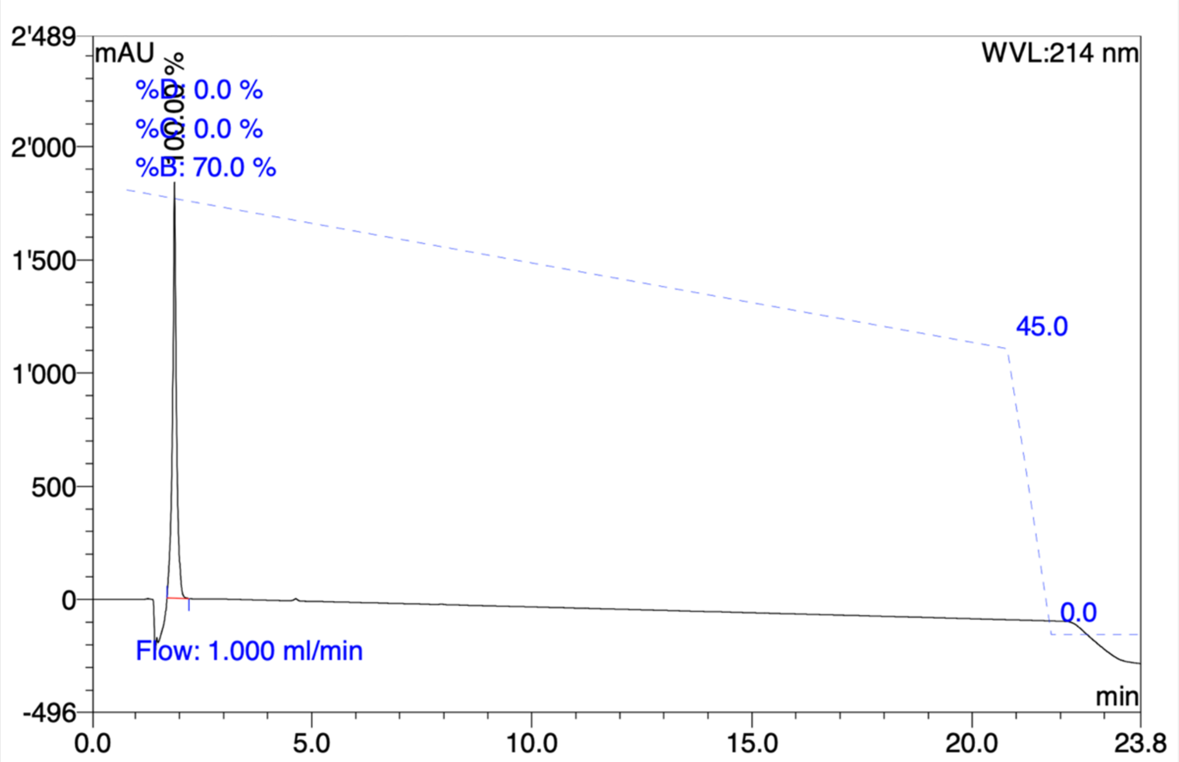

The peptide, being the least hydrophobic, is eluting together with the injection peak.

HR-MALDI m/z calcd for $C_{78}H_{113}N_{29}O_{16}$: 855.9455 $[M+H]^{2+}$; found: 855.9470 $[M+H]^{2+}$.

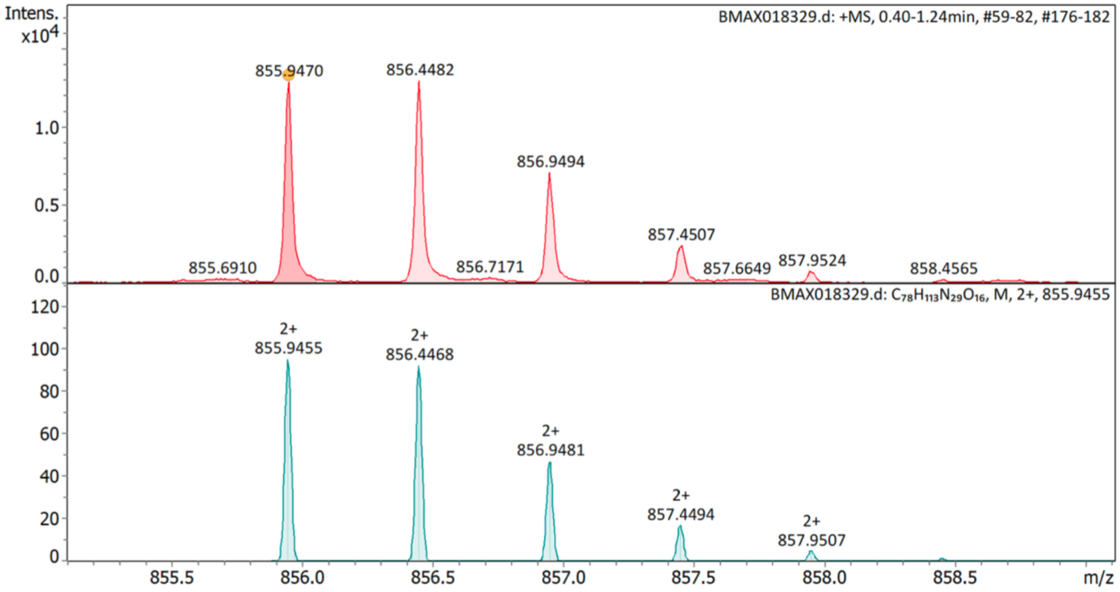

CF-Ahx-FZZFZZFZZ **((PheZZ)$_3$**

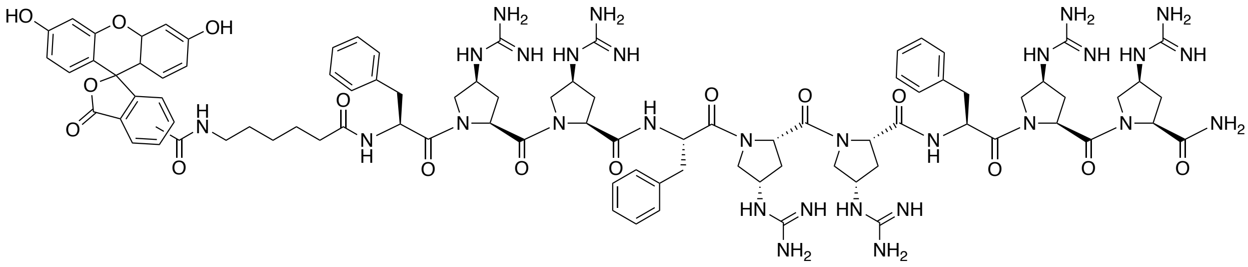

Purity, determined using conditions B.

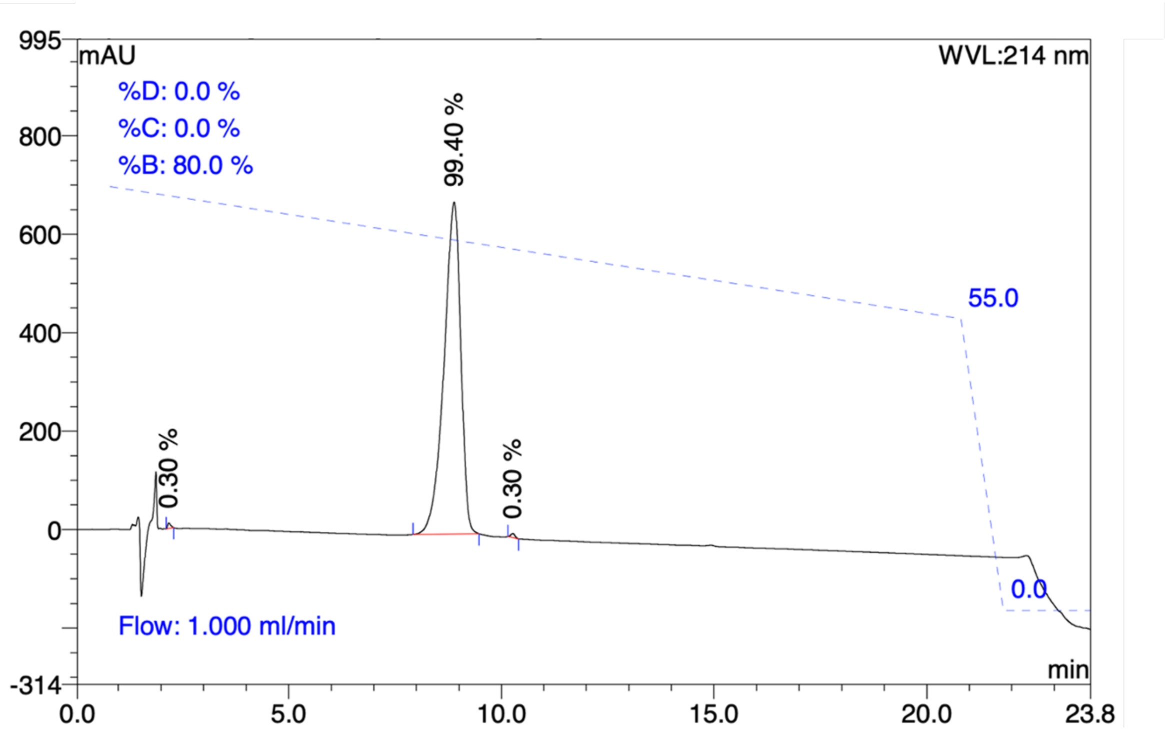

Retention time analysis, determined using conditions A.

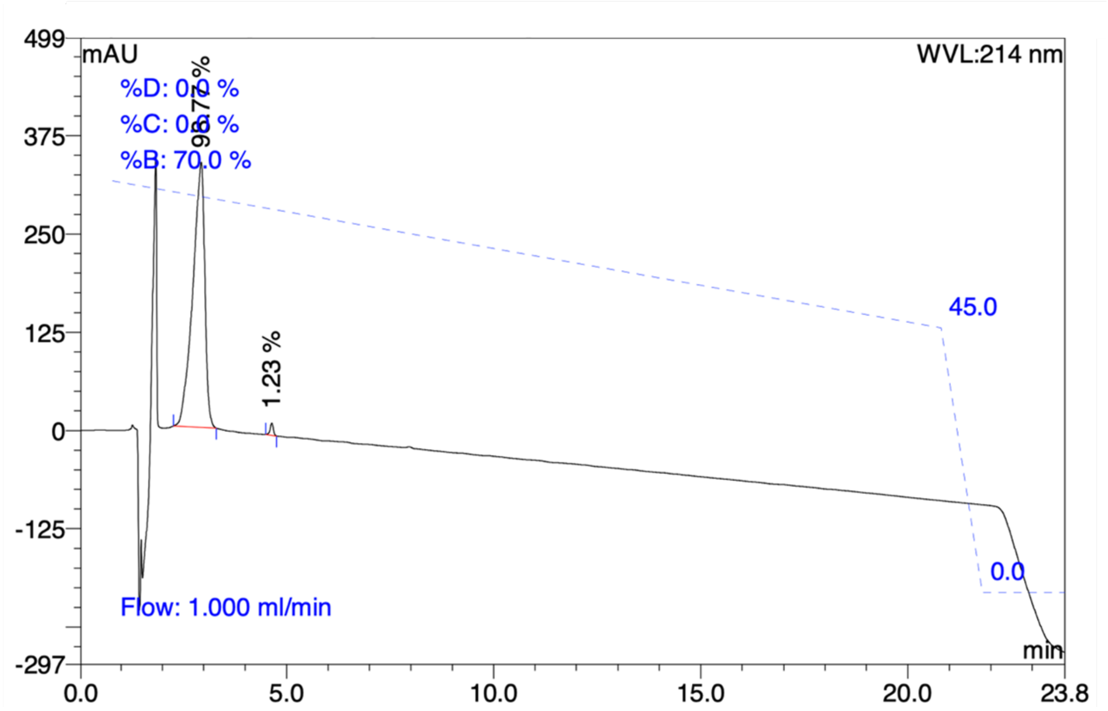

HR-MALDI m/z calcd for $C_{90}H_{113}N_{29}O_{16}$: 464.4764 $[M+H]^{4+}$; found: 464.4770 $[M+H]^{4+}$.

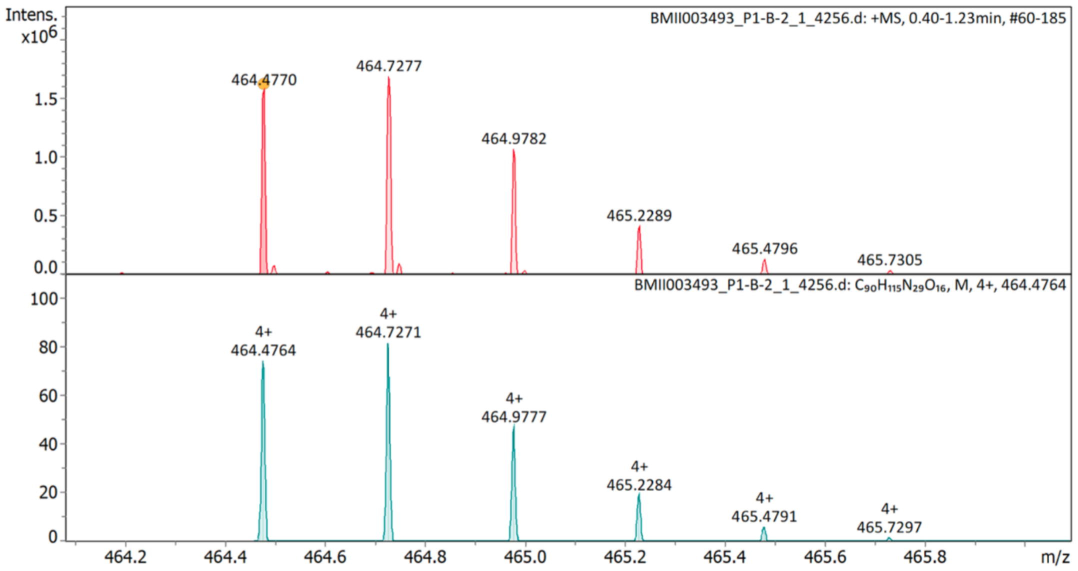

CF-Ahx-WZZWZZWZZ **((TrpZZ)$_3$**

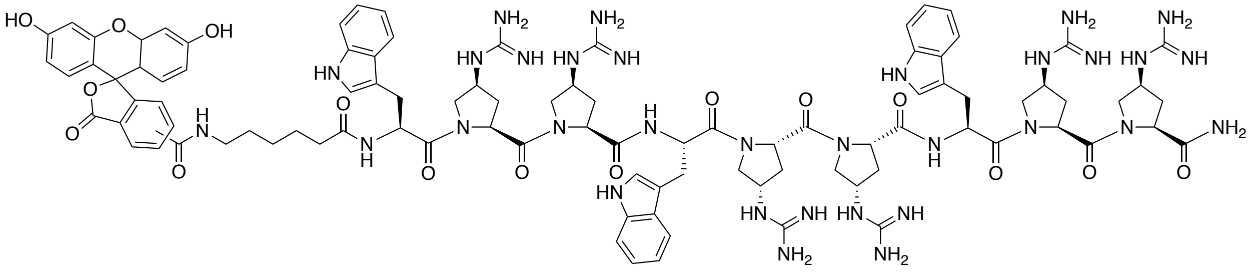

Purity, determined using conditions B.

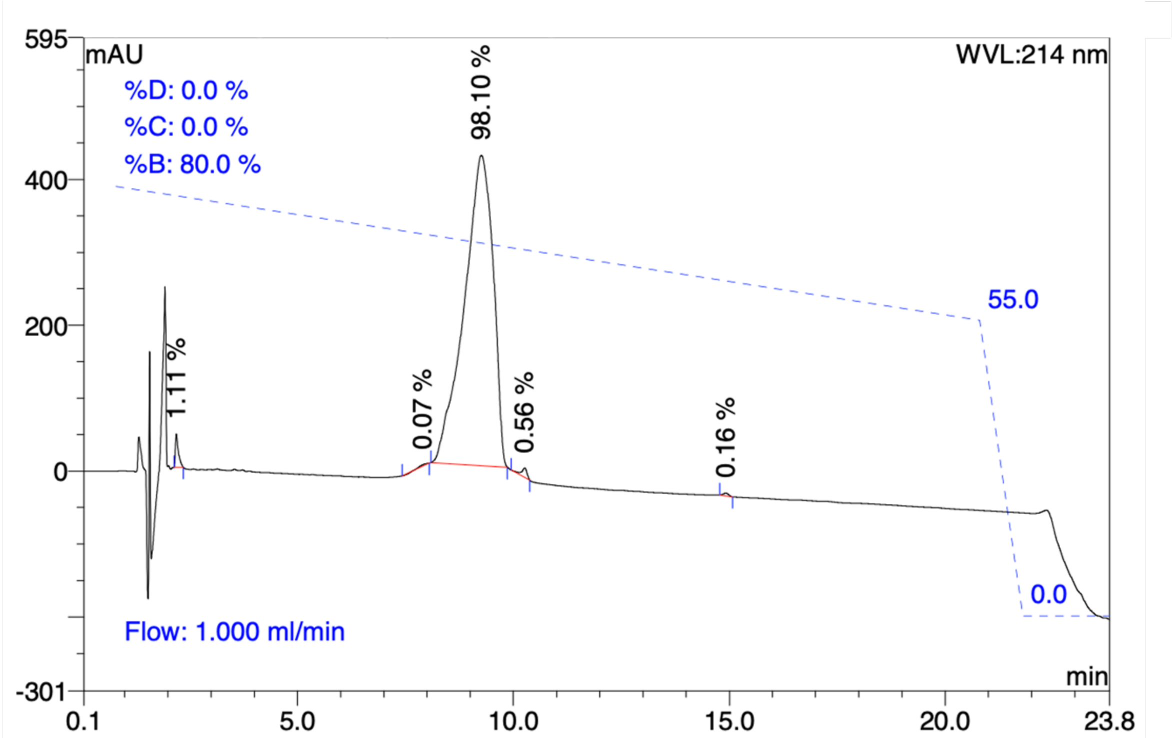

Retention time analysis, determined using conditions A.

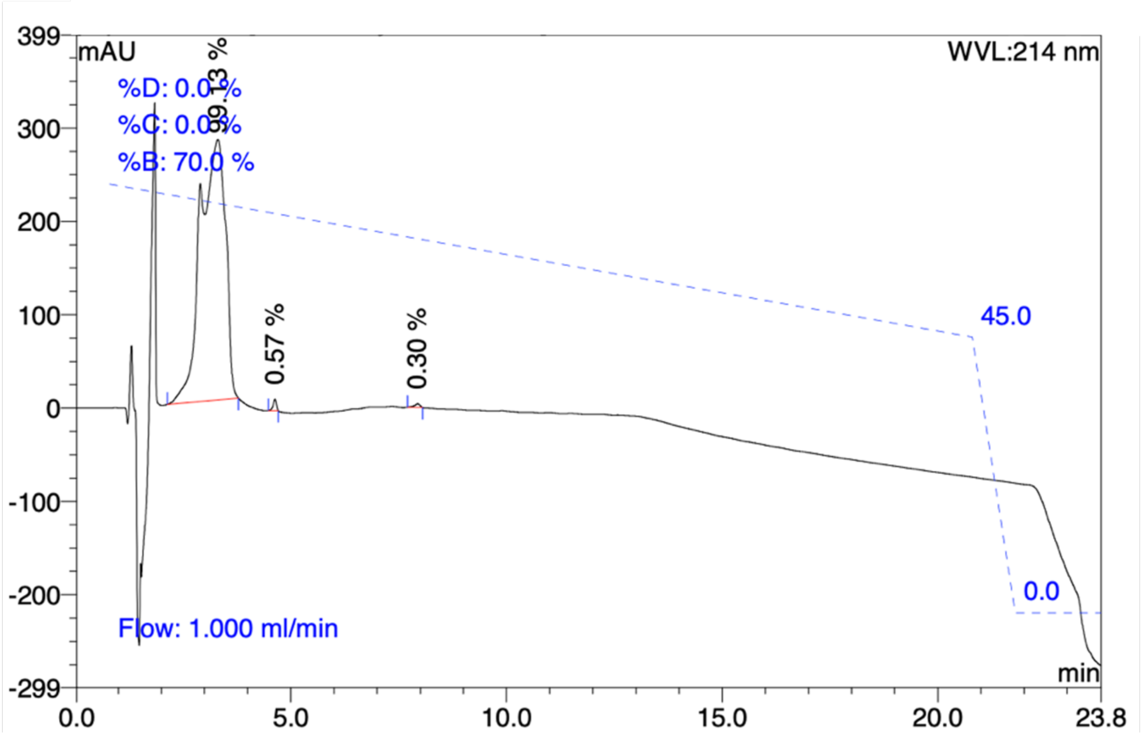

HR-MALDI m/z calcd for $C_{96}H_{116}N_{32}O_{16}$: 657.9770 $[M+H]^{3+}$; found: 657.9787 $[M+H]^{3+}$.

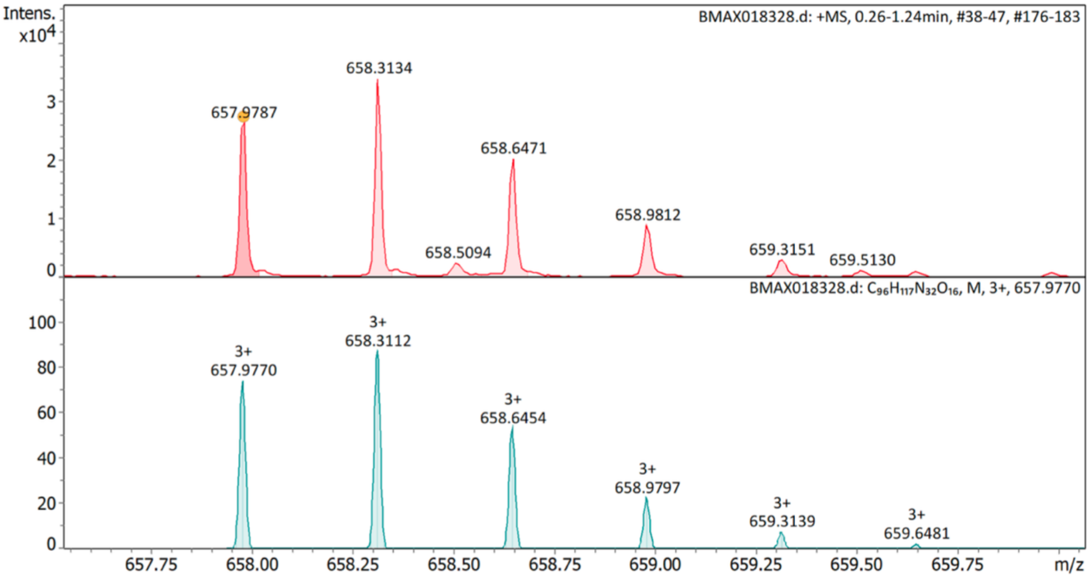

CF-Ahx-ChaZZChaZZChaZZ (**(ChaZZ)**$_3$)

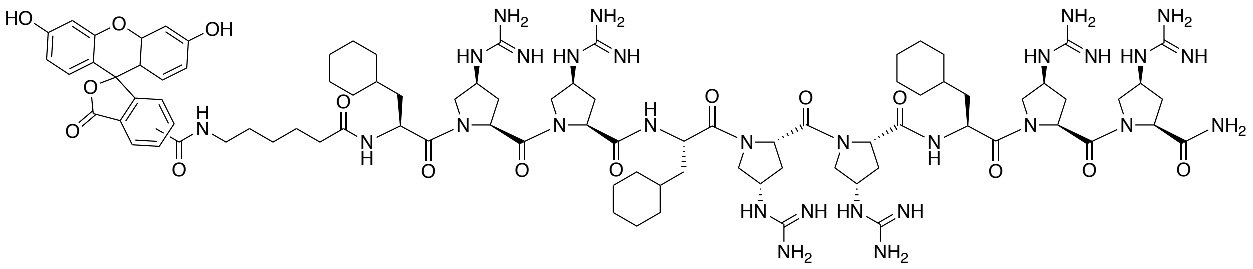

Purity and **retention time analysis,** determined using conditions A.

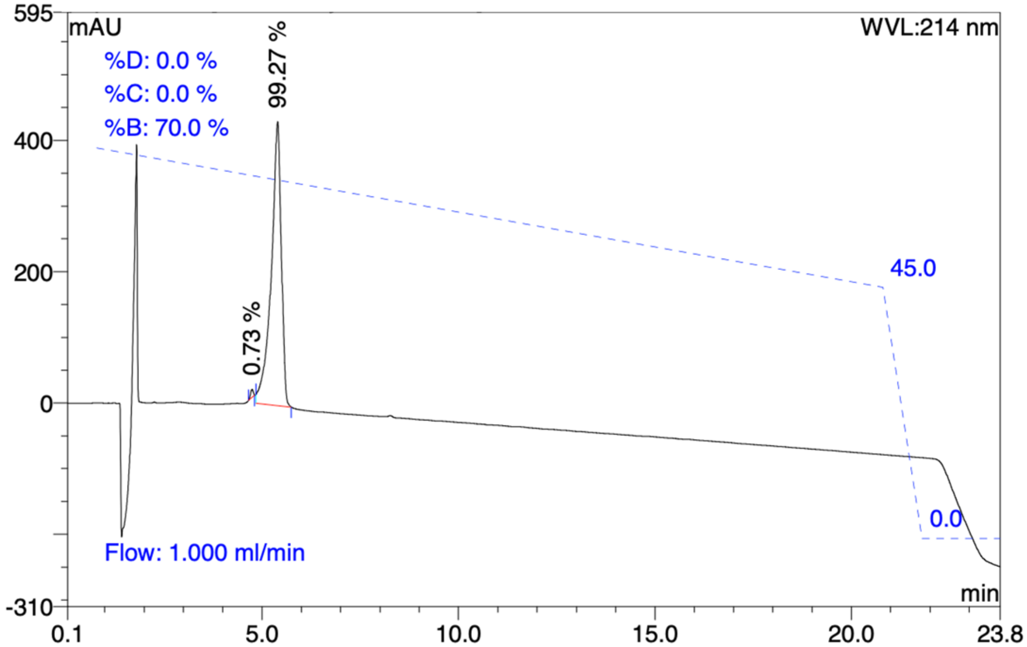

HR-MALDI m/z calcd for $C_{90}H_{129}N_{29}O_{16}$: 625. 013 $[M+H]^{3+}$; found: 625.0147 $[M+H]^{3+}$.

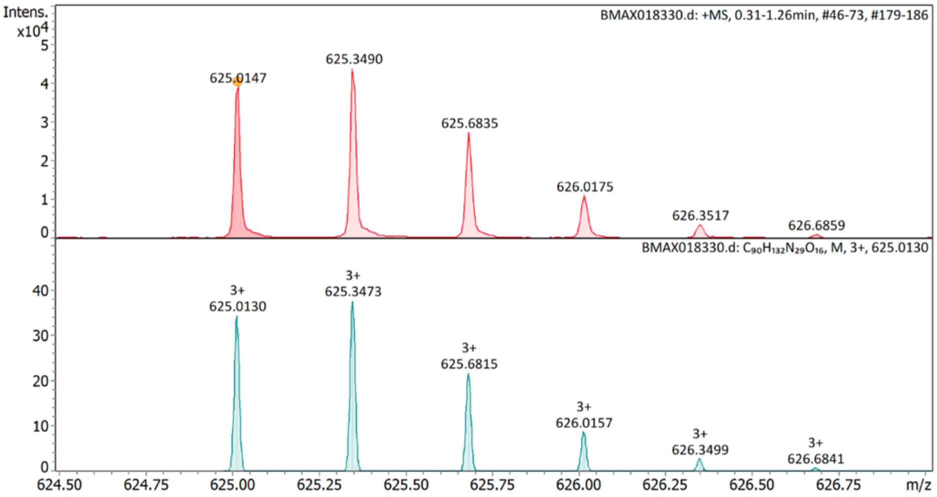

CF-Ahx-ChaZZChaZZChaZZCha (**(ChaZZ)$_3$-Cha**)

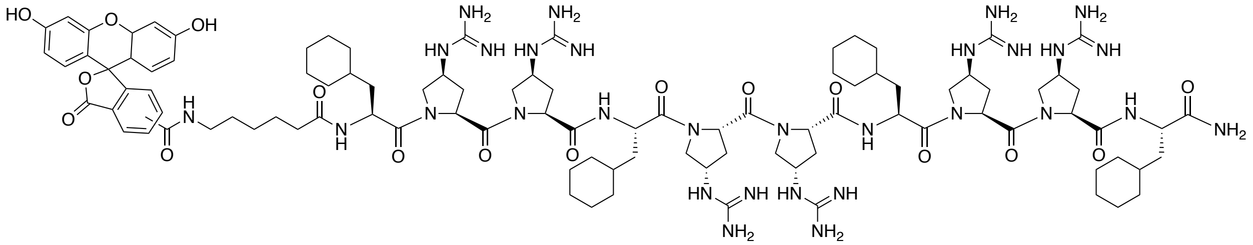

Purity and **retention time analysis,** determined using conditions A.

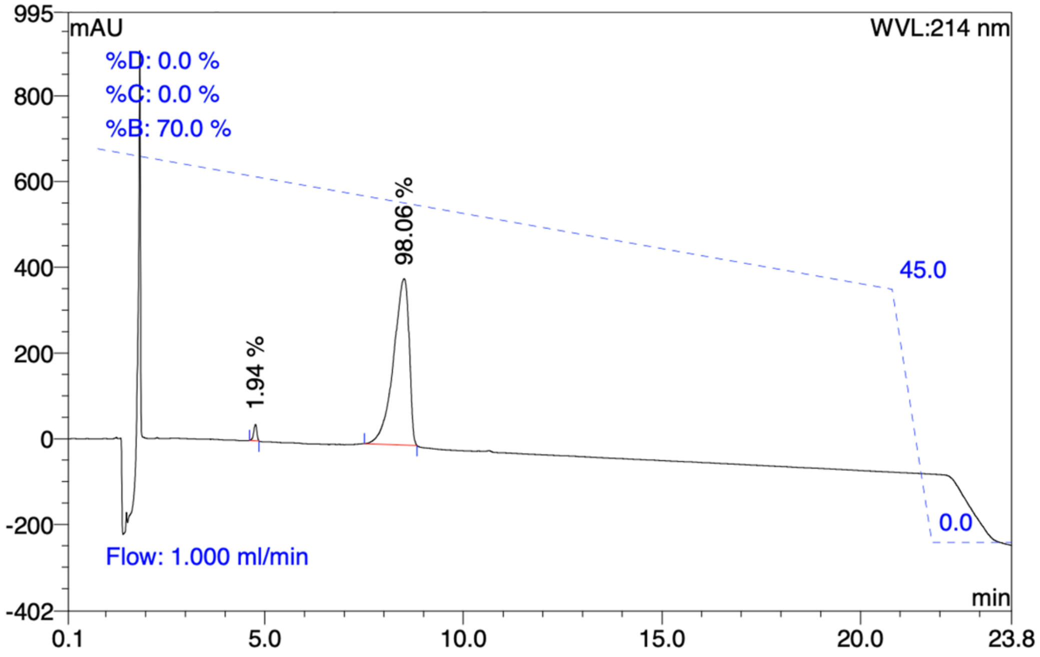

HR-MALDI m/z calcd for $C_{99}H_{146}N_{30}O_{17}$: 676.0515 $[M+H]^{3+}$; found: 676.0532 $[M+H]^{3+}$.

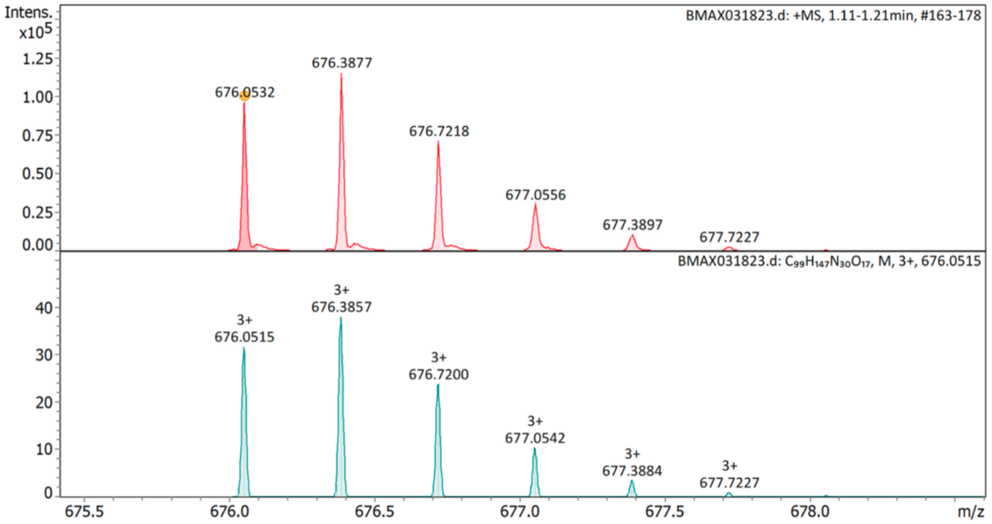

CF-Ahx-ChaZZChaZZ (**(ChaZZ)$_2$**)

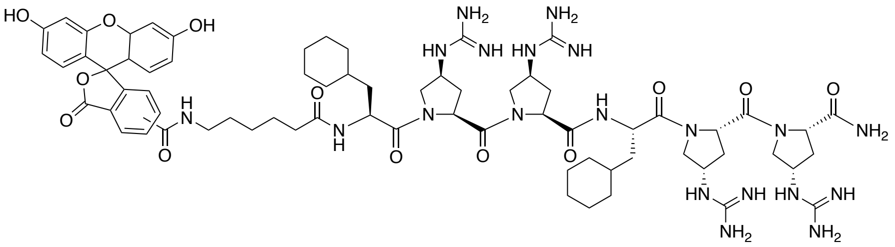

Purity and **retention time analysis,** determined using conditions A.

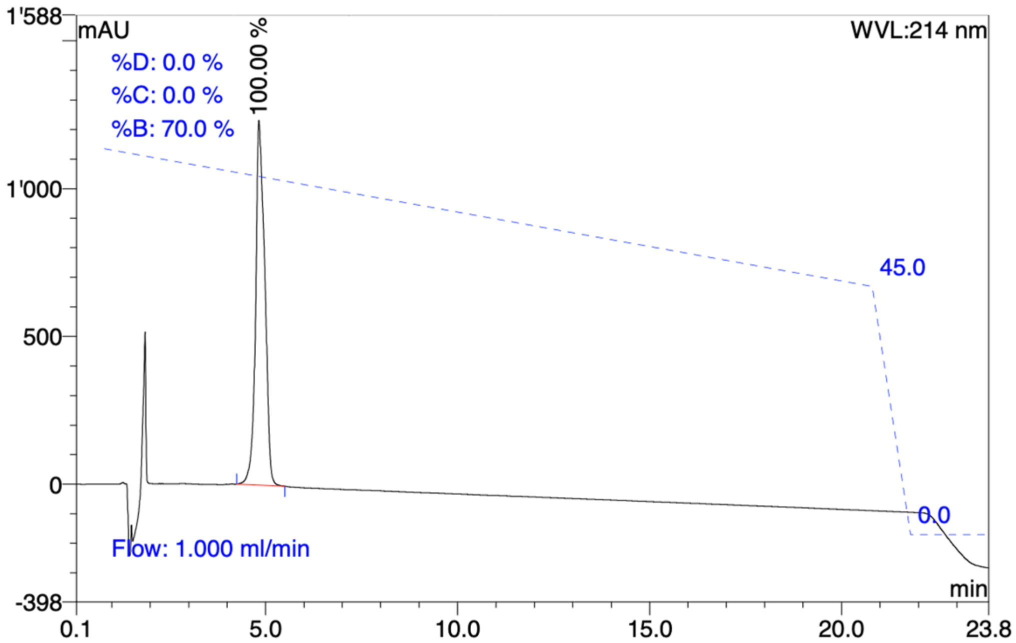

HR-MALDI m/z calcd for $C_{69}H_{96}N_{20}O_{13}$: 706.3735 $[M+H]^{2+}$; found: 706.3735 $[M+H]^{2+}$.

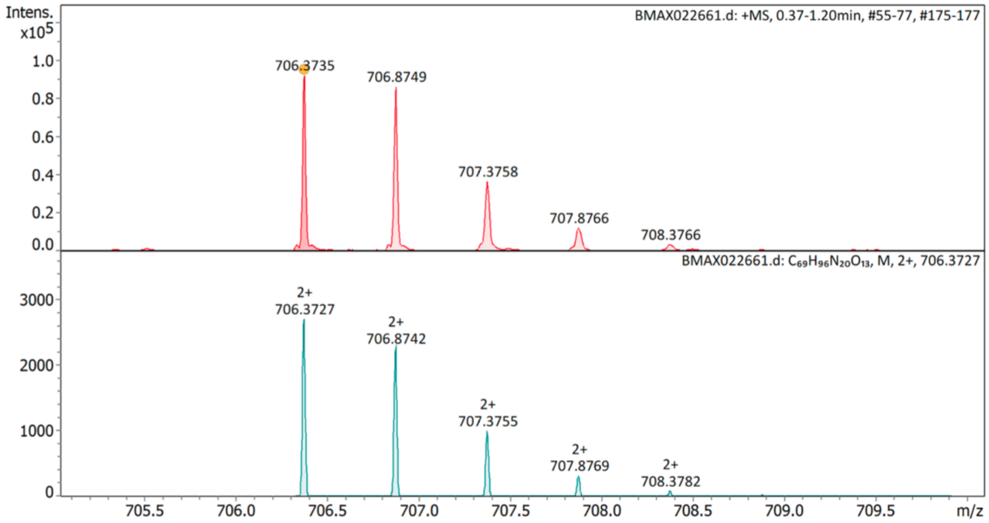

CF-Ahx-ChaZZChaZZCha (**(ChaZZ)₂-Cha**)

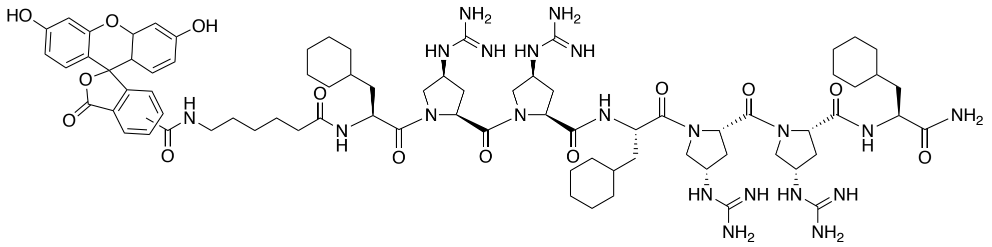

Purity and **retention time analysis,** determined using conditions A.

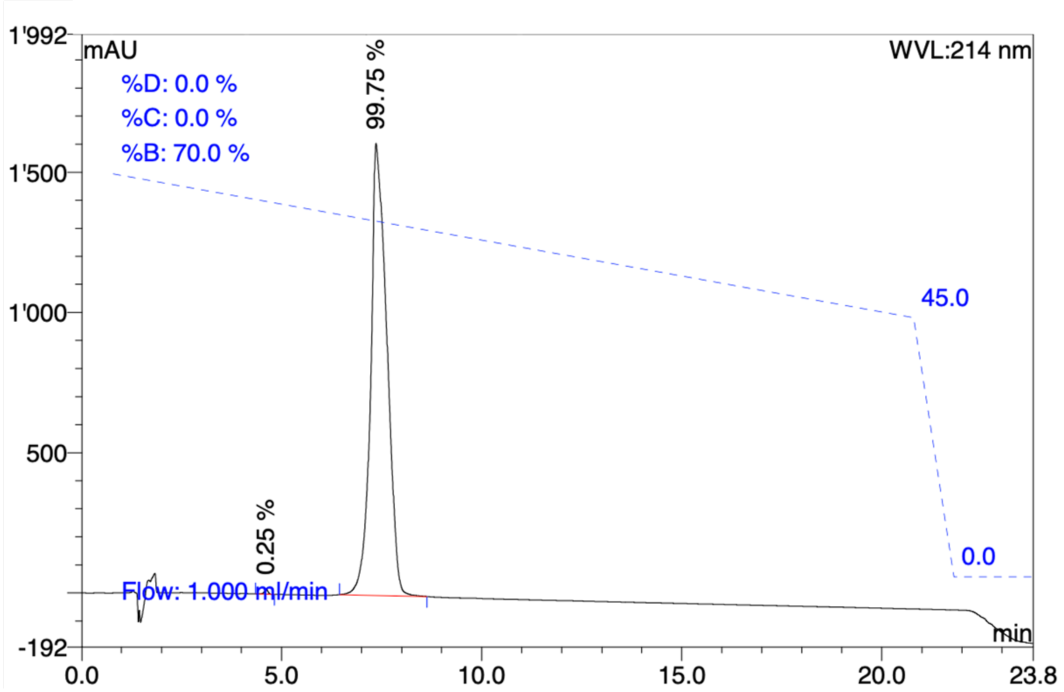

HR-MALDI m/z calcd for $C_{78}H_{111}N_{21}O_{14}$: 782.9304 $[M+H]^{2+}$; found: 782.9316 $[M+H]^{2+}$.

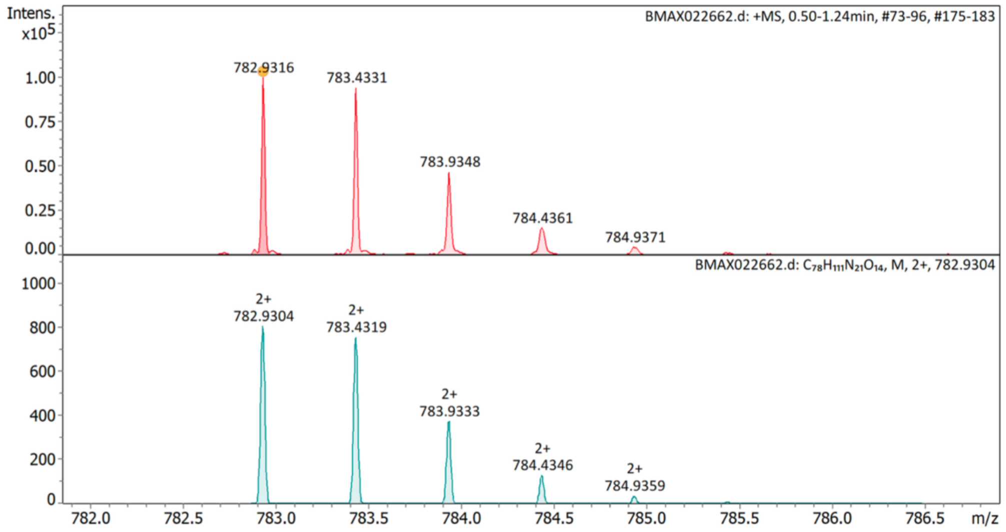

CF-Ahx-ChProZZChProZZChProZZ (**(ChProZZ)$_3$**)

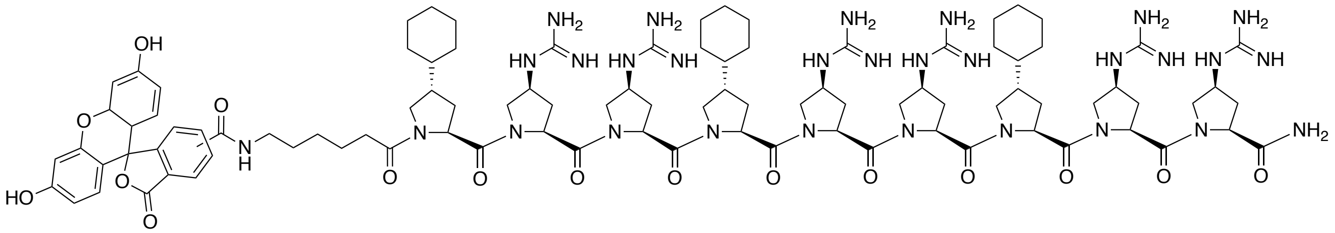

Purity and **retention time analysis,** determined using conditions A.

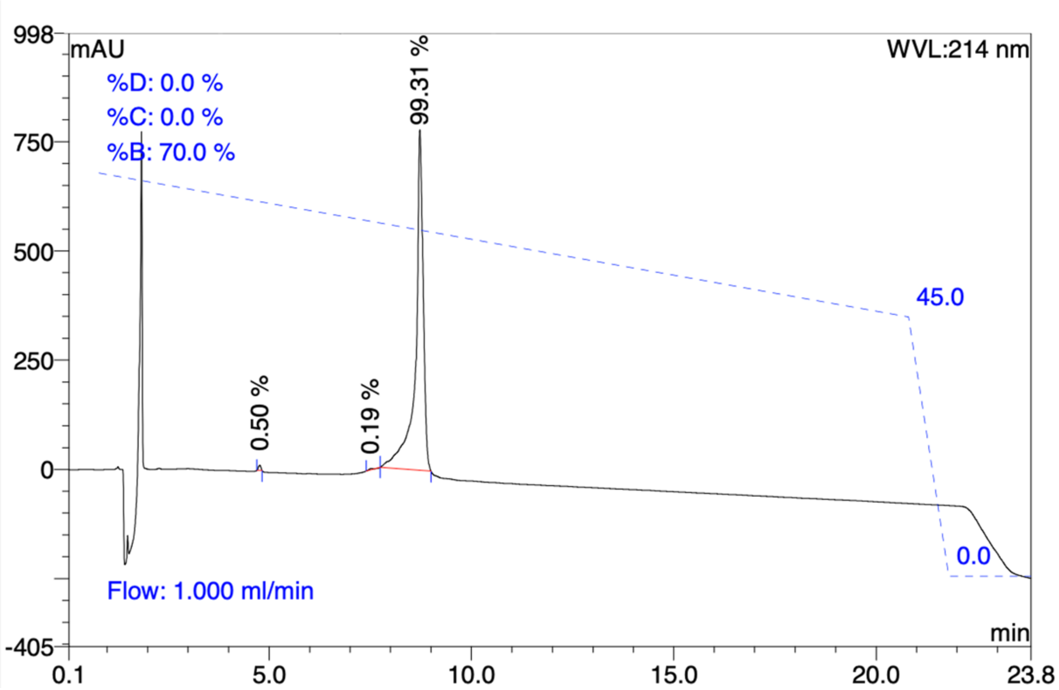

HR-MALDI m/z calcd for $C_{96}H_{137}N_{29}O_{16}$: 976.0394 $[M+H]^{2+}$; found: 976.0402 $[M+H]^{2+}$.

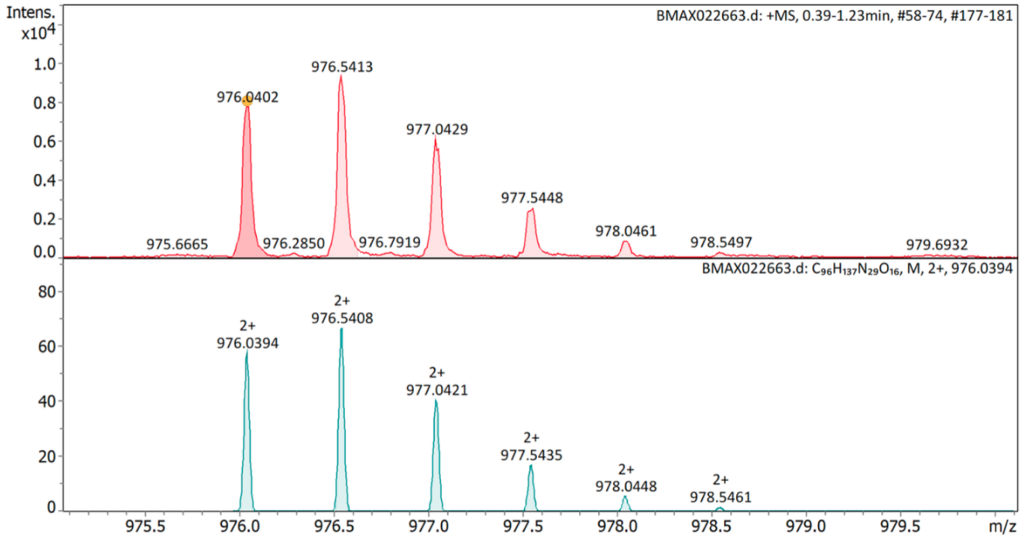

CF-Ahx-ChProZZChProZZChProZZChPro **((ChProZZ)3-ChPro)**

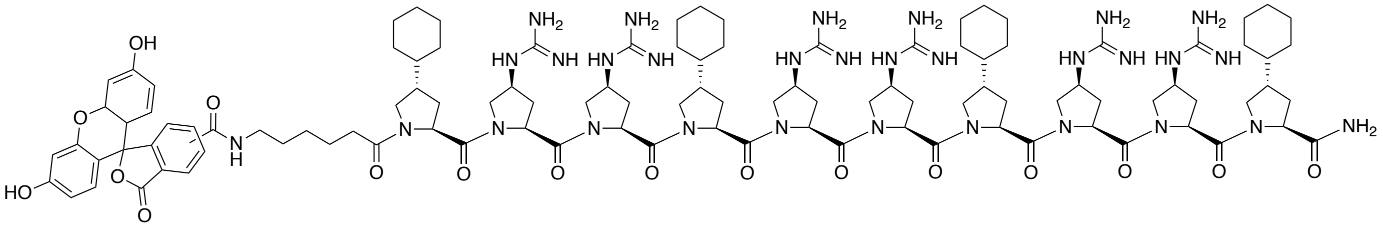

Purity and **retention time analysis,** determined using conditions A.

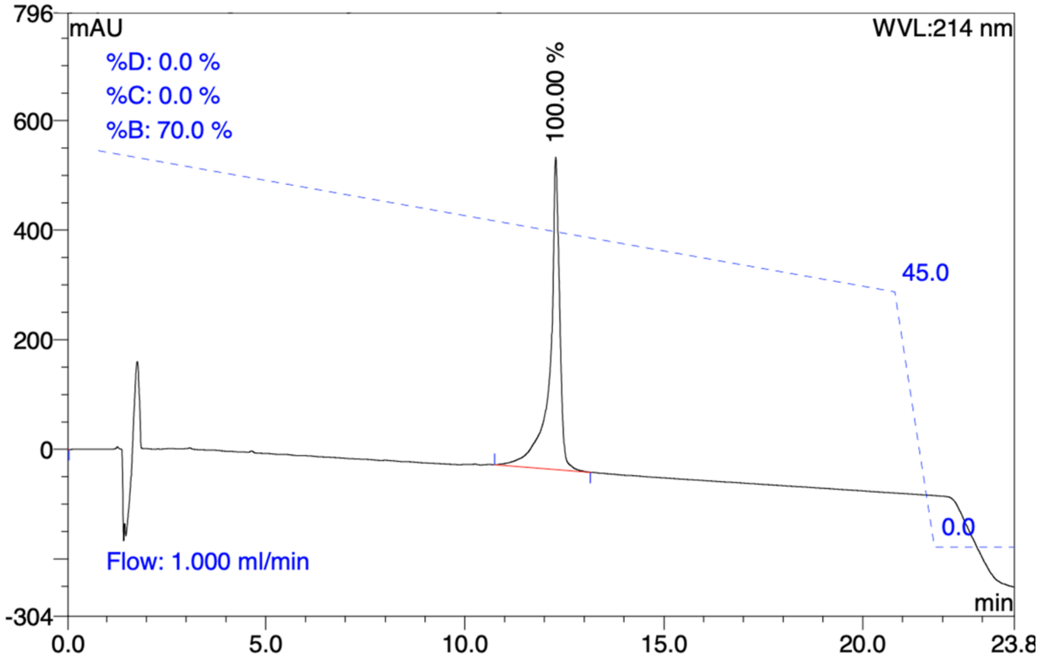

HR-MALDI m/z calcd for $C_{107}H_{154}N_{30}O_{17}$: 710.7390 $[M+H]^{3+}$; found: 710.7405 $[M+H]^{3+}$.

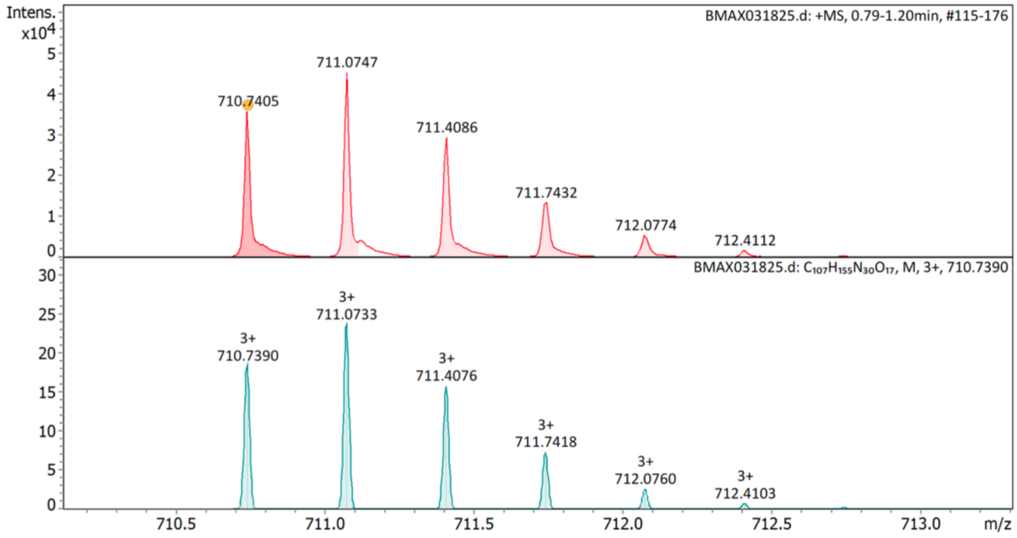

CF-Ahx-ChProZZChProZZ (**(ChProZZ)₂**)

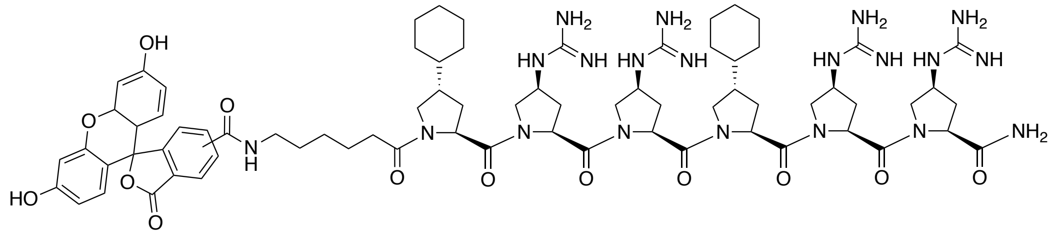

Purity and **retention time analysis,** determined using conditions A.

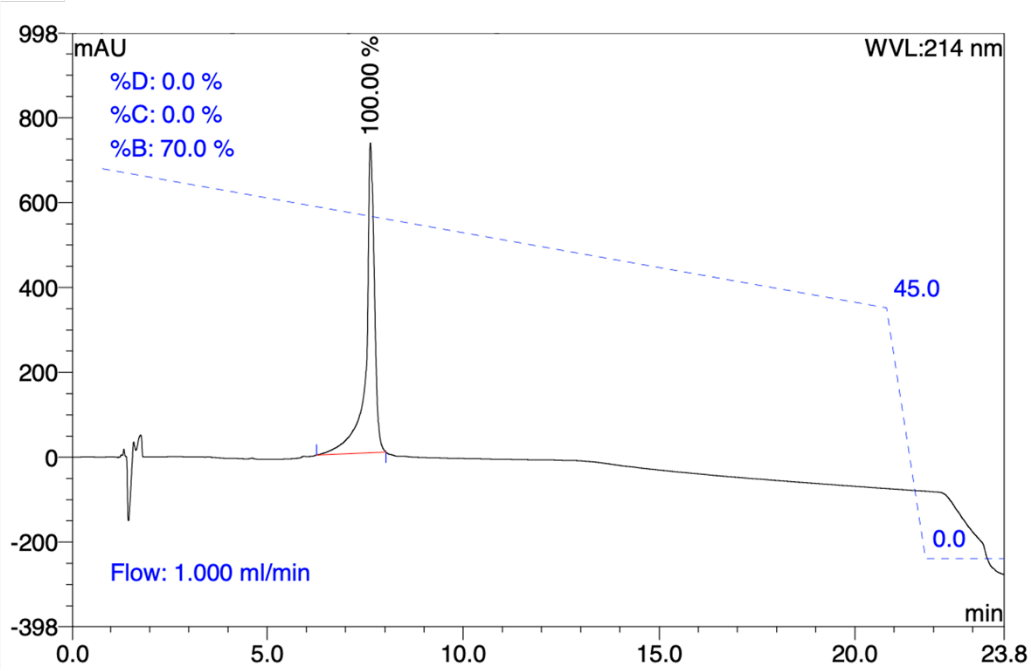

HR-MALDI m/z calcd for $C_{73}H_{100}N_{20}O_{13}$: 732.3884 $[M+H]^{2+}$; found: 732.3891 $[M+H]^{2+}$.

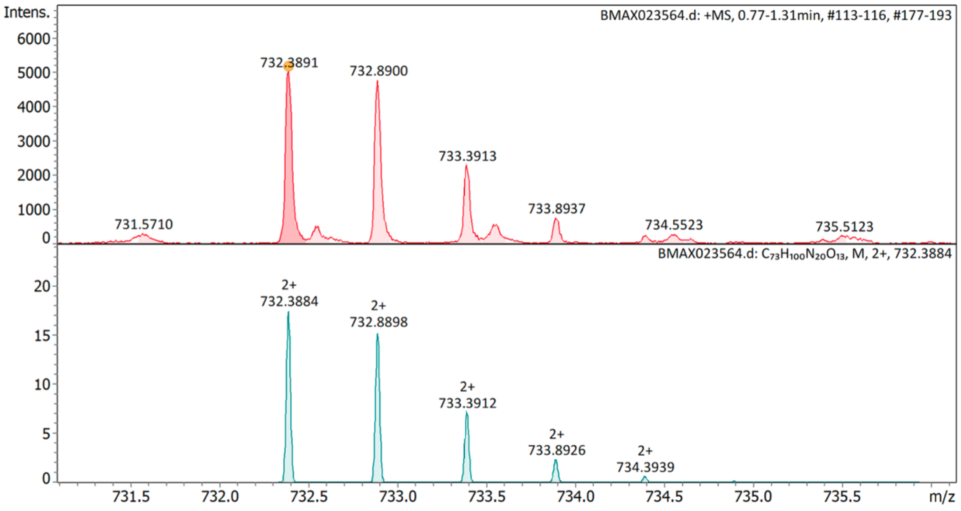

CF-Ahx-ChProZZChProZZChPro **((ChProZZ)$_2$-ChPro)**

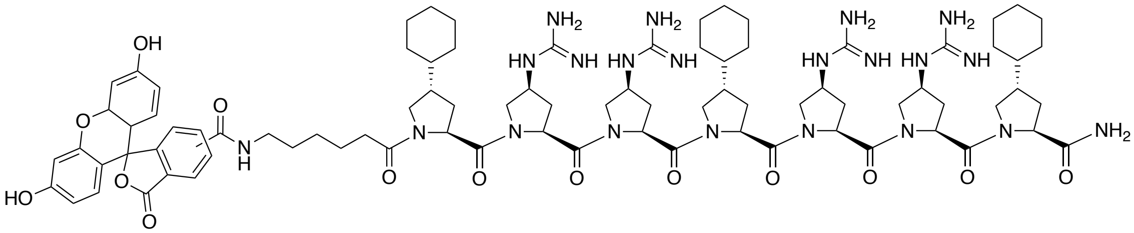

Purity and **retention time analysis,** determined using conditions A.

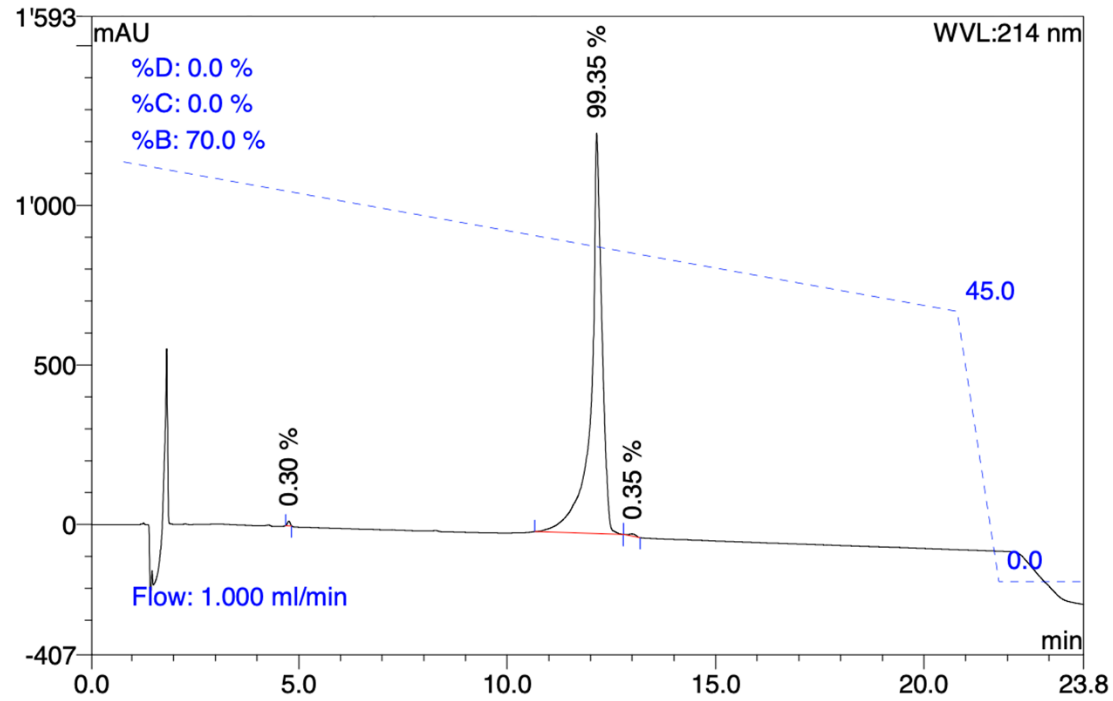

HR-MALDI m/z calcd for $C_{84}H_{117}N_{21}O_{14}$: 821.9539 $[M+H]^{2+}$; found: 821.9540 $[M+H]^{2+}$.

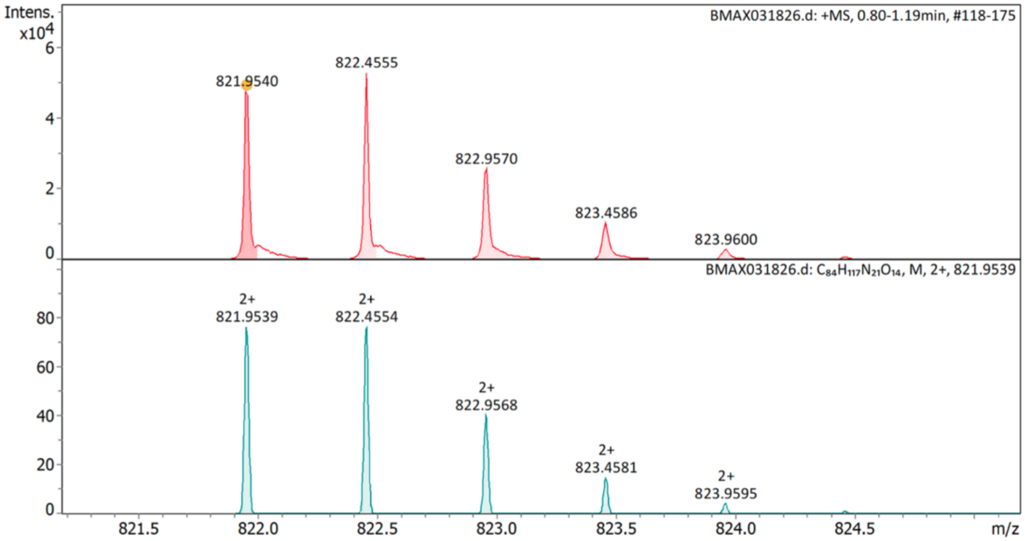

CF-Ahx-ChaRRChaRRChaRR **((ChaRR)3)**

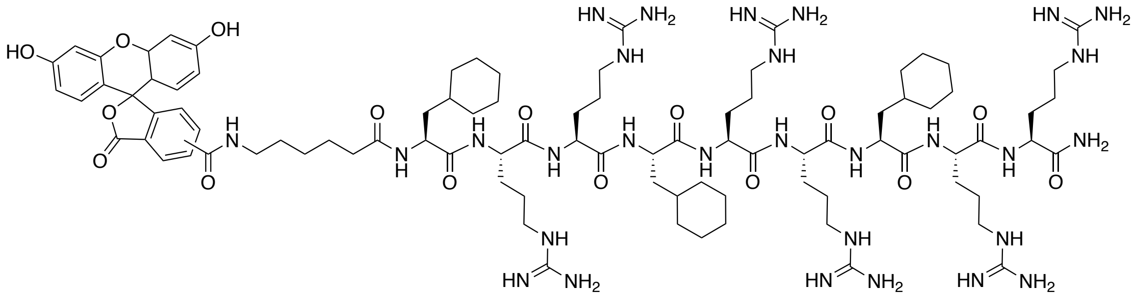

Purity and **retention time analysis,** determined using conditions A.

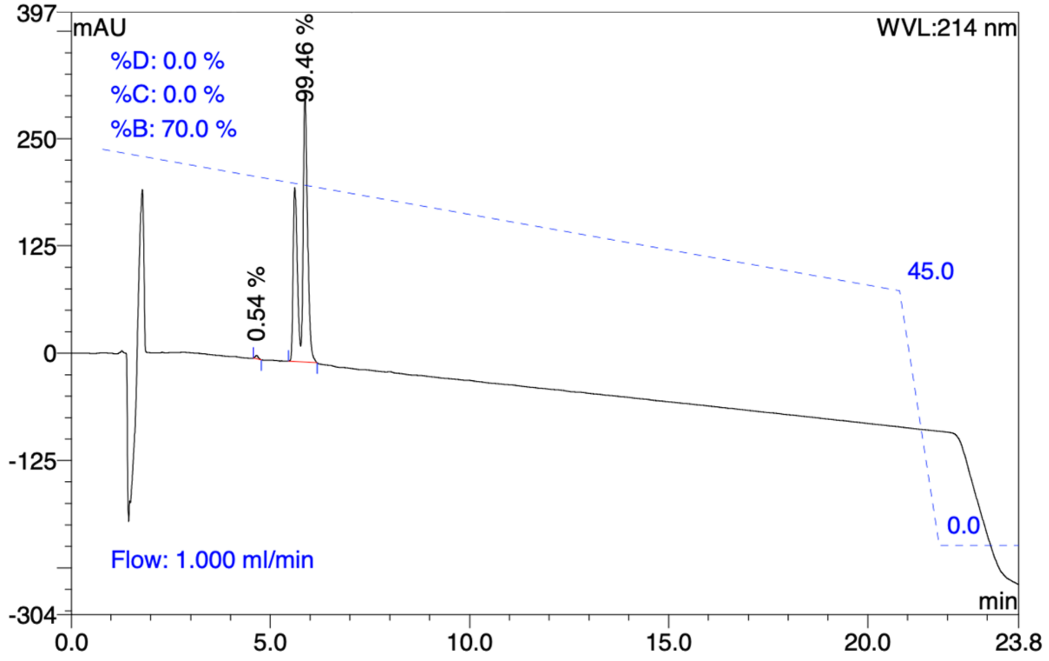

The double peak likely arises the isomers of 5(6)-carboxyfluorescein.

HR-MALDI m/z calcd for $C_{90}H_{143}N_{29}O_{16}$: 629.0443 $[M+H]^{3+}$; found: 629.0455 $[M+H]^{3+}$.

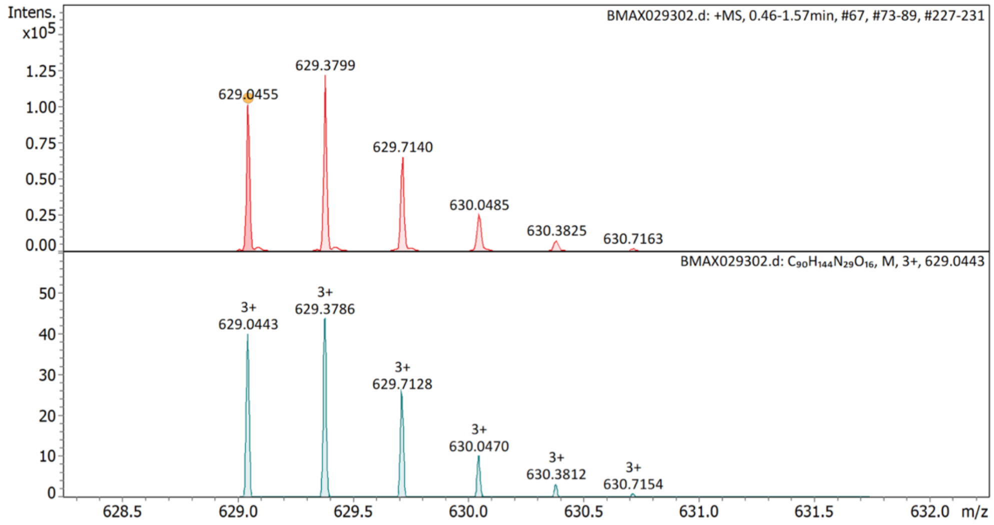

Fmoc-(4*S*)Gup(Boc)$_2$-OH

(2*S*,4*S*)-1-(((9*H*-fluoren-9-yl)methoxy)carbonyl)-4-((*Z*)-2,3-bis(*tert*-butoxycarbonyl)-guanidino)pyrrolidine-2-carboxylic acid (**Fmoc-(4*S*)Gup(Boc)$_2$-OH**)

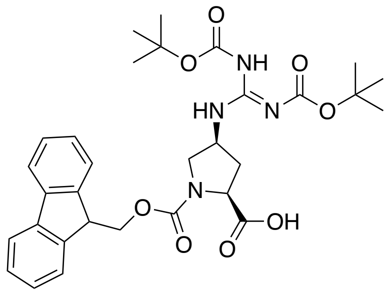

Fmoc-(4*S*)Gup(Boc)$_2$-OH was synthesized according to a procedure previously reported by our group.[3]

^{1}H and ^{13}C NMR show a double set of peaks due to *cis* and *trans* conformers around the tertiary carbamate in a ratio of 1.25:1.

^{1}H NMR (500 MHz, DMSO) δ 12.92 (s, 1H; OH), 11.42 (d, *J* = 7.5 Hz, 1H; NH), 8.44 (dd, *J* = 15.5, 7.6 Hz, 1H; NH), 7.90 (t, *J* = 2.5 Hz, 2H; Fmoc), 7.66 (dd, *J* = 7.5, 3.5 Hz, 2H; Fmoc), 7.42 (td, *J* = 7.5, 1.1 Hz, 2H; Fmoc), 7.37 – 7.30 (m, 2H; Fmoc), 4.70 – 4.57 (m, 1H, Hγ), 4.40 (dd, *J* = 9.4, 3.9 Hz, 1H, Hα), 4.34 – 4.12 (m, 3H, Fmoc), 3.79 (dt, *J* = 11.2, 6.0 Hz, 1H; Hδ), 3.43 – 3.27 (m, 3H; Hδ), 2.70 – 2.53 (m, 1H; Hβ), 2.01 (ddt, *J* = 47.2, 13.5, 4.3 Hz, 1H; Hβ), 1.97 (dt, *J* = 13.4, 4.3 Hz, 1H; Hβ), 1.47 (s, 9H), 1.41 (d, *J* = 4.9 Hz, 9H).

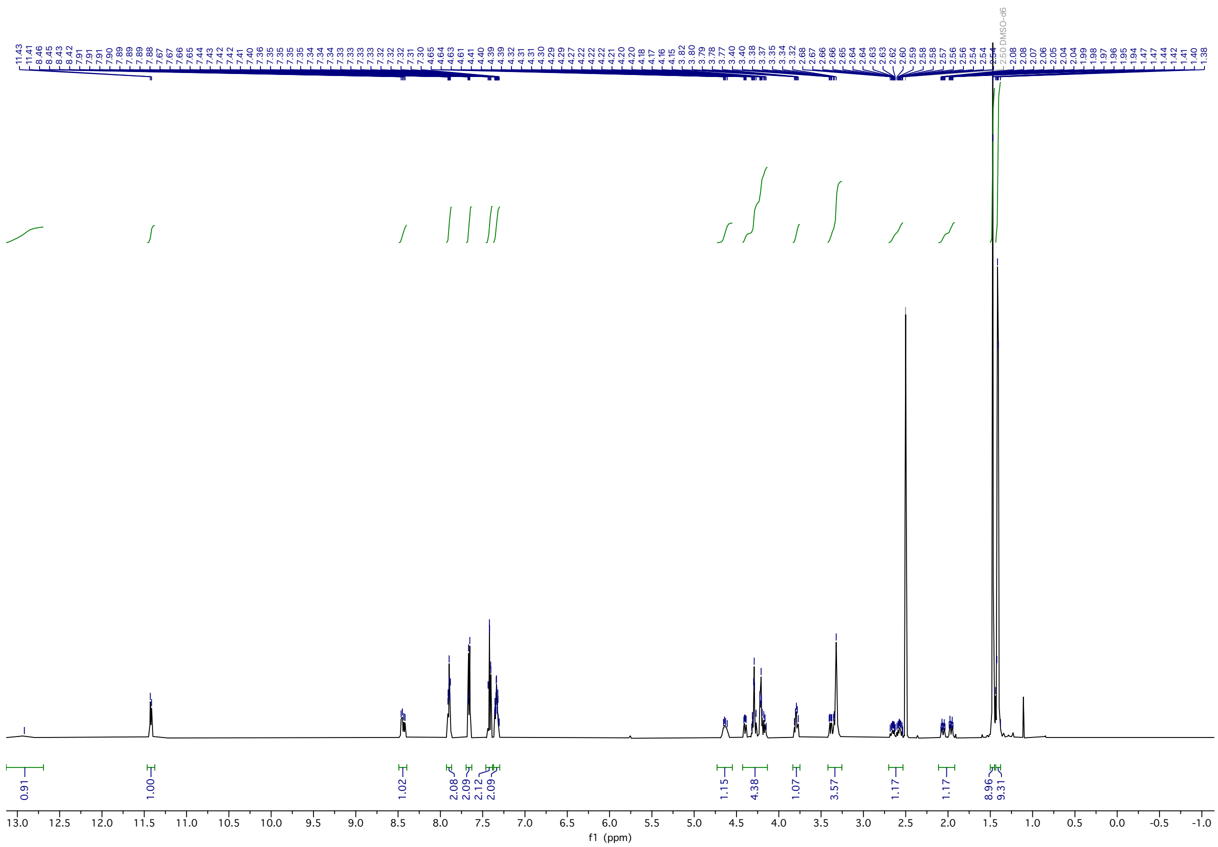

^{13}C NMR (126 MHz, DMSO) δ 174.1 (CO), 173.6 (CO), 162.9 (CO), 154.8 (CO), 153.7 (CO), 151.7 (Guanidinium), 143.7 (Fmoc), 140.7 (Fmoc), 127.7 (Fmoc), 127.7 (Fmoc), 127.2 (Fmoc), 125.2 (Fmoc), 120.1 (Fmoc), 83.0 (^{t}Bu), 78.5 (^{t}Bu), 67.2 (Fmoc), 66.8 (Fmoc), 57.7 (Hα), 57.3 (Hα), 52.4 (Cδ), 51.8 (Cδ), 49.4 (Cγ), 48.5 (Cγ), 46.7 (Fmoc), 46.5 (Fmoc), 36.0 (Cβ), 34.6 (Cβ), 28.0 (^{t}Bu), 27.6 (^{t}Bu).

Analytical data are in agreement with previous reports.[4,5]

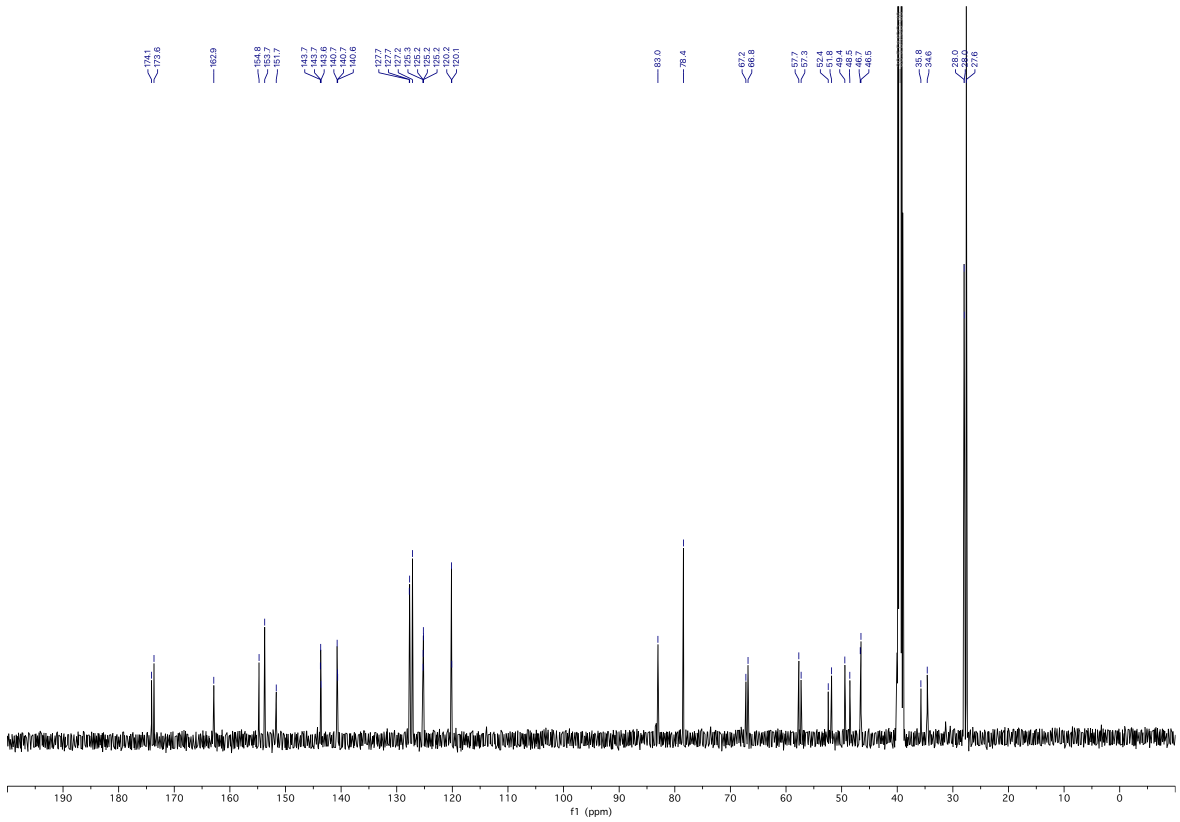

References

(1) Bolte, S.; Cordelieres, F. P., A guided tour into subcellular colocalization analysis in light microscopy. *J. Microsc*. **2006**, *22,* 213-232.
(2) Auer, H. E. Far-ultraviolet absorption and circular dichroism spectra of L-tryptophan and some derivatives. *J. Am. Chem. Soc.***1973,** *95*, 3003-3011.
(3) Nagel, Y. A.; Raschle, P. S.; Wennemers, H. Effect of Preorganized Charge-Display on the Cell-Penetrating Properties of Cationic Peptides. *Angew. Chem. Int. Ed.* **2017**, *129*, 128-132.
(4) Tamaki, M.; Han, G.; Hruby, V. J. Practical and efficient synthesis of orthogonally protected constrained 4- guanidinoprolines. *J. Org. Chem*. **2001**, *66*, 1038-1042.
(5) Feichtinger, H. L.; Sings, T. J.; Baker, K.; Matthews, M.; Goodmann, *J. Org. Chem*. **1998**, *63*, 8432-8439.
